# Hot spots policing of small geographic areas effects on crime

**DOI:** 10.1002/cl2.1046

**Published:** 2019-09-08

**Authors:** Anthony A. Braga, Brandon Turchan, Andrew V. Papachristos, David M. Hureau

**Affiliations:** ^1^ School of Criminology and Criminal Justice Northeastern University Boston Massachusetts; ^2^ Department of Sociology Northwestern University Evanston Illinois; ^3^ School of Criminal Justice State University of New York Albany New York

## Abstract

**Background:**

In recent years, crime scholars and practitioners have pointed to the potential benefits of focusing crime prevention efforts on crime places. A number of studies suggest that there is significant clustering of crime in small places, or “hot spots,” that generate half of all criminal events. Researchers have argued that many crime problems can be reduced more efficiently if police officers focused their attention to these deviant places. The appeal of focusing limited resources on a small number of high‐activity crime places is straightforward. If crime can be prevented at these hot spots, then citywide crime totals could be reduced.

**Objectives:**

To assess the effects of focused police crime prevention interventions at crime hot spots. The review also examined whether focused police actions at specific locations result in crime displacement (i.e., crime moving around the corner) or diffusion (i.e., crime reduction in surrounding areas) of crime control benefits.

**Search Methods:**

A keyword search was performed on 15 abstract databases. Bibliographies of past narrative and empirical reviews of literature that examined the effectiveness of police crime control programs were reviewed and forward searches for works that cited seminal hot spots policing studies were performed. Bibliographies of past completed Campbell systematic reviews of police crime prevention efforts were reviewed and hand searches of leading journals in the field were completed. Experts in the field were consulted and relevant citations were obtained.

**Selection Criteria:**

To be eligible for this review, interventions used to control crime hot spots were limited to police‐led prevention efforts. Suitable police‐led crime prevention efforts included traditional tactics such as directed patrol and heightened levels of traffic enforcement as well as alternative strategies such as aggressive disorder enforcement and problem‐oriented policing. Studies that used randomized controlled experimental or quasiexperimental designs were selected. The units of analysis were limited to crime hot spots or high‐activity crime “places” rather than larger areas such as neighborhoods. The control group in each study received routine levels of traditional police crime prevention tactics.

**Data Collection and Analysis:**

Sixty‐five studies containing 78 tests of hot spots policing interventions were identified and full narratives of these studies were reported. Twenty‐seven of the selected studies used randomized experimental designs and 38 used quasiexperimental designs. A formal meta‐analysis was conducted to determine the crime prevention effects in the eligible studies. Random effects models were used to calculate mean effect sizes.

**Results:**

Sixty‐two of 78 tests of hot spots policing interventions reported noteworthy crime and disorder reductions. The meta‐analysis of key reported outcome measures revealed a small statistically significant mean effect size favoring the effects of hot spots policing in reducing crime outcomes at treatment places relative to control places. The effect was smaller for randomized designs but still statistically significant and positive. When displacement and diffusion effects were measured, a diffusion of crime prevention benefits was associated with hot spots policing.

**Authors' Conclusions:**

The extant evaluation research suggests that hot spots policing is an effective crime prevention strategy. The research also suggests that focusing police efforts on high‐activity crime places does not inevitably lead to crime displacement; rather, crime control benefits may diffuse into the areas immediately surrounding the targeted locations.

## 1 PLAIN LANGUAGE SUMMARY

### 1.1 Hot spots policing is associated with reductions in crime

Hot spots policing is associated with small but meaningful reductions in crime at locations where criminal activities are most concentrated. Focusing police efforts at high activity crime places is more likely to produce a diffusion of crime prevention benefits into areas adjacent to targeted hot spots than crime displacement.

### 1.2 What is this review about?

Crime is concentrated in small places, or “hot spots,” that generate half of all criminal events. Hot spots policing focuses police resources and attention on these high crime places. For the purpose of this review, hot spots programs must have consisted of police‐led crime prevention efforts that targeted high‐activity crime “places” rather than larger areas such as neighborhoods.

This review considers both randomized controlled experimental and quasiexperimental evaluations of the effects of hot spots policing interventions on crime where the control group in each study received routine levels of traditional police enforcement tactics.

**What is the aim of this review?**

This Campbell systematic review assesses the preventive effects of focusing police‐led crime prevention efforts on crime “hot spots” as compared to traditional police crime control strategies. The review summarizes evidence from 65 studies containing 78 tests of hot spots policing interventions, including 27 randomized controlled trials and 38 quasiexperimental evaluations.

### 1.3 What studies are included?

A total of 65 studies containing 78 tests of hot spots policing interventions were identified. However, standardized effects sizes were only calculated for 73 main effects tests due to reporting deficiencies in three included studies.

All studies were published from 1989 to 2017: 51 studies were conducted in the United States, four in the United Kingdom, four in Sweden, and six in other countries.

### 1.4 What are the main findings of this review?

#### 1.4.1 Does focusing crime prevention efforts on crime hot spots reduce crime?

Yes. Hot spots policing generates statistically significant small reductions in overall crime and disorder in areas where the strategy is implemented. These crime control gains were evident across specific categories of crime outcomes including drug offenses, disorder offenses, property crimes, and violent crimes.

#### 1.4.2 Does policing crime hot spots inevitably produce crime displacement effects?

No. Overall, it is more likely that hot spots policing generates crime control benefits that diffuse into the areas immediately surrounding the targeted locations than displacing crime into nearby locations.

### 1.5 What do the findings of this review mean?

Findings from this review support hot spots policing as a proactive crime reduction strategy. Police departments should incorporate focusing resources at high‐activity crime places as part of their broader approach to crime prevention.

The majority of studies included in the updated review have been published since the previous iteration of the review and utilized rigorous research designs.

Despite the drastic increase in eligible studies, only one study conducted a formal cost‐benefit assessment of the hot spot policing intervention. The growth of hot spots policing warrants further empirical attention on the efficiency of hot spots policing for reducing crime.

### 1.6 How up‐to‐date is this review?

The review authors searched for studies up to February 2017.

## 2 BACKGROUND

### 2.1 The issue

Over the past 30 years, crime scholars and practitioners have pointed to the potential benefits of focusing crime prevention efforts on crime places. A number of studies suggest that crime is not spread evenly across city landscapes. Rather, there is significant clustering of crime in small places, or “hot spots,” that generate half of all criminal events (Pierce, Spaar, & Briggs, 1988; Sherman, Gartin, & Buerger, 1989). Even within the most crime‐ridden neighborhoods, crime clusters at a few discrete locations and other areas are relatively crime free (Weisburd, Groff, & Yang, 2012). More recent research has reinforced this idea of crime concentrations (Braga, Andresen, & Lawton, 2017) and led Weisburd (2015) to argue that there is a “law of crime concentration” at places showing not just that crime is concentrated but that it is concentrated at similar levels across cities and across time. A number of researchers have argued that many crime problems can be reduced more efficiently if police officers focused their attention to these persistent high‐activity crime places (Braga & Weisburd, 2010; Sherman & Weisburd, 1995; Weisburd, 1997). The appeal of focusing limited resources on a small number of high‐activity crime places is straightforward. If we can prevent crime at these hot spots, then we might be able to control citywide crime levels (Weisburd, Braga, Groff, & Wooditch, 2017).

Police officers have long recognized the importance of place in crime problems. Police officers know the locations within their beats that tend to be trouble spots and are often very sensitive to signs of potential crimes across the places that comprise their beats. As Bittner (1970, p. 90) suggests in his classic study of police work, some officers know “the shops, stores, warehouses, restaurants, hotels, schools, playgrounds, and other public places in such a way that they can recognize at a glance whether what is going on within them is within the range of normalcy.” The traditional response to such trouble spots typically included heightened levels of patrol and increased opportunistic arrests and investigations. Putting police officers in high crime locations may be an old and well‐established idea; however, in the long history of policing, police crime prevention strategies did not focus systematically on crime hot spots until only very recently (Braga & Schnell, 2018). The availability of powerful crime mapping software packages has allowed police departments to identify and address problem places more easily than was previously possible in the days when pin maps were necessary to examine crime concentrations (Weisburd & Lum, 2005).

### 2.2 Hot spots policing

Hot spots policing has become a very popular way for police departments to prevent crime. Many police departments report having the capability to manage and analyze crime data in sophisticated ways and, through management innovations such as Compstat, hold officers accountable for implementing problem‐solving strategies to control hot spot locations (Weisburd, Mastrofski, McNally, Greenspan, & Willis, 2003). In the words of then‐New York Police Department Deputy Commissioner Jack Maple, “the main principle of deployment can be expressed in one sentence: ‘map the crime and put the cops where the dots are.’ Or, more succinctly: ‘Put cops on dots.’” (Maple, 1999, p. 128). The 2007 Law Enforcement Management and Administrative Statistics survey reported that nearly all police agencies in large metropolitan centers use computers for hot spots identification (Reaves, 2010). The Police Executive Research Forum (2008) surveyed 176 U.S. police departments and found that nearly 9 out of 10 agencies used hot spots policing strategies to deal with violent crime in their jurisdictions and that problem‐solving techniques were often deployed to address violent crime hot spots. In a more recent study of a representative sample of police agencies, the National Police Research Platform reported that 75% of the agencies surveyed used the hot spots policing approach (Mastrofski & Fridell, n.d.; reported in Weisburd & Majmundar 2018).

A growing body of research evidence suggests that focused police interventions, such as directed patrols, proactive arrests, and problem‐oriented policing (POP), can produce significant crime prevention gains at high‐crime “hot spots” (see, e.g., Braga, 2008; Eck, 1997, 2002; Weisburd & Eck, 2004). Indeed, the National Research Council's Committee to Review Research on Police Policy and Practices found that “...studies that focused police resources on crime hot spots provided the strongest collective evidence of police effectiveness that is now available” (Skogan & Frydl, 2004, p. 250). More recently, the National Research Council's Committee on Proactive Policing concluded that the available research evidence suggests that hot spots policing strategies generate statistically significant crime reduction effects (Weisburd & Majmundar, 2018). Critics of place‐based interventions, however, charge that such policing strategies result in displacement—that is, criminals move to places not protected by police intervention (e.g., Blattman, Green, Ortega, & Tobón, 2017; Reppetto, 1976). The available evidence suggests that hot spots policing interventions are more likely to be associated with the diffusion of crime control benefits into surrounding areas rather than crime displacement (e.g., Braga & Weisburd, 2010; Weisburd & Majmundar, 2018; Weisburd et al., 2006).

### 2.3 Theoretical underpinnings

The crime prevention potency of hot spots policing is supported by two key theoretical mechanisms: deterrence and crime opportunity reduction (Braga & Schnell, 2018). Deterrence theory suggests that crime can be prevented when the costs of committing the crime are perceived by the offender to outweigh the benefits (Gibbs, 1975; Zimring & Hawkins, 1973). Much of the literature evaluating deterrence focuses on the effect of changing certainty, swiftness, and severity of punishment associated with certain acts on the prevalence of those crimes (Apel & Nagin, 2011; Nagin, 2013; Paternoster, 1987). Reflecting on the theoretical and policy lessons learned from hot spots policing evaluations, Nagin et al. (2015) argued that increasing police visibility in crime hot spots will generate substantial marginal deterrent effects by heightening potential offenders' perceived risk of apprehension and discouraging them from taking advantage of concentrated crime opportunities in these small places. Indeed, in the well‐known Minneapolis hot spots patrol experiment, Sherman and Weisburd (1995) claimed evidence of place‐specific “micro‐deterrence” associated with increased police presence in hot spot areas (p. 646).

Hot spots policing is also highly influenced by three complementary crime opportunity theories: rational choice, routine activities, and environmental criminology (Braga & Clarke, 2014; Eck & Weisburd, 1995). The rational choice perspective assumes that “offenders seek to benefit themselves by their criminal behavior; that this involves the making of decisions and choices, however rudimentary on occasion these choices may be; and that these processes, constrained as they are by time, the offender's cognitive abilities, and by the availability of relevant information, exhibited limited rather than normative rationality” (Cornish & Clarke, 1987, p. 933). This perspective is often combined with routine activity theory to explain criminal behavior during the crime event (Clarke & Felson, 1993). Routine activities theory posits that a criminal act occurs when a likely offender converges in space and time with a suitable target (e.g., victim or property) in the absence of a capable guardian (Cohen & Felson, 1979). Rational offenders come across criminal opportunities as they go about their daily routines and make decisions whether to commit offenses. The assumption is that, if victims and offenders are prevented from converging in space and time through the effective manipulation of the situations and settings that give rise to criminal opportunities, police can reduce crime.

Environmental criminology explores the distribution and interaction of targets, offenders, and opportunities across time and space; understanding the characteristics of places, such as the presence of crime attractors or crime generators, is important as these attributes give rise to the opportunities that rational offenders will encounter during their routine activities (Brantingham & Brantingham, 1991). Although this perspective is primarily concerned with applied crime prevention, Weisburd et al. (1992, p. 48) suggest “environmental criminology's basic contribution lay in its call for a change in the unit of analysis from persons to places.” The attributes of a place are viewed as key in explaining clusters of criminal events. For example, a poorly lit street corner with an abandoned building, located near a major thoroughfare, provides an ideal location for a drug market. The lack of proper lighting, an abundance of “stash” locations around the derelict property, a steady flow of potential customers on the thoroughfare, and a lack of informal social control (termed defensive ownership) at the place generates an attractive opportunity for drug sellers. In many such cases, the police spend considerable time and effort arresting sellers without noticeably impacting the drug trade. The compelling criminal opportunities at the place attract sellers and buyers, and thus sustain the market. If the police want to be more efficient at disrupting the market, this suggests they should focus on the features of the place which cause the drug dealing to cluster at that particular location (see, e.g., Green, 1996).

### 2.4 Why it is important to do the review

The widespread use of hot spots policing to prevent crime warrants ongoing careful reviews of the available empirical evidence on the crime control benefits of the approach. If hot spots policing program are effective in controlling crime, the societal benefits may be considerable. For instance, in an influential article, Durlauf and Nagin (2011) suggested that crime and incarceration in the United States would both be reduced if resources were shifted from imprisonment to policing. Among other focused police interventions, they specifically point to evaluations of hot spots policing deployment strategies as evidence that the police, when properly oriented, can prevent crime.

As new program evaluations are completed, however, conclusions on the crime control efficacy of hot spots policing could change in response to the growing scientific evidence base. For instance, several recent hot spots policing studies have reported null effects (Gerell, 2016), crime increases (Phillips, Wheeler, & Kim, 2016), and modest crime displacement (Blattman et al., 2017). This document provides an updated version of a previously completed Campbell Collaboration systematic review of the effects of hot spots policing on crime (Braga, 2001, 2005, 2007; Braga, Papachristos, & Hureau, 2012, 2014).

## 3 OBJECTIVES

This review will synthesize the existing published and nonpublished empirical evidence on the effects of focused police crime prevention interventions at high‐activity crime places and will provide a systematic assessment of the preventive value of focused police crime prevention efforts at crime hot spots. The review also examined whether focused police actions at specific locations result in crime displacement or a diffusion of crime control benefits.

## 4 METHODS

This review synthesizes existing published and nonpublished empirical evidence on the effects of focused police crime prevention interventions at crime hot spots and provides a systematic assessment of the preventive value of these programs. In keeping with the conventions established by the systematic reviews methods literature, the stages of this review and the criteria used to select eligible studies are described below.

### 4.1 Criteria for considering studies for this review

#### 4.1.1 Types of studies

In eligible studies, crime places that received the hot spots policing intervention were compared to places that experienced routine levels of traditional police service (i.e., regular levels of patrol, ad‐hoc investigations, etc.). The comparison group in each study had to be either experimental or quasiexperimental (nonrandomized) (Campbell & Stanley, 1966; Cook & Campbell, 1979; Shadish, Cook, & Campbell, 2002).

#### 4.1.2 Types of areas

The units of analysis were crime hot spots or high‐activity crime “places.” As Eck (1997, p. 7‐1) suggests, “a place is a very small area reserved for a narrow range of functions, often controlled by a single owner, and separated from the surrounding area… examples of places include stores, homes, apartment buildings, street corners, subway stations, and airports.” All studies using units of analysis smaller than a neighborhood or community were considered. This constraint was placed on the review process to ensure that identified studies were evaluating police strategies focused on the small number of locations that generate a disproportionate amount of crime in urban areas.

As described earlier, hot spots policing was a natural outgrowth of theoretical perspectives that suggested specific places where crime concentrates were an important focus for strategic crime prevention efforts. Police interventions implemented at the community or neighborhood level would not be specifically focused on small places, often encompassing only one or a few city blocks, that would be considered hot spots of crime. However, this review does include quasiexperimental designs that compare changes at larger areal units, such as policing districts or census tracts, if the implemented hot spots policing program was clearly focused at specific places within the larger areal unit. For instance, The Kansas City Gun Project quasiexperiment evaluated the effects of increased gun seizures focused at gun hot spots within an 8 by 10 block police beat on gun crime relative to traditional policing services in comparison police beats (Sherman & Rogan, 1995a).

#### 4.1.3 Types of interventions

To be eligible for this review, interventions used to control crime hot spots were limited to police‐led crime control efforts. Eligible police interventions included traditional tactics such as directed patrol and heightened levels of traffic enforcement as well as alternative strategies such as aggressive disorder enforcement and POP (Goldstein, 1990). Studies of police crackdown programs were also considered (see, e.g., Sherman, 1990). However, to be included in the review, crackdown programs had to be focused on very specific places. Some ongoing attention to crime hot spots must be a characteristic of the program whether it was a series of subsequent crackdowns or simple maintenance of the targeted area through other means (e.g., additional follow‐up directed patrol). This inclusion criterion ensured that only crackdown programs that were similar to more formal hot spots policing programs were considered.

#### 4.1.4 Types of outcome measures

Eligible studies had to measure the effects of police intervention on officially recorded levels of crime at places such as crime incident reports, citizen emergency calls for service, and arrest data. Other outcomes measures such as survey, interview, systematic observations of social disorder (such as loitering, public drinking, and the solicitation of prostitution), systematic observations of physical disorder (such as trash, broken windows, graffiti, abandoned homes, and vacant lots), and victimization measures used by eligible studies to measure program effectiveness were also coded and analyzed. We closely examined any eligible studies that reported outcome data on community reactions to implemented hot spots policing programs.

Particular attention was paid to studies that measured crime displacement effects and diffusion of crime control benefit effects. As mentioned earlier, policing strategies focused on specific locations have been criticized as resulting in displacement (see Reppetto, 1976). More recently, academics have observed that crime prevention programs may result in the complete opposite of displacement—that crime control benefits were greater than expected and “spill over” into places beyond the target areas (Clarke & Weisburd, 1994; Weisburd et al., 2006). The quality of the methodologies used to measure displacement and diffusion effects, as well as the types of displacement (spatial, temporal, target, modus operandi) examined, was assessed. Based on our a priori knowledge of several hot spots policing experiments (e.g., Braga & Bond, 2008; Weisburd & Green, 1995a), we expected most analyses of displacement and diffusion effects to compare pre‐ and posttest counts of official crime data in catchment areas surrounding treatment and control hot spots.

### 4.2 Search strategies for identification of studies

Several strategies were used to perform an exhaustive search for literature fitting the eligibility criteria. First, a keyword search was performed on an array of online abstract databases (see lists of keywords and databases below). Second, the bibliographies of past narrative and empirical reviews of literature that examined the effectiveness of police crime control programs were reviewed (Braga, 2008, 2016; Higginson & Mazerolle, 2014; Johnson, Guerette, & Bowers, 2014; Lum, Koper, & Telep, 2011; Telep & Weisburd, 2012; Telep, Weisburd, Gill, Vitter, & Teichman, 2014; Weisburd & Telep, 2014; Weisburd, Farrington, & Gill, 2017; Weisburd, Telep, & Braga, 2015;). Third, forward searches for works that cited seminal hot spots policing studies were performed (Braga & Bond, 2008; Braga et al., 1999, 2014; Sherman & Rogan, 1995a; Sherman & Weisburd, 1995; Sherman, Buerger, & Gartin, 1989; Weisburd & Green, 1995a; Weisburd et al., 2006). Fourth, bibliographies of past completed Campbell systematic reviews of police crime prevention efforts were searched (Bowers, Johnson, Guerette, Summers, & Poynton, 2011; Braga & Weisburd, 2012; Braga, Welsh, & Schnell, 2015; Koper & Mayo‐Wilson, 2012; Mazerolle, Bennett, Davis, Sargeant, & Manning, 2013). Fifth, hand searches of leading journals in the field were performed.^1^

The searches were all completed between January 2017 and February 2017. Thus, the review only covers studies published in 2017 and earlier. Sixth, after finishing the above searches and reviewing the studies as described later, the list of studies meeting our eligibility criteria was emailed in June 2017 to leading criminology and criminal justice scholars knowledgeable in the area of hot spots policing strategies. These 146 scholars were defined as those who authored at least one study which appeared on our inclusion list, anyone involved with the National Academy of Sciences review of police research and other leading scholars (see Appendix A). This helped to identify studies the above searches left out as these experts were able to make referrals to studies that were missed, particularly unpublished studies. Finally, an information specialist was engaged at the outset of our review and at points along the way in order to ensure that appropriate search strategies were used to identify the studies meeting the criteria of this review.^2^

The following 15 databases were searched:

1. Criminal Justice Abstracts
2. Sociological Abstracts
3. National Criminal Justice Reference Service Abstracts
4. Educational Resources Information Clearinghouse
5. Google Scholar
6. Proquest Dissertation and Theses A&I
7. Westlaw Next
8. Government Publications Office, Monthly Catalog (GPO Monthly)
9. Informit
10. Web of Science Core Collection
11. Academic Search Premier
12. HeinOnline
13. Social Sciences Premium Collection
14. Rutgers University Gottfredson Library gray literature database
15. C2 SPECTR (Campbell Collaboration Social, Psychological, Educational and Criminological Trials Register)^3^

The following terms were used to search the 15 databases listed above:

(a) Hot spot AND police
(b) Crime place AND police
(c) Crime clusters AND police
(d) Crime displacement
(e) Place‐oriented interventions
(f) High crime areas AND police
(g) High crime locations AND police
(h) Targeted policing
(i) Directed patrol
(j) Crackdowns
(k) Enforcement swamping

### 4.3 Data collection and analysis

#### 4.3.1 Details of study coding categories

All eligible studies were coded (see coding protocol attached in Appendix B) on a variety of criteria including:

(a) Reference information (title, authors, publication etc.)
(b) Nature of description of selection of site, problems and so forth.
(c) Nature and description of selection of comparison group or period
(d) The unit of analysis
(e) The sample size
(f) Methodological type (randomized experiment or quasiexperiment)
(g) A description of the hot spots policing intervention
(h) Dosage intensity and type
(i) Implementation difficulties
(j) The statistical test(s) used
(k) Reports of statistical significance (if any)
(l) Effect size/power (if any)
(m) The conclusions drawn by the authors

The four authors independently coded each eligible study. Where there were discrepancies, the authors jointly reviewed the study and determined the final coding decision.

#### 4.3.2 Statistical procedures and conventions

Analysis of outcome measures across studies were carried out in a uniform manner and, when appropriate and possible, involved quantitative analytical methods. We used meta‐analyses of program effects to determine the size and direction of the effects and to weight effect sizes based on the variance of the effect size and the study sample size (Lipsey & Wilson, 2001). In this systematic review, the standardized mean difference effect size (also known as Cohen's *d*; see Rosenthal, 1994) was used. Computation of effect sizes in the studies was not always direct. The goal was to convert all observed effects into a standardized mean difference effect size metric. Indeed, it was sometimes difficult to develop precise effect size metrics from published materials. This reflects a more general problem in crime and justice with “reporting validity” (Farrington, 2006; Lösel & Köferl, 1989) and has been documented in reviews of reporting validity in crime and justice studies (see Perry & Johnson, 2008; Perry, Weisburd, & Hewitt, 2010).

The Effect Size Calculator, developed by David B. Wilson and available on the Campbell Collaboration's web site, was used to calculate standardized mean difference effect sizes for reported outcomes in each study.^4^ Biostat's Comprehensive Meta Analysis Version 2.2 was then used to conduct the meta‐analysis of effect sizes. For many of the included studies, treatment and control group crime counts were used to calculate effect sizes. From these raw counts, Odds ratios (ORs) were first calculated. To obtain Cohen's *d*, the log of this OR was then multiplied by √3/π (Hasselblad & Hedges, 1995). The variance of log OR was calculated as the sum of the reciprocal terms in the cells immediately below. The computational formulae are presented here:

$$\begin{array}{lll} \; & \text{Pre} & \text{Post}\\ \text{Treatment} & a & b\\ \text{Control} & c & d \end{array}$$

$$\begin{array}{l} \text{OR}\;=\;(b\;⁎\;c)/(a\;⁎\;d),\\ V(\text{LOR})=(1/a)+(1/b)+(1/c)+(1/d). \end{array}$$

An adjustment for over‐dispersion was then made using the method in Farrington, Gill, Waples, and Argomaniz (2007): the adjusted V(LOR) is computed as the product of V(LOR) and *D*, with *D* = 0.0008 × *N* + 1.2. *N* is indexed as the mean number of incidents per case and is calculated as the total number of incidents (*a* + *b* +* c* + *d*) divided by the total number of treatment plus control cases. This adjusted V(LOR) is then multiplied by (3/π2) to give the final variance of the effect size [V(d)] (Hasselblad & Hedges, 1995).

In certain included studies, counts were not provided or could not be reconstructed from information in the study report. We then attempted to contact study authors to gain access to the original data and/or request further output that would allow us to calculate Cohen's *d*. When this was not possible, we attempted to use other methods. For example, many recent papers reported incidence rate ratios (IRRs) in order to estimate treatment effects conditional on the use of covariates. In such cases, ORs were obtained by taking the product of the IRR and a ratio of the pretest means in the control and treatment group [OR = IRR × (mean_pre_C/ mean pre_T)]. This allows *d* to be calculated from log OR using standard methods. The standard error of this IRR is squared to obtain the variance. In other included studies, Cohen's *d* could not be estimated in either way described above, and other methods were pursued. For instance, in Weisburd and Green (1995b), the *p* levels from a mixed‐model analysis of variance were used to compute the effect sizes. The *p* level for each contrast was first converted to a *Z* score which was then used to calculate a correlational effect size (*r*). Using conventional formulae, this effect size was then converted to Cohen's *d*.

#### 4.3.3 Determination of independent findings

One problem in conducting meta‐analyses in crime and justice is that investigators often do not prioritize outcomes examined. This is common in studies in the social sciences in which authors consider it good practice to report all relevant outcomes. For example, the Jersey City Drug Market Analysis Program experiment presents an array of outcome measures including violence, property, disorder, and narcotics calls for service (Weisburd & Green, 1995a). However, the lack of prioritization of outcomes in a study raises the question of how to derive an overall effect of treatment. Specifically, the reporting of one significant result may reflect a type of “creaming” in which the authors focus on one significant finding while ignoring the less positive results of other outcomes. But authors commonly view the presentation of multiple findings as a method for identifying the specific contexts in which the treatment is effective. When the number of such comparisons is small and therefore unlikely to affect the error rates for specific comparisons, such an approach is often valid.

All studies for which a standardized effect size could be obtained were analyzed using three approaches. The first approach is conservative; we calculated an overall mean effect size for each study that combined all reported outcomes. The second represents the largest effect reported in the studies and offers an upper bound to the review findings. It is important to note that in some of the studies with more than one outcome reported, the largest outcome reflected what authors thought would be the most direct program effect. This was true for the Jersey City Drug Market Analysis Program experiment, which examined a wider range of crime outcome measures, but suggested that the largest program effects would be found in the case of disorder calls of service given the program's focus on street‐level drug markets (Weisburd & Green, 1995a). Finally, the smallest effect size for each study was analyzed. This approach is the most conservative and likely underestimates the effect of hot spots policing programs on crime. It was used here primarily to provide a lower bound to the review findings.

#### 4.3.4 Treatment of qualitative research

Qualitative research on crime and disorder outcomes was not included in this systematic review. The authors hope that a qualitative researcher will assist in future updates to this review with a synthesis of qualitative evaluation measures.

## 5 RESULTS

### 5.1 Selection of studies

#### 5.1.1 Results of the search

Search strategies in the systematic review process generate a large number of citations and abstracts for potentially relevant studies that must be closely screened to determine whether the studies meet the eligibility criteria (Farrington & Petrosino, 2001). The screening process yields a much smaller pool of eligible studies for inclusion in the review. Search strategies used for this review yielded a total of 26,038 titles, citations, and abstracts. Naturally, due to the number of databases, key terms, and tactics used, there was an inevitable overlap in search results.^5^ Each result was reviewed for any suggestion of an experimental or quasiexperimental evaluation of hot spots policing interventions. Two hundred and seventy‐four distinct abstracts were selected for closer review and the full‐text reports, journal articles, and books for these abstracts were acquired and carefully assessed to determine whether the interventions and evaluations met the eligibility criteria.

**Figure 1:**
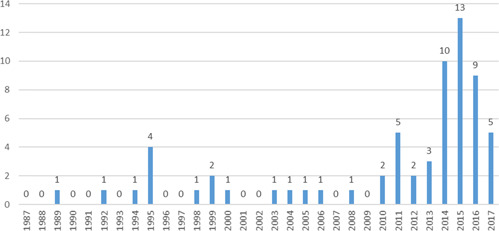
Number of eligible hot spots policing studies by year (*N *= 65) [Color figure can be viewed at wileyonlinelibrary.com]

The original Campbell systematic review of the effects of hot spots policing on crime identified nine studies (Braga, 2001) and first update of the review included 19 studies (Braga, Papachristos, and Hureau, 2014). In this iteration, we identified 65 eligible studies to be included in the updated systematic review and meta‐analysis. Figure 1 presents the yearly counts of included hot spots policing evaluations and highlights the strong growth in hot spots policing studies since the completion of the previous review. Indeed, we identified 46 new studies representing a 242% increase in eligible studies since the prior review. The 65 eligible studies included:

1. Minneapolis Repeat Call Address Policing (RECAP) Program (Sherman et al., 1989)
2. New York Tactical Narcotics Teams (Sviridoff, Sadd, Curtis, & Grinc, 1992)
3. St. Louis Problem‐Oriented Policing in three Drug Market Locations Study (Hope, 1994)
4. Minneapolis Hot Spots Patrol Program (Sherman & Weisburd, 1995)
5. Jersey City Drug Markets Analysis Program (DMAP) (Weisburd & Green, 1995a)
6. Kansas City Gun Project (Sherman & Rogan, 1995a)
7. Kansas City Crack House Police Raids Program (Sherman & Rogan, 1995b)
8. Beenleigh Calls for Service Project (Criminal Justice Commission, 1998)
9. Jersey City Problem‐Oriented Policing at Violent Places Project (Braga et al., 1999)
10. Houston Targeted Beat Program (Caeti, 1999)
11. Oakland Beat Health Program (Mazerolle, Price, & Roehl, 2000)
12. Pittsburgh Police Raids at Nuisance Bars Program (Cohen, Gorr, & Singh, 2003)
13. Buenos Aires Police Presence after Terror Attack Study (DiTella & Schargrodsky 2004)
14. Philadelphia Drug Corners Crackdowns Program (Lawton, Taylor, & Luongo, 2005)
15. Jersey City Displacement and Diffusion Study (Weisburd et al., 2006)
16. Lowell Policing Crime and Disorder Hot Spots Project (Braga & Bond, 2008)
17. Jacksonville Policing Violent Crime Hot Spots Project (Taylor, Koper, & Woods, 2011)
18. Philadelphia Foot Patrol Program (Ratcliffe, Taniguchi, Groff, & Wood, 2011)
19. Boston Safe Streets Teams Program (Braga, Hureau, & Papachristos, 2011)
20. DDACTS Program in Washoe County (Beck, 2010)
21. Safer Cities Initiative in Los Angeles (Berk & MacDonald, 2010)
22. License Plate Reader Patrols in Crime Hot Spots in two Adjacent Jurisdictions (Lum, Hibdon, Cave, Koper, & Merola, 2011)
23. Camden 28‐Day Crime Suppression Initiative (Ratcliffe & Breen, 2011)
24. Predictive Risk Mapping and Policing in Trafford, Greater Manchester (Fielding & Jones, 2012)
25. Broken Windows Style Crackdowns in three California Cities (Weisburd, Hinkle, Famega, & Ready, 2012)
26. Operation LASER in Los Angeles (Uchida & Swatt, 2013)
27. Palos Verdes Team Policing Project (Martinez, 2013)
28. License Plate Readers at Crime Hot Spots Experiment in Mesa, Arizona (Koper, Taylor, & Woods, 2013)
29. Lowell Smart Policing Initiative (Bond, Hajjar, Ryan, & White, 2014)
30. DDACTS Program in Shawnee, Kansas (Bryant, Collins, & Villa, 2014)
31. Summer Crime Initiative in Washington, DC (Mazeika, 2014)
32. Operation Impact in Newark, New Jersey (Piza & O'Hara, 2014)
33. St. Louis Metropolitan PD's Firearms Violence Hot Spots Policing Experiment (Rosenfeld, Deckard, & Blackburn, 2014)
34. Hot Spots Randomized Field Trial in Sacramento, California (Telep, Mitchell, & Weisburd, 2014)
35. Trinidad & Tobago Police Services Hotspot Experiment (Sherman et al., 2014)
36. Policing Crime Hot Spots in Stockholm, Sweden (Marklund & Merenius, 2014)
37. Policing Crime Hot Spots in Eskilstuna, Sweden (Marklund & Merenius, 2014)
38. Anti‐Drunk Driving Program in Rajasthan, India (Banerjee, Duflo, Keniston, & Singh, 2014)
39. Philadelphia Policing Tactics Experiment (Groff et al., 2015)
40. Colorado Springs PD's Risk‐Based Intervention (Kennedy, Caplan, & Piza, 2015)
41. Newark PD's Risk‐Based Intervention (Kennedy et al., 2015)
42. Kansas City PD's Risk‐Based Intervention (Kennedy et al., 2015)
43. Glendale PD's Risk‐Based Intervention (Kennedy et al., 2015)
44. St. Louis County Hot Spots in Residential Areas Study (Kochel, Burruss, & Weisburd, 2015)
45. Mobile Computing Technology at Crime Hot Spots in a Suburban County (Koper, Lum, & Hibdon, 2015)
46. Proactive CCTV Monitoring with Directed Police Patrol in Newark, New Jersey (Piza, Caplan, Kennedy, & Gilchrist, 2015)
47. Tactical Police Response at Micro‐Time Hot Spots (Santos & Santos 2015a, 2015b)
48. Philadelphia GunStat Model (Sorg, 2015)
49. Dallas Patrol Management Experiment (Weisburd et al., 2015)
50. West Midlands Police's Randomized Control Trial of Policing Hot Spots (Williams, 2015)
51. Actively Monitored CCTVs in Stockholm, Sweden (Marklund & Holmberg, 2015)
52. Operation Style in Peterborough, England (Ariel, Weinborn, & Sherman, 2016)
53. Glendale Smart Policing Initiative (Dario, 2016)
54. Policing Violent Crime Hot Spots in Malmö, Sweden (Gerell 2016)
55. Operation Impact in New York City (MacDonald, Fagan, & Geller, 2016)
56. Kansas City Foot Patrol Project (Novak, Fox, Carr, & Spade, 2016)
57. Police Paramilitary Raids in Buffalo, New York (Phillips et al., 2016)
58. Offender‐Focused Police Intervention at Hot Spots (Santos & Santos, 2016)
59. New Haven Smart Policing Initiative (Sedelmaier & Hipple, 2016)
60. Operation Menas in London, England (Ariel and Partridge 2016)
61. Investigating Hot Spots Policing in Copenhagen, Denmark (Attermann, 2017)
62. Hot Spots Policing in Bogotá, Colombia (Blattman et al., 2017)
63. Philadelphia Predictive Policing Experiment (Ratcliffe et al., 2017)
64. Flint DDACTS Program (Rydberg, McGarrell, Norris, & Circo, 2017)
65. Operation Strikeforce in Buffalo, New York (Wheeler & Phillips, 2018)

There were a number of studies identified during the abstract search that were worthy of further consideration but ultimately determined not to meet the inclusion criteria. These studies are noted in Appendix C.

### 5.2 Characteristics of selected studies

Table 1 presents the basic characteristics of the 65 eligible hot spots policing studies. Fifty‐one of the 65 (78.5%) identified studies were conducted in the United States. Four hot spots policing evaluations were conducted in the United Kingdom and four eligible studies were completed in Sweden. One hot spots policing evaluation was conducted in each of the following countries: Argentina, Australia, Colombia, Denmark, India, and Trinidad and Tobago. Twenty‐seven studies (41.5%) were completed in medium‐sized cities with between 200,000 and 500,000 residents, 25 studies (38.5%) were completed in large cities with more than 500,000 residents, and 12 studies were completed in smaller cities with <200,000 residents (18.5%). One study included both a large and small city in the designated study area (1.5%): Lum et al. (2011) evaluated the impact of license plate reader technology in crime hot spots in two adjacent jurisdictions located in Alexandria City and the eastern portion of Fairfax County (VA). Eleven cities were the research sites for multiple hot spots policing evaluations. These cities were Philadelphia (five studies), Kansas City (four studies), Jersey City (three studies), Newark (three studies), St. Louis (three studies), Los Angeles (two studies), Lowell (two studies), Minneapolis (two studies), New York City (two studies), Port St. Lucie (two studies), and Stockholm (two studies). Thirty‐six of the eligible hot spots policing studies were published in peer‐reviewed journals (55.4%), 16 were available as published reports (24.6%), seven were available as unpublished theses/dissertations (10.8%), and six were available as unpublished reports or working papers (9.2%).

**Table 1:** Characterisics of eligible hot spots policing evaluations

Characteristic	*N*	%
Evaluation country (*N* = 65)		
United States	51	78.5
United Kingdom	4	6.2
Sweden	4	6.2
Other^a^	6	9.2
City population (*N* = 65)		
Small (<200,000 residents)	12	18.5
Medium (200,000–500,000 residents)	27	41.5
Large (>500,000 residents)	25	38.5
Small and large	1	1.5
Publication type (*N* = 65)		
Peer‐reviewed article	36	55.4
Published report	16	24.6
Thesis/dissertation	7	10.8
Unpublished report/working paper	6	9.2
Evaluation type (*N* = 65)		
Randomized controlled trial	27	41.5
Quasiexperimental	38	58.5
Intervention type (*N* = 78)		
Problem‐oriented policing	27	34.6
Increased policing	51	65.4
Displacement/diffusion effects (*N* = 78)		
Measured displacement/diffusion	46	59.0
Did not measure displacement/diffusion	32	41.0

^a^ Argentina, Australia, Colombia, Denmark, India, and Trinidad and Tobago.

Twenty‐seven eligible studies used randomized controlled trials (41.5%) and 38 eligible studies used quasiexperimental research designs (58.5%) to evaluate the effects of hot spots policing on crime. Eleven of the 65 eligible studies (16.9%) evaluated more than one hot spots policing intervention. Nine studies examined two separate hot spots policing interventions and two studies examined three hot spots policing interventions. For instance, the seminal Minneapolis RECAP experiment separately evaluated POP interventions at residential and commercial addresses (Sherman et al., 1989). More recently, Blattman et al. (2017) evaluated the impacts of increased police patrol and, separately, increased police patrol plus municipal services on high‐crime street segments in Bogotá, Colombia. In total, the 65 studies included in this review yielded 78 experimental and quasiexperimental tests of hot spots policing on crime.

Across the 78 tests of hot spots policing, the specific types of hot spots policing interventions fit broadly into two categories: POP and increased traditional policing. More than one‐third of hot spots policing programs focused primarily on reducing crime opportunities at places by engaging strategies consistent with POP (*N* = 27, 34.6%). In these initiatives, the POP strategies generally attempted to change the underlying conditions and situational dynamics that caused problems to recur in high‐activity crime places (Braga, 2008; Goldstein, 1990). Increased traditional policing was used in two‐thirds of the eligible hot spots policing (*N* = 51, 65.4%). These programs were generally designed to deter offenders from committing crimes in hot spot areas by increasing police presence and enforcement activities. This was most commonly attempted through increased foot or vehicle patrol (*N* = 31), drug enforcement operations (*N* = 6), offender‐focused apprehension programs (*N* = 4), actively monitored CCTV with directed patrol (*N* = 3), and other kinds of increased enforcement activities (*N *= 7).^6^ Crime displacement and diffusion of crime control benefits effects were assessed for 46 of the 78 tests of hot spots policing (58.9%).

A noteworthy majority of the hot spots policing evaluations concluded that hot spots policing programs generated significant crime control benefits in the treatment areas relative to the control areas. Only 16 of the 78 tests (20.5%) of hot spots policing interventions did not report noteworthy crime control gains associated with the approach. Table 2 summarizes the treatments, hot spot definitions, and research designs. Table 3 summarizes the main effects of the intervention on crime and disorder measures, treatment effects as measured by other nonofficial data sources, and, if measured, the immediate spatial displacement and diffusion of crime control benefits effects. A more detailed narrative review of the 65 hot spots policing studies and the 78 tests contained in the eligible studies is provided in Appendix D.

**Table 2:** Hot spots policing experiments and quasiexperiments

Study	Treatment	Hot spot definition	Research design
Minneapolis (MN) RECAP	Problem‐oriented policing interventions comprised of mostly traditional enforcement tactics with some situational responses	Addresses ranked by frequency of citizen calls for service divided into commercial and residential lists; the top 250 commercial and top 250 residential addresses were included in experiment	Randomized controlled trial; control and treatment groups were each randomly allocated 125 commercial and 125 residential addresses
Sherman et al. (1989)	1‐year intervention period		
	Integrity of treatment threatened by large caseloads that outstripped the resources the RECAP unit could bring to bear		
New York (NY) Tactical Narcotics Teams	Undercover and plainclothes police crackdown on street drug markets primarily using “buy and bust” operations	TNT operating in 67th and 70th precincts were evaluated	Quasiexperiment; targeted areas in 67th and 70th precincts were compared to similar areas in 71st precinct
Sviridoff et al. (1992)	90‐day intervention period	Enforcement actions targeted at hot spots in precincts described as particular streets, intersections, and sets of buildings	ARIMA time‐series analyses of assault, robbery, and burglary crime incident trends in treatment and comparison areas
	Treatment in 67th precinct was limited by diminished manpower resources that resulted in fewer arrests and a shortened uniformed patrol maintenance presence		36‐month study time period that compared 3‐month intervention periods to nonintervention months
St. Louis (MO) POP in 3 Drug Areas	Problem‐oriented policing interventions comprised of mostly traditional enforcement tactics with some situational responses	Subjective selection of POP efforts made at three hot spot locations comprised of specific addresses associated with street‐level drug sales	Quasiexperiment; changes in citizen calls at hot spot addresses location were compared to changes in calls at other addresses on the block as well as other blocks in surrounding areas
Hope (1994)	9‐month intervention period		Simple trend analyses including 12 months preintervention and 6 months postintervention period
	No threats to the integrity of the treatment reported		
Minneapolis (MN) Hot Spots	Uniformed police patrol; experimental group, on average, experienced twice as much patrol presence	110 hot spots comprised of address clusters that experienced high volumes of citizen calls for service, had stable numbers of calls for over 2 years, and were visually proximate	Randomized controlled trial; control and treatment groups were each randomly allocated 55 hot spots within statistical blocks
Sherman and Weisburd (1995)	1‐year intervention period		Differences of differences between citizen calls in baseline and experimental years, comparing control and treatment groups
	Breakdown in the treatment noted during the summer months		
Jersey City (NJ) DMAP	Problem‐oriented crackdowns followed by preventive patrol to maintain crime control gains	56 drug hot spot areas identified based on ranking intersection areas with high levels of drug‐related calls and narcotics arrests, types of drugs sold, police perceptions of drug areas, and offender movement patterns	Randomized controlled trial; control and treatment groups were each randomly allocated 28 drug hot spots within statistical blocks
Weisburd and Green (1995b)	15‐month intervention period		Differences of differences between citizen calls during 7‐month pre‐ and posttest periods, comparing control and treatment groups
	Slow progress at treatment places caused intervention time period to be extended by 3 months		
Kansas City (MO) Gun Project	Intensive enforcement of laws against illegally carrying concealed firearms via safety frisks during traffic stops, plain view, and searches incident to arrest on other charges	8 by 10 block target beat selected by federal officials for Weed and Seed grant	Quasiexperiment; target beat matched to a control beat with nearly identical levels of drive‐by shootings
Sherman and Rogan (1995a)	29‐week intervention period	Enforcement actions targeted at hot spots in beat identified by computer analyses	Difference of means comparing weekly gun crimes between intervention period and 29‐week pretest period
			Time‐series analyses of weekly gun crimes for 52 weeks before‐after period (ARIMA—effect of abrupt intervention in time series)
	No threats to the integrity of the treatment reported; Two phases of patrols reported due to shifts in grant funding		Analysis of variance models with one extra pre‐ and post‐year to examine changes in homicides and drive‐by shootings for both patrol phases
Kansas City (MO) Crack House Raids	Court authorized raids on crack houses conducted by uniformed police officers	207 blocks with at least five calls for service in the 30 days preceding an undercover drug buy; sample was restricted to raids on the inside of residences where a drug buy was made that was eligible for a search warrant	Randomized controlled trial; Raids were randomly allocated to 104 blocks and were conducted at 98 of those sites; the other 103 blocks did not receive raids
Sherman and Rogan (1995b)	Intervention period was the day of the raid		
	All but seven cases received randomly assigned treatment as assigned		Differences of differences analytic design; prepost time periods were 30 days before and after raid for experimental blocks, and 30 days before and after controlled buy at treatment block for control blocks
	No threats to the integrity of the treatment reported		
Beenleigh (AUS) Calls for Service Project	Problem‐oriented policing interventions comprised of mostly traditional enforcement tactics with some situational responses 6‐month intervention period	Two groups of 10 addresses that experienced the highest volume of calls during separate 6 month periods	Quasiexperiment; Beenleigh, a lower‐income suburb with a population of 40,000, was matched to similar Browns Plains suburb
Criminal Justice Commission (1998)	No threats to the integrity of the treatment reported		Simple time‐series analyses of total monthly calls for service in 5‐month pretest, 6‐month intervention, and 3‐month posttest periods
			19 pre/post no control case studies
Jersey City (NJ) POP at Violent Places	Problem‐oriented policing interventions comprised of mostly aggressive disorder enforcement tactics with some situational responses	24 violent crime places identified based on ranking intersection areas with high levels of assault and robbery calls and incidents, and police and researcher perceptions of violent areas	Randomized controlled trial; 24 places were matched into like pairs based on simple quantitative and qualitative analyses; control and treatment groups were each randomly allocated 12 places within matched pairs
Braga et al. (1999)	16‐month intervention period		
	Initial slow progress at places caused by resistance of officers to implement intervention		Differences of differences between a number of indicators during 6‐month pre‐ and posttest periods, comparing control and treatment groups
Houston (TX) Targeted Beat Program	Patrol initiative designed to reduce Index crimes in seven beats	Seven highest crime beats were selected for this program	Quasiexperiment; target beats were matched to noncontiguous comparison beats through cluster analysis and correlations of Census data
Caeti (1999)	Three beats used “high visibility patrol” at hot spots	Enforcement actions targeted at hot spots in beats identified by computer analyses	Difference of means in reported crime was used to evaluate program effects for 3‐year preintervention and 2‐year intervention period
	Three beats used “zero tolerance” policing at hot spots		
	One beat used a problem‐oriented policing approach comprised of mostly traditional tactics to control hot spots		
	2‐year intervention period		
	Three “high visibility” patrol beats managed by one substation experienced police resistance to the program		
Oakland (CA) Beat Health Program	Problem‐oriented policing intervention that used civil remedies to alleviate drug and disorder problems at targeted properties	100 street blocks with a place on the block that was referred to the Beat Health Team as having a drug and/or blight problem	Randomized controlled trial; control and treatment groups were each randomly allocated 50 street blocks within residential and commercial statistical blocks
Mazerolle et al. (2000)	5.5‐month intervention period		
	No threats to the integrity of the treatment reported		Differences of differences analytic design; pre‐post time periods were 21.5 months before and 12 months after 5.5‐month intervention period
Pittsburgh (PA) Police Raids at Nuisance Bars	Raids by narcotics squad on nuisance bars to reduce drug selling in and around targeted bar	37 nuisance bar areas and 40 comparison nonnuisance bar areas were included in the analysis	Quasiexperiment; treatment nuisance bars were compared with nonequivalent nonnuisance bars located in the same neighborhood
Cohen et al. (2003)	Intervention period ranged from 1 to 5‐months per nuisance bar area with a mean of 3.7 raids per month during enforcement period	Bar areas were defined as by a 660 foot radius around the treatment and comparison bars that captured roughly two to three blocks in any direction from the bar	OLS and Tobit regression models estimated the impact of the intervention at treatment areas relative to comparison areas controlling for land‐use and population‐based risks
	No threats to the integrity of the treatment reported		36 month study time period with varying pre‐ and posttest periods for targeted bar areas
Buenos Aires (ARG) Police Presence after Terrorist Attack	Increased police presence at Jewish centers in three neighborhoods	37 street blocks with Jewish centers were evaluated	Quasiexperiment; 37 police‐protected blocks were compared with 839 other blocks
DiTella and Schargrodsky 2004	5‐month intervention period		Differences of differences analytic design; pre‐post time periods were 4 months before and 5 months after police protection was implemented
	No threats to the integrity of the treatment reported		
Philadelphia (PA) Drug Corners Crackdowns	Police crackdown that stationed officers at high‐activity drug locations	0.1 mile (~1 street block) areas were constructed around 214 targeted high‐activity drug locations and 73 comparison sites	Quasiexperiment; targeted areas were matched to comparison areas based on spatial analyses of drug crimes and simple analyses of U.S. census data
	18‐week intervention period		
Lawton et al. (2005)	No threats to the integrity of the treatment reported		ARIMA time‐series analyses of drug crime incident and violent crime incident trends in treatment and comparison areas
			139‐week study time period that compared 121 weeks of pretreatment trends to 18 weeks of treatment trends
Jersey City (NJ) Displacement and Diffusion Study	Problem‐oriented policing interventions comprised of mostly traditional enforcement tactics with some situational responses	Two hot spots (one drug and one prostitution) identified based on computerized mapping and database technology supplemented by police officer observations	Quasiexperiment; observed prostitution and drug event trends were examined over a 9‐month period and adjusted for citywide disorder and drug call trends, respectively
Weisburd et al. (2006)	6‐month intervention period		Difference of means tests compared pre‐ and posttest mean observed events
	Burglary hot spot dropped from study due to inadequate dosage of police intervention		
Lowell Policing Crime and Disorder Hot Spots Project	Problem‐oriented policing interventions comprised of mostly aggressive disorder enforcement tactics with some situational responses	34 crime and disorder hot spots identified based on spatial analyses of calls for service and supplemented by police officer and researcher observations	Randomized controlled trial; 24 places were matched into like pairs based on simple quantitative and qualitative analyses; control and treatment groups were each randomly allocated 12 places within matched pairs
Braga and Bond (2008)	12‐month intervention period		
	No threats to the integrity of the treatment reported		Differences of differences between a number of indicators during 6‐month pre‐ and posttest periods, comparing control and treatment groups
Jacksonville (FL) Policing Violent Crime Hot Spots Program Taylor et al. (2011)	Two interventions tested: problem‐oriented policing and direct‐saturation patrol	83 violent crime hot spots identified based on spatial analyses of incidents and calls for service	Randomized controlled trial; 83 places were randomly allocated in statistical blocks to problem‐oriented treatment (22), direct‐saturation patrol treatment (21), and control (40) conditions
	90‐day intervention period		
	No threats to the integrity of the treatment reported		Differences of differences between a number of violent and property crime indicators during 1‐year pretest and 90‐day posttest periods, comparing control and experimental groups
Philadelphia (PA) Foot Patrol Program	Foot patrol in violent crime hot spots	120 violent crime hot spots identified based on spatial and temporal analyses of street violent crime incidents	Randomized controlled trial; 120 places were matched into like pairs based on ranking of violent crime incident volume; control and treatment groups were each randomly allocated 60 places within matched pairs
Ratcliffe et al. (2011)	12‐week intervention period		
	No threats to the integrity of the treatment reported		
			Differences of differences between a number of indicators during 3‐month pretest and intervention periods, comparing control and treatment groups
Boston (MA) Safe Street Teams Program	Problem‐oriented policing interventions comprised of mostly enforcement initiatives and limited situational responses	13 violent crime hot spots based on spatial analyses of violent street crimes and officer perceptions of place boundaries	Quasiexperiment; 564 comparison street units were matched via propensity scores to 478 treatment street units
Braga et al. (2011)	3‐year intervention period		Growth curve regression models were used to estimate intervention effects at treatment street units relative to comparison street units over 10 years
	No threats to the integrity of the treatment reported		
DDACTS Program in Washoe County (NV)	Increased patrol and high visibility traffic enforcement	Two crime and car accident hot spots based on spatial analyses of crime, crash, and traffic‐related data	Quasiexperiment; two treatment areas and two control sites with comparable geographic and economic characteristics
Beck (2010)	Four 1‐week iterations of treatment		
	Little difference in the number of traffic stops between treatment and control areas		ANOVA models were used to estimate intervention effects at treatment locations relative to comparison locations for the 4 weeks before and 4 weeks after treatment
Safer Cities Initiative in Los Angeles (CA)	Zero‐tolerance policing aimed at breaking up homeless encampments	One area with a high concentration of homeless encampments	Quasiexperiment; one treatment division compared with four adjacent divisions with comparable economic and land use conditions
Berk and MacDonald (2010)	Pilot project lasted 124 weeks and full intervention lasted 67 weeks		Count‐based generalized additive model was used in a time series analysis to estimate the impact of the intervention over 417 weeks
	No threats to the integrity of the treatment reported		
LPR Patrols in Crime Hot Spots in Two Adjacent Jurisdictions	Directed patrol with license plate readers	30 auto‐related crime hot spots identified based on computerized mapping and database technology	Randomized controlled trial; 30 places were randomly allocated in statistical blocks to experimental (15) and control conditions (15)
Lum et al. (2011)	Intervention lasted 99 days for APD and 58 days for FCPD	and consultation with police agencies	Count‐based negative binomial regression while controlling for seasonality was used to estimate the impact of the intervention over the pre (99 days for APD; 58 days for FCPD), active (99 days for APD; 58 days for FCPD), and postintervention (30 days) periods
	No threats to the integrity of the treatment reported		
Camden (NJ) 28‐Day Crime Suppression Initiative	Increased high‐visibility uniformed patrol	One crime hot spot identified based on spatial analyses of crime and police patrol patterns	Quasiexperiment; one target area compared with the rest of the city
Ratcliffe and Breen (2011)	7‐week intervention period		Percentage change in crime in treatment and control groups over 7‐week intervals pre‐to‐during and pre‐to‐post‐intervention were used to estimate the impact of the intervention
	No threats to the integrity of the treatment reported		
Predictive Risk Mapping and Policing in Trafford, Greater Manchester	Increased patrol during high‐risk times	52 hot spots identified based on risk‐based computerized mapping	Quasiexperiment; 52 hot spots compared with 52 control hot spots similar to treated areas
Fielding and Jones (2012)	1‐year intervention period		Time series analysis of weekly crime counts before (1 year) and during the intervention (1 year)
	No threats to the integrity of the treatment reported		
Broken Windows Style Crackdowns in Three California Cities	Problem‐oriented policing consisting of strategies to reduce social and physical disorder	110 street segments identified based on spatial analyses of calls for service and crime incident data, and disorder problems	Randomized controlled trial; 110 street segments were randomly allocated in statistical blocks to experimental (55) and control conditions (55)
	7‐month intervention period		
Weisburd et al. (2012)	No threats to the integrity of the treatment reported		ANOVA model was used to estimate the impact of the intervention on treated areas relative to control areas
Operation LASER in Los Angeles (CA)	Location‐based and offender‐focused strategy stressing the removal of repeat offenders and gang members	Five gun violence hot corridors identified based on spatial analyses of crime incident, arrest, and calls for service data	Quasiexperiment; 20 reporting districts receiving both location and offender treatment strategies were compared to 314 control reporting districts
Uchida and Swatt (2013)	10‐month intervention period		
	No threats to the integrity of the treatment reported		Count‐based hierarchical linear models nested within reporting districts were used to estimate the impact of the intervention using monthly crime counts over 78 months
Palos Verdes (NV) Team Policing Project	Problem‐oriented policing consisting of saturation patrol, neighborhood cleanups, and community engagement	One neighborhood selected based on its history of crime and police–community relations problems	Quasiexperiment; one treatment area was compared with three control areas of similar size, demographics, and crime

	9‐month intervention period		Paired sample *t* tests were used to compare crime in treatment and control areas over 18 months
Martinez (2013)	No threats to the integrity of the treatment reported		
LPRs at Crime Hot Spots Experiment in Mesa (AZ)	Increased patrols with and without license plate readers	117 hot routes identified based on spatial analyses of autotheft data and police officer observations	Randomized controlled trial; 117 street segments were randomly allocated in statistical blocks to LPR‐enhanced patrols (45), manual check patrols (45), and control conditions (27)
Koper et al. (2013)	30‐week intervention period		
	No threats to the integrity of the treatment reported		Count‐based random effects panel regression models with controls for seasonality were used to estimate the short‐ and long‐term effects of the intervention over 30 weeks
Lowell (MA) Smart Policing Initiative	Problem‐oriented policing consisting of mostly enforcement actions and some community engagement	24 hot spots were identified based on spatial analyses of crime incident data	Quasiexperiment; 12 treatment hot spots were matched to 12 similar hot spots in control sectors
Bond et al. (2014)			
	16‐month intervention period		
	No threats to the integrity of the treatment reported		Percentage change in crime for treatment and control areas before (16 months) and during the intervention (16 months) was used to examine the impact of the intervention
DDACTS Program in Shawnee (KS)	Increased patrol and high visibility traffic enforcement	One target zone selected based on spatial analyses of crime and accident data	Quasiexperiment; one target zone was compared with one control zone of comparable size, population, land use, and crime
Bryant et al. (2014)	3‐year intervention period		
	No threats to the integrity of the treatment reported		The average number of crimes before (3 years) and during the intervention (3 years) was used to estimate the impact of the intervention
Summer Crime Initiative in Washington, DC	Increased patrol and arrest‐driven targeted enforcement	Five hot spots selected based on spatial analyses of crime incident data and supplemented with input from the Intelligence Unit	Quasiexperiment; five target areas were compared with five control areas with similar socioeconomic and housing characteristics
Mazeika (2014)	3‐month intervention period		
	No threats to the integrity of the treatment reported		*t* tests examining monthly crime counts were used to estimate the impact of the intervention on treated areas relative to control areas over 24 months
Operation Impact in Newark (NJ)	Saturation patrol emphasizing proactive enforcement of street‐level disorder and drug activity	One hot corridor was selected based on spatial and temporal analyses of street violence incident data	Quasiexperiment; one target area compared with the rest of the precinct and one control zone with similar problems
Piza and O'Hara (2014)	1‐year intervention period		Odds ratios were used to compare changes in crime for the target area relative to control areas before (1 year) and during the intervention (1 year)
	No threats to the integrity of the treatment reported		
St. Louis (MO) Metro PD's Firearms Violence Hot Spots Policing Experiment	Increased patrol with and, separately, without self‐initiated enforcement activity	47 hot spots were identified based on spatial analyses of firearm violence	Randomized controlled trial; 32 hot spots were randomly allocated in statistical blocks to enhanced patrol (8), enhanced patrol with self‐initiated activity (8), and control conditions (16)
Rosenfeld et al. (2014)	9‐month intervention period		
	Limited difference in treatment between patrol without self‐initiated enforcement activity and the control condition		Multilevel linear regression was used to compare changes in crime before and during the intervention for both treatment and control areas over 18 months
Hot Spots Randomized Field Trial in Sacramento (CA)	Increased patrol with an emphasis on proactive contact with residents and businesses	52 hot spots were identified based on spatial analyses of calls for service and crime incident data and supplemented with police officer observations	Randomized controlled trial; 42 hot spots were randomly allocated in statistical blocks to experimental (21) and control conditions (21)
Telep et al. (2014)	3‐month intervention period		
	No threats to the integrity of the treatment reported		*t* tests in a difference‐in‐difference design were used to estimate changes in crime in the 3‐month preintervention and 3‐month active intervention periods for both treated and control areas
Trinidad and Tobago Police Services Hotspot Experiment	Increased patrol especially during high‐risk times	Within the 20 treatment districts, hot spots were identified based on spatial analyses of crime incident data	Randomized controlled trial; 40 districts were randomly allocated in statistical blocks to experimental (20) and control conditions (20)
	3‐month intervention period		
Sherman et al. (2014)	No threats to the integrity of the treatment reported		Random effects meta‐analysis models were used to estimate the impact of the intervention on treated areas relative to control areas over 21 months
Policing Crime Hot Spots in Stockholm, Sweden	Problem‐oriented policing consisting of directed patrol, increased investigation, focus on repeat offenders, and community engagement	Hot spots were identified based on spatial and temporal analyses of robberies	Quasiexperiment; seven treatment areas were compared to the rest of the city
Marklund and Merenius (2014)	1‐year intervention period		
	Low treatment dosage reported		*t* tests were used to examine changes in weekly crime rates in the treatment and control areas before and after the intervention
Policing Crime Hot Spots in Eskilstuna, Sweden	Problem‐oriented policing consisting of undercover operations, collaborating with bar owners and employees, and increased monitoring of private security guards	Hot spots were identified based on spatial and temporal analyses of public assaults	Quasiexperiment; three treatment areas were compared to the rest of the city
Marklund and Merenius (2014)	1‐year intervention period		
	No threats to the integrity of the treatment reported		Compared changes in weekly crime rates in treatment and control areas before and after the intervention
Anti‐Drunk Driving Program in Rajasthan, India	Roadblocks targeting drunk driving	213 hot spots were identified based on officer knowledge of areas known for drunk driving accidents	Randomized controlled trial; 213 checkpoint locations were randomly allocated in statistical blocks to experimental (147) and control conditions (66)
Banerjee et al. (2014)	15‐month intervention period		Multilevel OLS regression was used to estimate the impact of the intervention at treated checkpoints relative to control checkpoints
	No threats to the integrity of the treatment reported		
Philadelphia (PA) Policing Tactics Experiment	Problem‐oriented policing consisting mostly of enforcement actions and strategies targeting high‐risk individuals and quality‐of‐life issues	81 hot spots were identified based on spatial analyses of crime and supplemented with input from command staff	Randomized controlled trial; 81 hot spots were randomly allocated in statistical blocks to problem‐oriented policing (20), foot patrol (20), offender‐focused (20), and control conditions (21)
Groff et al. (2015)	Increased foot patrol for 8 hr/day, 5 days per week		
	Offender‐focused strategy targeting high‐risk individuals who reside in hot spots		Multilevel mixed‐effects negative binomial regression was used to longitudinally analyze changes in crime for the treatment and control groups over 38 weeks
	Each tactic was implemented for 12 to 24 weeks		
	Staggered implementation of each tactic and some challenges maintaining treatment integrity		
Colorado Springs (CO) PD's Risk‐Based Intervention	Problem‐oriented policing consisting of mostly proactive enforcement actions, as well as community engagement and neighborhood cleanup	Hot spots were identified based on risk‐based computerized mapping	Quasiexperiment; 144 treatment street units were matched to 144 control street units via propensity score matching
Kennedy et al. (2015)	3.5‐month intervention period		Relative effect size was used to examine changes in crime in the preintervention (3.5 months) and postintervention periods (3.5 months) for target and control areas
	No threats to the integrity of the treatment reported		
Newark (NJ) PD's Risk‐Based Intervention	Problem‐oriented policing consisting of increased police presence and engagement with business owners	Hot spots were identified based on risk‐based computerized mapping	Quasiexperiment; 177 treatment street units were matched to 180 control street units via propensity score matching
Kennedy et al. (2015)	3‐month intervention period		Relative effect size was used to examine changes in crime in the preintervention (3 months) and postintervention periods (3 months) for target and control areas
	No threats to the integrity of the treatment reported		
Kansas City (MO) PD's Risk‐Based Intervention	Problem‐oriented policing consisting of enforcement actions, community engagement, and increased police presence	Hot spots were identified based on risk‐based computerized mapping	Quasiexperiment; 139 treatment street units were matched to 195 control street units via propensity score matching
Kennedy et al. (2015)	3‐month intervention period		
	No threats to the integrity of the treatment reported		Relative effect size was used to examine changes in crime in the preintervention (3 months), during the intervention (3 months), and postintervention periods (3 months) for target and control areas
Glendale (AZ) PD's Risk‐Based Intervention	Increased patrol and proactive enforcement actions	Hot spots were identified based on risk‐based computerized mapping	Quasiexperiment; 37 treatment street units were matched to 141 control street units via propensity score matching
Kennedy et al. (2015)	3‐month intervention period		
	Minor problems with boundary adherence for treatment condition		Relative effect size was used to examine changes in crime in the preintervention (3 months), during the intervention (3 months), and postintervention periods (3 months) for target and control areas
St. Louis County (MO) Hot Spots in Residential Areas Study	Problem‐oriented policing consisting of target hardening, community engagement, and increased interagency coordination	81 hot spots were identified based on spatial analyses of crime incident data and supplemented with input from precinct commanders	Randomized controlled trial; 71 hot spots were randomly allocated in statistical blocks to problem‐oriented policing (20), directed patrol (20), and control conditions (31)
Kochel et al. (2015)	Increased patrol during high‐risk times		
	5‐month intervention period		A time series analysis using ARIMA with controls for seasonality was used to estimate the impact of the intervention on treated areas relative to control areas over 104 weeks
	No threats to the integrity of the treatment reported		
Mobile Computing Technology at Crime Hot Spots in a Suburban County	Increased patrol and proactive enforcement actions	18 high crime street segments were identified based on spatial analyses of crime incident and calls for service data	Randomized controlled trial; 18 hot spots randomly allocated in statistical blocks to experimental (9) and control conditions (9)
Koper et al. (2015)	11‐week intervention period		Negative binomial regression in a longitudinal panel design was used to estimate the impact of the intervention on treated areas relative to control areas over 11 weeks
	Limited resources led to modest treatment dosage; officers did not use technology in anticipated ways		
Proactive CCTV Monitoring with Directed Patrol in Newark (NJ)	Actively monitored CCTV with directed patrol	38 hot spots were identified based on spatial and temporal analyses of calls for service data	Randomized controlled trial; 38 hot spots randomly allocated in statistical blocks to experimental (19) and control conditions (19)
	11‐week intervention period		Negative binomial regression was used to estimate the impact of the intervention changes in crime in treated areas relative to controls over 22 weeks
Piza et al. (2015)	No threats to the integrity of the treatment reported		Examined spatial displacement and diffusion effects in 291‐foot buffer areas around each viewshed
Tactical Police Response at Micro‐Time Hot Spots	Increased patrol at micro‐time hot spots	172 microtime hot spots for residential theft from vehicle crime and 108 microtime hot spots for residential burglary were identified based on near‐repeat spatial and temporal analyses of crime incident data	Quasiexperiment; propensity score matching was used to match 86 treatment areas for residential theft from vehicle crime to 86 control areas and 54 treatment areas for residential burglary to 54 control areas
Santos and Santos (2015a, 2015b)	Treatment delivered in 21 day iterations		
	No threats to the integrity of the treatment reported		
			Independent *t* tests were used to compare differences in crime between treatment and control areas postimplementation (21 days)
Philadelphia GunStat Model	Offender‐focused strategy consisting of aggressive prosecution and monitoring of repeat offenders	Five hot spots were identified based on practitioner knowledge of the spatial distribution of gun crime	Quasiexperiment; treatment locations (Phase 1 = 122; Phase 2 = 196) were matched to control locations (Phase 1 = 122; Phase 2 = 196) via propensity score matching
Sorg (2015)	2‐year intervention period		
	Problems with interagency communication and cross‐district collaboration, lack of enhanced monitoring by probation and parole, and high turnover among project leadership		Negative binomial regression was used to estimate the effects of the intervention on treatment areas relative to control areas over 2 years
Dallas (TX) Patrol Management Experiment	Automated vehicle location technology to increase total and unallocated patrol time at hot spots	1,006 hot spots within 232 police beats were identified by police division commanders	Randomized controlled trial; 232 police beats were randomly allocated in statistical blocks to experimental (116) and control conditions (116)
	13‐week intervention period		
Weisburd et al. (2015)	Less unallocated patrol time directed at hot spots than was anticipated		A *F* test was used to compare changes in crime in treatment and control areas over 13 weeks
West Midlands (England) Police's Randomized Control Trial of Policing Hot Spots	Increased frequency and length of patrol	14 high crime 150 × 150‐m grids were identified based on spatial analyses of street crime and antisocial behavior calls for service data	Small *N* randomized experiment that was analyzed as a quasiexperiment; seven pairs of hot spots were matched and then randomly allocated to treatment and control conditions
	100‐day intervention period		
Williams (2015)	Breakdown in treatment delivery near the end of intervention period led to shortened intervention		Percentage change in crime in treatment and control areas pre (100 days), during (100 days), and postintervention (100 days) was analyzed to estimate the effect of the intervention
Actively Monitored CCTVs in Stockholm, Sweden	Actively monitored CCTV with directed patrol	Two hot spots were identified based on practitioner knowledge of the spatial and temporal distribution of crime	Quasiexperiment; two target areas were compared with five other areas in the city with comparable crime and nightlife activity
	33‐month intervention period		
Marklund and Holmberg (2015)	Limited use of CCTV footage in police investigations		Percentage change in crime in treatment and control areas over 61 months was used to estimate the impact of the intervention
Operation Style in Peterborough, England	Increased presence of uniformed civilian police staff	72 high crime 150‐m radius polygons were identified based on spatial analyses of crime incident data	Randomized controlled trial; 72 hot spots were assigned to treatment (34) and control conditions (38) using simple random assignment
	1‐year intervention period		
Ariel et al. (2016)	Difficult to maintain treatment integrity		Standardized mean difference and OLS regression were used to compare changes in crime before (24 months) and during the intervention (12 months)
Glendale (AZ) Smart Policing Initiative	Problem‐oriented policing consisting of mostly surveillance and enforcement actions	Six high crime convenience stores were identified based on practitioner knowledge of the spatial and temporal distribution of crime	Quasiexperiment; six treatment stores were compared to 68 control stores
Dario (2016)	1‐year intervention period		
	No threats to the integrity of the treatment reported		Negative binomial regression was used to estimate the impact of the intervention on treatment areas relative to control areas by comparing crime between preintervention (31 months) and postintervention (25 months)
Policing Violent Crime Hot Spots in Malmö, Sweden	Actively monitored CCTVs with directed patrol	One high crime entertainment district was identified based on practitioner knowledge of the spatial and temporal distribution of crime	Quasiexperiment; one target area was compared with one control area with similar nightlife
Gerell (2016)	1‐year intervention period		Changes in crime counts before (1 year) and during the intervention (1 year) for treatment and control areas were used to determine the effects of the intervention
	No threats to the integrity of the treatment reported		
Operation Impact in New York City	Increased patrol with proactive enforcement actions	Hot spots were identified based on local commanders' and crime analysts' knowledge of the spatial distribution of crime	Quasiexperiment; treatment precinct‐months were compared with control precinct‐months
	9‐year intervention period		
MacDonald et al. (2016)	No threats to the integrity of the treatment reported		Poisson regression models were used to estimate the impact of the intervention on treated areas relative to control areas over 9 years
Kansas City (MO) Foot Patrol Project	Increased foot patrol for two shifts per day	Eight patrol beats were identified based on practitioner and researcher knowledge of the spatial distribution of crime	Quasiexperiment; four treatment police beats were compared with four control police beats
Novak et al. (2016)	3‐month intervention period		
	No threats to the integrity of the treatment reported		A time‐series analysis with panel‐specific autoregressive models was used to estimate the impact of the intervention by comparing the preintervention period (30 weeks) to the active (13 weeks) and postintervention periods (40 weeks)
Police Paramilitary Raids in Buffalo (NY)	Police paramilitary raids at places known for drug activity	99 high crime locations were identified based on practitioner knowledge of the spatial distribution of violence, shootings, and drug crime	Quasiexperiment; 99 treatment areas were compared to 282 control areas via propensity score matching
	2‐day intervention period		
Phillips et al. (2016)	No threats to the integrity of the treatment reported		Fixed effects negative binomial panel regression was used to estimate the impact of the intervention on target areas relative to control areas across the preintervention (35 weeks) and postintervention periods (35 weeks)
Offender‐Focused Police Intervention at Hot Spots in Port St. Lucie (FL)	Offender‐focused strategy consisting of detectives contacting repeat offenders and strengthened formal surveillance	48 hot spots were identified based analyses of the spatial distribution of crime with consideration of the neighborhood context	Randomized controlled trial; 48 hot spots were randomly assigned in statistical blocks to experimental (24) and control conditions (24)
Santos and Santos (2016)	9‐month intervention period		Negative binomial and OLS regression were used to estimate the impact of the intervention on treated areas relative to controls over the preintervention (9 months) and active intervention periods (9 months)
	No threats to the integrity of the treatment reported		
New Haven (CT) Smart Policing Initiative	Problem‐oriented policing consisting of directed patrol, problem solving, and community engagement	One high crime neighborhood identified based on spatial analyses of violent crime and calls for service data	Quasiexperiment; 1 treatment neighborhood compared to 4 control neighborhoods with similar crime and socioeconomic characteristics
Sedelmaier and Hipple (2016)	13‐week intervention period		
	No threats to the integrity of the treatment reported		Changes in crime counts pre (13 weeks), during (13 weeks), and postintervention (13 weeks) were examined to estimate the impact of the intervention
Operation Menas in London, England	Increased police presence of teams of two uniformed officers	102 high crime bus stops were identified based on spatial and temporal analyses of crime incident data	Randomized controlled trial; 102 bus stops were assigned to experimental (51) and control conditions) using simple random assignment
Ariel and Partridge (2017)	6‐month intervention period. No threats to the integrity of the treatment reported		
		Each bus stop included a 50‐m buffer	Count‐based adjusted Poisson regression was used to estimate the impact of the intervention before (6 months) and during the intervention (6 months)
Investigating Hot Spots Policing in Copenhagen, Denmark	Problem‐oriented policing consisting of visible patrol, removal of physical disorder, increased surveillance, and community engagement	31 hot spots were identified based on spatial and temporal analyses of crime incident data	Randomized controlled trial; 31 hot spots were randomly assigned in statistical blocks to experimental (15) and control conditions (16)
Attermann (2017)	9‐month intervention period		OLS regression was used to estimate the impact of the intervention on treated areas relative to control areas during the before (8 months) and during the intervention (8 months)
	No threats to the integrity of the treatment reported		
Hot Spots Policing in Bogotá, Colombia	Problem‐oriented policing consisting of directed patrol and municipal services to address disorder	1,919 high crime street segments were identified based on spatial analyses of crime incident data and supplemented with input from command staff	Randomized controlled trial; 1,919 street segments were randomly assigned in statistical blocks to experimental (756) and control conditions (1,163)
Blattman et al. (2017)	Increased police presence		Weighted least squares regression accounting for randomization interference and inverse probability weights was used to estimate the impact of the intervention on treated areas relative to control areas over the 8‐month intervention period
	8‐month intervention		
	Negligible visual improvements from increased municipal services		
Philadelphia (PA) Predictive Policing Experiment	Increased uniformed and, separately, unmarked patrol	Three 500 by 500 feet high crime grids for each of the 20 police districts were identified based on risk‐based computerized mapping	Randomized controlled trial; 20 police districts randomly assigned to 1 of 4 conditions: control, awareness, enhanced marked patrol, and enhanced unmarked patrol
Ratcliffe et al. (2017)	Increased information sharing with patrol officers		
	3‐month intervention period		Negative binomial regression was used to estimate the impact of the intervention over 90 days
	Challenges with software and resource availability		
Flint (MI) DDACTS Program	Saturation patrol with high‐visibility traffic enforcement	Seven crime hot spots were identified based on spatial analyses of crime incident and accident data	Quasiexperiment; 1,117 treated blocks were compared to 2 control groups: other blocks in the same city (1,888) and blocks in a different city (13,097)
Rydberg et al. (2017)	27‐month intervention period		
	No threats to the integrity of the treatment reported		Fixed effects meta‐analysis models were used to estimate the impact of the intervention on treated areas relative to control areas before (3 years) and during the intervention (3 years)
Operation Strikeforce in Buffalo (NY)	Traffic roadblocks with automated license plate readers	46 high crime locations were identified based on spatial analyses of crime incident data	Quasiexperiment; 328 treated segments were compared to 328 control segments via propensity score matching
Wheeler and Phillips (2018)	2‐month intervention period		
	No threats to the integrity of the treatment reported		T‐tests of mean differences and negative binomial regression were used to estimate impact of the intervention on treated areas relative to control areas between pre (39 months) and postintervention periods (10 months)

**Table 3:** Results of hot spots policing experiments and quasiexperiments

Study	Crime outcomes	Other outcomes	Displacement/diffusion
Minneapolis (MN) RECAP	No statistically significant differences in the prevalence of citizen calls for service at commercial addresses	None	Not measured
Sherman et al. (1989)	Statistically significant 15% reduction in calls for service at residential address in the first 6 months that decline to 6% in the first full year		
New York (NY) Tactical Narcotics Teams	No statistically significant reductions in assault, robbery, and burglary incidents in the 70th precinct	Prepost community survey and interviews suggested that TNT did not improve community perceptions of disorder, reduce fear of crime, increase use of public amenities, or improve community attitudes toward the police	Not measured
Sviridoff et al. (1992)	In the 67th precinct, there was a statistically significant reduction in assault incidents; no statistically significant reductions in robbery or burglary incidents		
St. Louis (MO) POP in 3 Drug Areas	All three drug locations experienced varying reductions in total calls	None	Compared trends in calls at targeted addresses to trends in calls at other addresses on same block
Hope (1994)	Regression analysis suggests that reductions on blocks where drug locations were located were greater than other blocks and intersections in surrounding areas		Location 1—significant displacement into surrounding addresses; Location 2—no displacement or diffusion; Location 3—no displacement or diffusion
Minneapolis (MN) Hot Spots	Modest, but statistically significant reductions in total crime calls for service ranging from 6% to 13%	Systematic observations of crime and disorder were half as prevalent in experimental as in control hot spots	Not measured
Sherman and Weisburd (1995)			
Jersey City (NJ) DMAP	Statistically significant reductions in disorder calls for service in treatment drug markets relative to control drug markets	None	Examined displacement and diffusion effects in two‐block catchment areas surrounding the treatment and control drug places and replicated the drug market identification process
Weisburd and Green (1995b)	No change in violent and property crime calls		Little evidence of displacement; analyses suggest modest diffusion of benefits for disorder
Kansas City (MO) Gun Project	65% increase in guns seized by the police; 49% decrease in gun crimes in treatment area	Separate pre/post quasiexperiment surveying citizens opinions of KC gun project suggests citizens were aware of the project, generally supported the intensive approach, and perceived an improvement in the quality of life in treatment neighborhood compared to residents in comparison beat	Displacement tests using pre/post difference in means and ARIMA time‐series analyses were conducted in seven contiguous beats
Sherman and Rogan (1995a)	15% reduction in guns seized by the police; 4% increase in gun crimes in control area		No significant displacement into specific beats; two beats showed significant reductions in gun crimes
Kansas City (MO) Crack House Raids	Modest decreases in citizen calls and offense reports that decayed in 2 weeks	None	Not measured
Sherman and Rogan (1995b)			
Beenleigh (AUS) Calls for Service Project	No noteworthy differences in total number of calls between Beenleigh and Browns Plains areas	None	Not measured
Criminal Justice Commission (1998)	Noteworthy reductions in calls reported by nonexperimental pre/post impact assessments in 16 of the 19 case studies		
Jersey City (NJ) POP at Violent Places	Statistically significant reductions in total calls for service and total crime incidents	Observation data revealed that social disorder was alleviated at 10 of 11 treatment places relative to control places	Examined displacement and diffusion effects in two‐block catchment areas surrounding the treatment and control violent places
Braga et al. (1999)	All crime categories experienced varying reductions; statistically significant reductions in street fight calls, property calls, narcotics calls, robbery incidents, and property crime incidents	Nonexperimental observation data revealed that physical disorder was alleviated at 10 of 11 treatment places	Little evidence of immediate spatial displacement or diffusion
		Nonexperimental interviews with key community members in target locations suggest no noteworthy improvements in citizen perceptions of places	
Houston (TX) Targeted Beat Program	Aggregated experimental beats experienced significant reductions in auto theft, total Part I Index crimes, and total Part I suppressible (robbery, burglary, auto theft) index crimes relative to aggregate control beats	None	Simple pre/post analyses of reported crimes in beats contiguous to treatment beats
Caeti (1999)	Three “zero tolerance” beats experienced mixed results; certain reported crimes decreased in particular beats		No evidence of significant displacement; contiguous beats surrounding three target areas (problem‐solving beat, 2 zero‐tolerance beats) experienced possible diffusion of benefits in particular reported crime
	Three “high visibility” beats experienced reductions in a wide variety of Index crimes		
	Problem solving beat experienced no significant decrease relative to control beat		
Oakland (CA) Beat Health Program	Statistically significant reductions in drug calls in treatment blocks relative to control blocks; no statistically significant differences in other call types	None	Examined displacement and diffusion effects in 500 foot radii catchment areas surrounding the treatment and control street blocks
Mazerolle et al. (2000)			
			Analyses of catchment areas suggested an overall diffusion of crime control benefits for treatment catchment areas relative to control catchment areas
Pittsburgh (PA) Police Raids at Nuisance Bars	Statistically significant reductions in drug calls in treatment bar areas relative to control bar areas that largely disappeared when intervention ceased	None	Not measured
Cohen et al. (2003)			
Buenos Aires (ARG) Police Presence after Terrorist Attack	Statistically significant 75% reduction in motor vehicle thefts	None	Examined displacement and diffusion effects in blocks that were one and two blocks away from treatment blocks
DiTella and Schargrodsky (2004)			No evidence of immediate spatial displacement or diffusion
Philadelphia (PA) Drug Corners Crackdowns	Statistically significant reductions in violent crime incidents and drug crime incidents in treatment areas; no statistically significant changes in violent crime incidents and drug crime incidents in comparison areas	None	ARIMA analyses of 0.1 buffer areas surrounding targeted locations
Lawton et al. (2005)			A significant reduction in violent crime incidents
			Mixed findings for drug crime incidents
Jersey City (NJ) Displacement and Diffusion Study	Statistically significant 45% reduction at the targeted prostitution location	Ethnography and interviews with arrested offenders confirmed that offenders did not displace from targeted locations into surrounding areas	Examined displacement and diffusion effects in one and two block catchment areas surrounding targeted locations
Weisburd et al. (2006)	Statistically significant 58% reduction at the targeted drug crime location		Analyses revealed significant diffusion of crime control benefits
Lowell Policing Crime and Disorder Hot Spots Project	Statistically significant reductions in total calls for service	Observation data revealed that social disorder was alleviated at 14 of 17 treatment places relative to control places	Examined displacement and diffusion effects in two‐block catchment areas surrounding the treatment and control violent places
Braga and Bond (2008)	All crime categories experienced varying reductions; statistically significant reductions in street fight calls, property calls, narcotics calls, robbery incidents, and property crime incidents	Observation data revealed that physical disorder was alleviated at 13 of 17 treatment places relative to control places	No evidence of immediate spatial displacement or diffusion
		Pre‐ and posttest interviews with key community members in treatment and control locations suggest that disorder problems were positively impacted	
Jacksonville (FL) Policing Violent Crime Hot Spots Program Taylor et al. (2011)	Problem‐oriented policing generated statistically significant 33% reduction in street violence	None	Examined displacement and diffusion effects in 500 feet buffer zones surrounding the treatment and control violent places
	Direct‐saturation patrol did not generate any statistically significant reductions		Evidence of immediate spatial displacement associated with problem‐oriented policing intervention
Philadelphia (PA) Foot Patrol Program	Statistically significant 23% reduction in street violent crime incidents	None	Examined displacement and diffusion effects in buffer zones constructed by the research team
Ratcliffe et al. (2011)			Evidence of immediate spatial displacement associated with foot patrol; however, the net benefit of foot patrol in reducing violent crime exceeded the displacement effect
Boston (MA) Safe Street Teams Program	Statistically significant 14% reduction in violent crime incidents	None	Examined displacement and diffusion effects in two‐block catchment areas surrounding the treatment and control street units
Braga et al. (2011)			No evidence of immediate spatial displacement or diffusion
DDACTS Program in Washoe County (NV)	No significant changes in any crime outcomes examined	None	Not measured
Beck (2010)			
Safer Cities Initiative in Los Angeles (CA)	Statistically significant decreases of 30% for nuisance crime, 39% violent crime, and 35% property crime	None	Examined displacement and diffusion effects in the four police divisions surrounding the treated division
Berk and MacDonald (2010)			Evidence of an immediate spatial diffusion of benefits
LPR Patrols in Crime Hot Spots in Two Adjacent Jurisdictions	No significant changes in total crime, auto theft, and autorelated crime	None	Not measured
Lum et al. (2011)			
Camden (NJ) 28‐Day Crime Suppression Initiative	Percentage changes pre‐ to postintervention periods, results favored treatment group for violent crime, drug crime, vehicle crime, and burglary	None	Examined displacement and diffusion effects in several blocks surrounding the treated area using the weighted displacement quotient
Ratcliffe and Breen (2011)			Evidence of a spatial diffusion of benefits for violent crime, burglary, and drug crime
			Evidence of spatial displacement for vehicle crime
Predictive Risk Mapping and Policing in Trafford, Greater Manchester	Statistically significant 45% to 53% declines in burglary in treated high‐risk areas	None	Not measured
Fielding and Jones (2012)			
Broken Windows Style Crackdowns in Three California Cities	No significant change in calls for service	Surveys of 371 residents in treated and control areas before and after the intervention	Not measured
Weisburd et al. (2012)		Nonsignificant improvements in treated residents' perceptions of crime levels	
		No significant differences in fear of crime, collective efficacy, and perceptions of disorder	
Operation LASER in Los Angeles (CA)	Statistically significant 5% reduction in monthly gun crime for the overall intervention	None	Not measured
Uchida and Swatt (2013)	Statistically significant 7% reduction in gun crime for areas that received both types of treatment		
Palos Verdes (NV) Team Policing Project	Target neighborhood experienced statistically significant increases in violent and total calls for service	None	Examined spatial displacement and diffusion effects in a neighborhood nearby the treated area
Martinez (2013)	Target neighborhood did not experience significant changes for property crime and disorder offenses		No evidence of spatial displacement or diffusion
LPRs at Crime Hot Spots Experiment in Mesa (AZ)	For LPR treatment group, significant decreases in short‐ and long‐term of 28% and 49%, respectively experienced	None	Examined spatial displacement and diffusion effects in street routes adjacent to treated routes
Koper et al. (2013)	For manual check treatment group, significant short‐term 35% increase in drug calls for service and significant long‐term decreases of 75% for auto theft calls and 46% for person crimes		Evidence suggest spatial displacement effects for person and disorder crimes
Lowell (MA) Smart Policing Initiative	Hot spots in treatment sectors experienced declines of 16% to 19%, whereas comparison hot spots experienced a 5% increase to 14% decrease	None	Not measured
Bond et al. (2014)			
DDACTS Program in Shawnee (KS)	Treatment zone experienced a statistically significant 40% decrease in total target crimes and marginally significant 70% decrease in robbery, as well as nonsignificant declines in vehicle burglary, vehicle theft, and collisions	Postintervention survey of businesses and residents	Examined displacement and diffusion effects in a community adjacent to the treated area
Bryant et al. (2014)	No significant changes in crime in the control zone	Respondents reported improved quality of life	Evidence of diffusion effects for vehicle theft and total targeted crime
Summer Crime Initiative in Washington, DC	Between the pre and active intervention periods, citizen‐generated robbery calls for service decreased significantly in the target area and increased in the control area	None	Examined spatial displacement and diffusion in the two‐block radius surrounding the treatment area
			Examined offender displacement in a cohort of repeat offenders
Mazeika (2014)	Between the pre‐ and postintervention periods, citizen‐generated robbery calls for service increased in the target area and decreased in the control area		Little evidence of spatial or offender displacement
Operation Impact in Newark (NJ)	Statistically significant declines of 30% for overall violence and 61% for aggravated assault in the target area relative to the control zone	None	Examined spatial displacement and diffusion effects in a one‐block radius surrounding the treatment area
Piza and O'Hara (2014)			Examined temporal displacement and diffusion effects in the hours the operation was not active
			Evidence of spatial diffusion for overall violence, aggravated assault, and shootings; Evidence of spatial displacement for robbery
	Nonsignificant decreases in favor of treatment for murder, shootings, and robbery		Evidence of temporal diffusion for overall violence, murder, aggravated assault, and shootings; evidence of temporal displacement for robbery
St. Louis (MO) Metro PD's Firearms Violence Hot Spots Policing Experiment	For directed patrol with self‐initiated activity, statistically significant reduction in gun assault rates and marginally significant reduction in overall gun violence	None	Examined spatial displacement and diffusion effects in 500‐foot buffer zones surrounding treated areas
Rosenfeld et al. (2014)	For directed patrol without self‐initiated activity, no significant changes in any outcome		Examined offense displacement and diffusion effects for nonfirearm assault
			Examined temporal displacement and diffusion effects for hours when the operation was not active
			No evidence of any form of displacement
Hot Spots Randomized Field Trial in Sacramento (CA)	Statistically significant reduction in calls for service and Part I crime	None	Examined spatial displacement and diffusion in two‐block catchment areas surrounding treatment and control hot spots
Telep et al. (2014)	No significant changes in soft crime incidents		Evidence of spatial displacement for calls for service and Part I incidents
Trinidad and Tobago Police Services Hotspot Experiment	Statistically significant overall decrease in murders and shootings	None	Not measured
Sherman et al. (2014)			
Policing Crime Hot Spots in Stockholm, Sweden	No significant differences in robberies were observed	None	Examined temporal displacement and diffusion effects for the nights the operation was not active
Marklund and Merenius (2014)			No evidence of temporal displacement
Policing Crime Hot Spots in Eskilstuna, Sweden	16% reduction in public assaults in target areas	None	Not measured
Marklund and Merenius (2014)			
Anti‐Drunk Driving Program in Rajasthan, India	Statistically significant 17% reduction in nighttime accidents and 25% reduction in nighttime deaths	None	Not measured
Banerjee et al. (2014)			
Philadelphia (PA) Policing Tactics Experiment	For offender‐focused treatment group, statistically significant 42% decrease in total violent crime and 50% decrease in violent felonies	None	For offender‐focused treatment group, examined displacement and diffusion effects in the two‐block radius surrounding treated hot spots
Groff et al. (2015)	For problem‐oriented policing and foot patrol treatment groups, no significant changes in the outcomes		Evidence of diffusion for violent crime and violent street felonies
Colorado Springs (CO) PD's Risk‐Based Intervention	Marginally significant 33% decrease in motor vehicle theft	None	Examined spatial displacement and diffusion effects in the street units surrounding treated street units
Kennedy et al. (2015)			Evidence of a slight diffusion effect
Newark (NJ) PD's Risk‐Based Intervention	Marginally significant 35% decrease in gun violence	None	Examined spatial displacement and diffusion effects in the street units surrounding treated street units
Kennedy et al. (2015)			Evidence of a slight diffusion effect
Kansas City (MO) PD's Risk‐Based Intervention	Nonsignificant 12% decrease in aggravated violence	None	Not measured
Kennedy et al. (2015)			
Glendale (AZ) PD's Risk‐Based Intervention	Marginally significant 42% decrease in robbery	None	Examined spatial displacement and diffusion effects in the street units surrounding treated street units
Kennedy et al. (2015)			Evidence of a slight diffusion effect
St. Louis County (MO) Hot Spots in Residential Areas Study	For directed patrol treatment group, statistically significant 5% decrease in calls for service	Pre‐ and postintervention panel survey of residents living in hot spots	Not measured
Kochel et al. (2015)	For problem‐oriented policing treatment group, statistically significant 7% decrease in calls for service	For directed patrol, significant short‐term decreases in procedural justice and trust relative to the control group, as well as a nonsignificant decrease in police legitimacy	
		For problem‐oriented policing, no significant short‐term changes in any community outcomes	
		In the long‐term, residents in areas that received either treatment were more likely to cooperate with police	
Mobile Computing Technology at Crime Hot Spots in a Suburban County	Nonsignificant 11% decrease in crime incidents	None	Not measured
Koper et al. (2015)	Marginally significant 24% decrease in crime incidents for high dosage experimental areas		
Proactive CCTV Monitoring with Directed Patrol in Newark (NJ)	Statistically significant 48% decrease in violent crime and 49% decrease for social disorder during tours when operation was active	None	Examined spatial displacement and diffusion effects in 291‐foot buffer areas around each viewshed
Piza et al. (2015)	Nonsignificant reduction in drug crime		Examined temporal displacement and diffusion effects in the hours and days when the operation was not active
			Total net effects show declines in violent crime during the tours and days the intervention was active, as well as social disorder for the full intervention period
Tactical Police Response at Micro‐Time Hot Spots	Statistically significant 20% in residential theft from vehicle crime	None	Examined spatial displacement and diffusion effects in a 0.2‐mile catchment area surround the treated hot spot
Santos and Santos (2015a, 2015b)	Statistically significant 1.15 fewer residential burglary offenses per microtime hot spot relative to controls		No evidence of spatial displacement
Philadelphia GunStat Model	For Phase I, target locations experienced significant 5% to 29% increases in violent crime and 6% to 64% increases in violent street felonies relative to control locations	None	Not measured
Sorg (2015)	For Phase II, no significant effects on violent crime or violent street felonies		
Dallas (TX) Patrol Management Experiment	Statistically significant 21% decrease in total crime in the treatment hot spots relative to control hot spots	None	Not measured
Weisburd et al. (2015)	No significant differences in crime at the beat‐level		
West Midlands (England) Police's Randomized Control Trial of Policing Hot Spots	Treatment hot spots experienced a 14% reduction in street crimes and antisocial behavior calls for service relative to control hot spots	Examined crime severity by using the Crime Harm Index	Examined displacement and diffusion effects in 150 by 150‐m grids surrounding the targeted hot spots
Williams (2015)		Crime Harm Index increased in the treatment areas	Evidence of diffusion effects for street crime and antisocial behavior calls for service
Actively Monitored CCTVs in Stockholm, Sweden	The two target areas experienced decreases of 58% and 62% for sex offenses whereas the decrease in control areas was 18%	None	Examined temporal displacement and diffusion effects for the hours when the operation was not active
Marklund and Holmberg (2015)	28% decrease in total crime for control areas was greater than 15% and 26% decreases in the two target areas		Limited evidence of temporal displacement for total crime
Operation Style in Peterborough, England	Statistically significant decreases of 39% for total crime and 20% for emergency calls for service in the treatment hot spots relative to control hot spots	Examined crime severity by using the Crime Harm Index	Examined spatial displacement and diffusion effects in the 50‐m radius surrounding the hot spots
Ariel et al. (2016)		Each minute of soft patrol per day was associated with up to a 26‐day reduction in imprisonment in the treatment group relative to control group	Evidence of a spatial diffusion of benefits
Glendale (AZ) Smart Policing Initiative	Statistically significant 16% reduction in calls for service at treatment stores relative to all control stores	None	Examined spatial displacement and diffusion effects in the 500‐yard area surrounding treatment stores
Dario (2016)			Evidence of a spatial diffusion of benefits
Policing Violent Crime Hot Spots in Malmö, Sweden	Nonsignificant decrease in public assaults in treatment area relative to control area when CCTV cameras were actively monitored	None	Examined temporal displacement and diffusion effects in the hours and days the operation was not active
Gerell (2016)			Nonsignificant increase in public assaults during nonoperational times
Operation Impact in New York City	Statistically significant 12% decrease in expected monthly total crime associated with treatment	None	Examined spatial displacement and diffusion effects in blocks immediately adjacent to impact zones
MacDonald et al. (2016)	Statistically significant decreases of 16% for robbery, 13% for assaults, and 46% for burglary in favor of treatment relative control		Evidence of a spatial diffusion effect for total monthly crime
	Statistically significant increases in weapons and other felony offenses in treated zones		
Kansas City (MO) Foot Patrol Project	Statistically significant reduction in violent crime in favor of treatment over first 30 days	None	Examined spatial displacement and diffusion effects in the two‐block catchment area surrounding target areas
Novak et al. (2016)	Nonsignificant reduction in violent crime in favor of treatment over entire study period		No evidence of spatial displacement
Police Paramilitary Raids in Buffalo (NY)	Statistically significant increases in calls for service and drug arrests in target areas relative to control areas	None	Not measured
Phillips et al. (2016)	Nonsignificant decrease in Part I violent and Part I nonviolent crime in target areas relative to control areas		
Offender‐Focused Police Intervention at Hot Spots in Port St. Lucie (FL)	No statistically significant difference in residential burglary and theft from vehicle crime	None	Not measured
Santos and Santos (2016)	Nonsignificant decrease in arrests and rearrests in target areas relative to control areas		
New Haven (CT) Smart Policing Initiative	Target hot spots experienced decreases of 47% for total crime and 72% for violent crime whereas control hot spots experienced a 19% decrease in total crime and 6% decrease in violent crime	None	Not measured
Sedelmaier and Hipple (2016)			
Operation Menas in London, England	Marginally significant 37% reduction in driver incident reports in target areas relative to control areas during active operation hours	None	Examined spatial displacement and diffusion effects in the 50‐ to 100‐m catchment zone surrounding targeted bus stops
Ariel and Partridge (2017)	Nonsignificant 25% increase in victim‐generated crime in target areas relative to control areas		Examined temporal displacement and diffusion effects in the hours and days when the operation was not active
			Evidence of spatial diffusion effects for driver incident reports and limited evidence of displacement for victim‐generated crime during active operation hours
			Limited evidence of temporal diffusion effects for driver incident reports and limited evidence of displacement for victim‐generated crimes
Investigating Hot Spots Policing in Copenhagen, Denmark	Statistically significant reductions in vandalism and motor vehicle crime in target areas relative to control areas	None	Not measured
Attermann (2017)	No significant differences in violence, robbery, shoplifting, other theft, and total street crime		
Hot Spots Policing in Bogotá, Colombia	Nonsignificant reduction in total crime in favor of intensive patrol relative to the control group	Survey of residents in hot spots	Examined spatial displacement and diffusion effects in the 250‐m catchment area surrounding targeted street segments
Blattman et al. (2017)		Marginally significant reduction in feelings of insecurity for residents in areas that received both intensive patrol and municipal services treatment relative to residents in control areas	
	Marginally significant reduction in total crime associated with groups that received both intensive patrol and municipal services treatment relative to the control group	Nonsignificant reduction in feelings of insecurity for residents in areas that received intensive patrol only relative to control areas	Evidence of spatial displacement of property crime for intensive policing treatment that was greater than crime reduction benefits in target areas
		Neither treatment associated with changes in opinions of police	
		Limited evidence that intensive policing was associated with more negative perceptions of the Mayor's Office	Mixed results of spatial displacement for violent crime
Philadelphia (PA) Predictive Policing Experiment	Statistically significant 31% decrease in property crime for the uniformed patrol treatment group relative to the control group	None	Examined temporal displacement and diffusion effects in the 8 hr after the treatment shift
Ratcliffe et al. (2017)	No significant change in property crime associated with either the awareness only and unmarked patrol treatment groups		Examined spatial displacement and diffusion effects by including 500 by 500‐foot grids surrounding target areas
	None of the three treatment groups were significantly related to violent crime		Evidence of temporal diffusion effects for property crime for the uniformed patrol treatment group
Flint (MI) DDACTS Program	Statistically significant 24% decrease in robbery in target areas relative to Detroit comparison areas	None	Not measured
Rydberg et al. (2017)	Statistically significant 18% increase in overall violence in target areas relative to Flint comparison areas		
	Statistically significant increases of 26% to 33% for target areas relative to control areas		
Operation Strikeforce in Buffalo (NY)	Statistically significant increase in Part I violent and Part I nonviolent crime in the target areas relative to control areas	None	Not measured
Wheeler and Phillips (2018)	Statistically significant 20% reduction in traffic accidents in target areas relative to control areas		

#### 5.2.1 Community reactions to hot spots policing programs

Only seven of the 65 eligible studies (10.8%) considered the effects of hot spots policing strategies on police–community relations. For the Kansas City Gun Project, community members exposed to treatment indicated that they welcomed concentrated police efforts at problem places (Shaw, 1995). Residents in treated areas of the Lowell Policing Crime and Disorder Hot Spots experiment reported that they recognized the intervention and its positive impacts on local disorder problems (Braga & Bond, 2009). Results from the Jersey City Problem‐Oriented Policing in Violent Places experiment suggested that community members' improved perceptions of disorder were attributed to the focused intervention and their attitudes toward police were not negatively affected (Braga, 1997).

A “broken windows” style hot spots experiment in three California cities found the disorder‐oriented intervention did not produce a “backfire effect” as it pertains to residents' fear of crime, police legitimacy, collective efficacy, or perceptions of crime or social disorder (Weisburd, Hinkle, Famega, & Ready, 2011). However, a companion analysis to the Weisburd et al. (2006) Jersey City Displacement and Diffusion study suggested that the increased police activity associated with the intervention may have made residents feel less safe (Hinkle & Weisburd, 2008). The Data‐Driven Approach to Crime and Traffic Safety program in Shawnee (KS) found local businesses and community members both reported seeing an increase in high visibility police presence during the intervention and the majority of those who were familiar with initiative believed that it improved the quality of life in the area (Bryant et al., 2014). Evidence from the St. Louis County Hot Spots in Residential Areas experiment suggested the directed patrol treatment was associated with short‐term detriments to police–community relations, but no negative short‐term effects were linked to the problem‐solving treatment (Kochel & Weisburd, 2017). In the long term, for both treatment conditions, residents' willingness to cooperate with police was higher after the intervention ended (Kochel & Weisburd, 2017).

### 5.3 Study implementation

The majority of the eligible hot spots policing studies seemed to implement the desired treatment successfully. Twenty‐one studies (32.3% of 65), however, did report potential threats to the integrity of the treatment spanning various degrees of severity. The Minneapolis RECAP experiment showed no statistically significant differences in the prevalence of citizen calls for service at commercial addresses that received the POP treatment as compared to control commercial addresses (Sherman et al., 1989). Buerger (1993) speculated that these results were probably due to the assignment of too many cases to the RECAP unit, thus outstripping the amount of resources and attention the police officers provided to each address. Moreover, the simple randomization procedure led to the placing of some of the highest event addresses into the treatment group; this led to high variability between the treatment and control groups and low statistical power. Although the overall findings suggest that the RECAP program was not effective in preventing crime, a case study analysis revealed that several treated addresses experienced dramatic reductions in total calls for service (Buerger, 1992).

The Vera Institute of Justice evaluation of the Tactical Narcotics Teams noted that the intervention was not implemented as planned in one of the two treatment precincts (Sviridoff et al., 1992). In the 67th precinct, 20% of the staffing of the Tactical Narcotics Team was reassigned to another department initiative. As a result, the treatment in the 67th precinct yielded fewer arrests and the maintenance period for targeted drug hot spots by uniform patrol was shortened when compared to the treatment in the 70th precinct.

The patrol treatment in the Minneapolis Hot Spots experiment (Sherman & Weisburd, 1995, pp. 638–639) was disrupted during summer months due to a peak in the overall calls for service received by the Minneapolis Police Department and a shortage of officers due to vacations; this situation was further complicated by changes in the computerized calls for service system implemented in the fall. The changes in the calls for service system and the disappearance of differences in patrol dosage between treatment and control hot spots during summer months were addressed by conducting separate outcome analyses using different intervention time periods; there were no substantive differences in the outcomes of the experiment across the different time periods.

The Jersey City DMAP experiment (Weisburd and Green 1995a, p. 721) and Jersey City POP at Violent Places experiment (Braga, 1997, pp. 107–142) reported instances where the treatments were threatened by subversion by the participants. The officers charged with preventing crime at the treatment hot spots were resistant to participating in the programs and this resulted in low levels of treatment during the early months of both experiments. In the Jersey City DMAP experiment, this situation was remedied by providing a detailed crackdown schedule to the Narcotics Squad commander and extending the experiment from 12 to 15 months. This problem was remedied in the Jersey City POP experiment by changing the leadership of the POP unit, developing an implementation accountability system, and providing additional training in the POP approach, in addition to other smaller adjustments.

The Philadelphia Policing Tactics randomized experiment noted deficiencies in both the foot patrol and POP treatment conditions (Groff et al., 2015, pp. 44–45). For the foot patrol treatment, there was not a significant increase in police activity in the targeted areas. The POP treatment suffered due to a lack of commitment to the problem‐solving process and POP officers being pulled from the treatment hot spots to deal with issues elsewhere in the city. Similarly, varying levels of POP activities were also reported across treatment stores in the Glendale (AZ) Smart Policing Initiative quasiexperiment (Dario, 2016).

The Houston Beat Patrol Program reported that the three “high visibility” patrol beats managed by one substation experienced police resistance to the program (Caeti, 1999). However, the evaluation suggested that the treatment was applied with enough integrity to measure possible impacts on reported crime outcomes. In the Jersey City Displacement and Diffusion Study, focused police attention was originally applied to three crime hot spots; unfortunately, the Police Foundation research team detected that the intervention was not being applied with an adequate dosage in the burglary hot spot and, as such, the location was dropped from the evaluation (Weisburd et al., 2006). In the Peterborough “soft” hot spots policing experiment, Ariel et al. (2016: 310) reported a mild threat to the integrity of treatment as it was difficult for the officers to stay within the hot spot boundaries to ensure the consistent delivery of 15‐min patrols, three times per shift, over the entire duration of the study period. Similarly, Kennedy et al. (2015, p. 14) reported officers participating in the Glendale (AZ) quasiexperimental risk‐based intervention did not strictly adhere to the target area boundaries; in response, the quasiexperimental evaluation expanded its definition of treated areas as street segments that experienced at least one intervention activity.

As described in Table 3 and Appendix D, several studies tested new technological innovations designed to increased police presence and enforcement activities in treatment hot spots relative to control hot spots. In four studies, technological failures were noted as possible threats to treatment integrity. Marklund and Holmberg (2015) reported that low‐quality video footage from CCTVs placed in hot spots hampered police investigations of offenders frequenting targeted areas. In the Philadelphia Predictive Policing randomized experiment, Ratcliffe et al. (2017) reported that officers experienced challenges when attempting to access the software. In the Trinidad and Tobago directed patrol randomized experiment, Sherman et al. (2014) documented problems with the GPS technology used to monitor treatment dosage. Finally, in the West Midlands hot spots experiment, the treatment was originally planned to be a 150‐day intervention but a breakdown in “geofencing” over the last 50 days limited the analysis to the first 100 days of intervention.

The treatment delivered in the Philadelphia Police Department's GunStat program suffered from a number of serious implementation issues; Sorg (2015) noted a lack of collaboration across policing districts (p. 175), the withholding of intelligence on repeat offenders frequenting hot spots locations (p. 176), unstable program leadership during the study period (p. 177), and a lack of support from partnering criminal justice agencies (pp. 182–183). Most of the other hot spots policing experiments reporting threats to the integrity of treatment raised questions on dosage such as no differences in police stop rates between treatment and control locations in the Washoe County Data‐Driven Approaches to Crime and Traffic Safety quasiexperiment (Beck, 2010), negligible physical improvements noted in the municipal services hot spots in the Bogotá Hot Spots Policing experiment (Blattman et al., 2017), lower levels of police presence in treatment areas than anticipated in the Dallas (Weisburd et al., 2015) and mobile computing in suburban hot spots (Koper et al., 2015) randomized experiments, and fewer contacts with offenders in targeted hot spots in the Stockholm quasiexperiment (Marklund & Merenius, 2014). Finally, in the St. Louis Gun Violence Hot Spots experiment, Rosenfeld et al. (2014) noted that although the directed patrol with self‐initiated activity treatment was implemented with strong fidelity, the fidelity for directed patrol without self‐initiated activity was limited.

Of course, these implementation problems are not unique to these hot spots policing experiments and quasiexperiments; many well‐known criminal justice field experiments have experienced and successfully dealt with methodological difficulties.^7^ It is also important to note here that none of the eligible studies noted problems with attrition. Since the units‐of‐analysis were places, this may have diminished common attrition issues commonly found in evaluations involving people as the units‐of‐analysis.

### 5.4 Risk of bias in included studies

Table 4 presents our assessment of risk of bias in the *N *= 65 included hot spots policing studies. We assessed the level of risk of bias along six sources of potential bias for each study (“Low” or “High”), or if a study was not clear on whether the bias was present or not (“Unclear”). The dimensions of bias assessed were: (a) to what extent was the random allocation sequence adequately generated? (b) How well was the randomization sequence followed? (c) What was the level of similarity between treatment and control units at the baseline? (d) How much protection against contamination was present in the study? (e) How free was the study from selective reporting? (f) How free was the study from other reported risks of bias?

**Table 4:** Assessment of risk of bias in eligible hot spots policing studies

Study (author(s), year)	Random allocation^a^	Randomization process^b^	Selection^c^	Protection from contamination^d^	Nonreporting^e^	Other bias^f^
Sherman et al. (1989)	High	High	Low	High	High	High
Sviridoff et al. (1992)	Low	Low	High	High	High	High
Hope (1994)	Low	Low	Low	High	High	High
Sherman and Weisburd (1995)	High	High	High	High	High	High
Weisburd and Green (1995b)	High	High	High	High	High	High
Sherman and Rogan (1995a)	Low	Low	High	High	High	High
Sherman and Rogan (1995b)	High	High	High	High	High	High
Criminal Justice Commission (1998)	Low	Low	High	High	High	High
Braga et al. (1999)	High	High	Unclear	High	High	High
Caeti (1999)	Low	Low	High	High	High	Low
Mazerolle et al. (2000)	High	High	High	High	High	High
Cohen et al. (2003)	Low	Low	Low	High	High	High
DiTella and Schargrodsky 2004	Low	Low	High	High	High	High
Lawton et al. (2005)	Low	Low	High	High	High	High
Weisburd et al. (2006)	Low	Low	Low	High	High	High
Braga and Bond (2008)	High	High	Unclear	High	High	High
Beck (2010)	Low	Low	High	High	High	High
Berk and MacDonald (2010)	Low	Low	High	High	High	High
Braga et al. (2011)	Low	Low	High	High	High	High
Lum et al. (2011)	High	High	High	High	High	High
Ratcliffe and Breen (2011)	Low	Low	Low	High	High	High
Ratcliffe et al. (2011)	High	High	High	High	High	High
Taylor et al. (2011)	High	High	High	High	High	High
Fielding and Jones (2012)	Low	Low	Low	High	High	High
Weisburd et al. (2012)	High	High	High	High	High	High
Uchida and Swatt (2013)	Low	Low	Low	High	High	High
Martinez (2013)	Low	Low	Low	High	High	High
Koper et al. (2013)	High	High	High	High	High	High
Banerjee et al. (2014)	High	High	Unclear	High	High	High
Bond et al. (2014)	Low	Low	Low	High	High	High
Bryant et al. (2014)	Low	Low	Low	High	High	High
Marklund and Merenius (2014) (Eskilstuna)	Low	Low	Low	High	High	High
Marklund and Merenius (2014) (Stockholm)	Low	Low	Low	High	High	High
Mazeika (2014)	Low	Low	High	High	High	High
Piza and O'Hara (2014)	Low	Low	High	High	High	High
Rosenfeld et al. (2014)	High	High	High	High	High	High
Sherman et al. (2014)	High	High	High	High	High	High
Telep et al. (2014)	High	High	High	High	High	High
Groff et al. (2015)	High	High	High	High	High	High
Kennedy et al. (2015) (CO)	Low	Low	Unclear	High	High	High
Kennedy et al. (2015) (NJ)	Low	Low	Unclear	High	High	High
Kennedy et al. (2015) (MO)	Low	Low	Unclear	High	High	High
Kennedy et al. (2015) (AZ)	Low	Low	Unclear	Unclear	High	High
Kochel et al. (2015)	High	High	Unclear	High	High	High
Koper et al. (2015)	High	High	High	High	High	High
Marklund and Holmberg (2015)	Low	Low	Low	High	High	High
Piza et al. (2015)	High	High	High	High	High	High
Santos and Santos (2015a, 2015b)	Low	Low	High	High	High	High
Sorg (2015)	Low	Low	High	High	High	High
Weisburd et al. (2015)	High	High	Unclear	High	High	High
Williams (2015)	Unclear	Unclear	Unclear	Unclear	High	High
Ariel and Partridge (2017)	High	High	High	High	High	High
Ariel et al. (2016)	High	High	High	High	High	High
Dario (2016)	Low	Low	Low	High	High	High
Gerell (2016)	Low	Low	Low	High	High	High
MacDonald et al. (2016)	Low	Low	Low	High	High	High
Novak et al. (2016)	Low	Low	Unclear	High	High	High
Phillips et al. (2016)	Low	Low	High	High	High	High
Santos and Santos (2016)	High	High	High	High	High	High
Sedelmaier and Hipple (2016)	Low	Low	High	High	High	High
Attermann (2017)	High	High	High	High	High	High
Blattman et al. (2017)	High	High	High	Low	High	Low
Ratcliffe et al. (2017)	High	High	High	High	High	High
Rydberg et al. (2017)	Low	Low	High	Low	High	High
Wheeler and Phillips (2018)	Low	Low	High	High	High	High
“High” Totals	27	27	37	61	65	62
% of *N *= 65 studies	41.5%	41.5%	56.9%	93.8%	100%	95.4%

^a^ To what extent was the random allocation sequence adequately generated?

^b^ How well was the randomization sequence followed?

^c^ What was the level of similarity between treatment and control units at the baseline?

^d^ How much protection against contamination was present in the study?

^e^ How free was the study from selective reporting?

^f^ How free was the study from other reported risks of bias?

All 27 randomized controlled trials included in this review used credible methods for randomization and did not report any issue in the implementation of the randomization scheme implemented. However, there were some limitations to the internal validity of the included studies. More than half of all eligible studies (*N *= 37, 56.9%) provided direct evidence (usually in the form of a table that presented balanced outcomes and descriptive variables) that the treatment and control units were similar at the baseline measurement period. Another 11 studies (16.9%) provided descriptions of methods, such as block randomization (e.g., Braga et al., 1999) and propensity score matching (e.g., Kennedy et al., 2015), that create balanced treatment and control groups but did not provide clear evidence that the described techniques actually achieved balance. Seventeen studies (26.2%) used treatment and control units that were not the same. For instance, the Jersey City Displacement and Diffusion Study compared crime outcomes in the targeted areas relative to crime outcomes in the rest of the city. The simple randomization procedure used in the Minneapolis RECAP experiment led to the placement of some of the highest event addresses into the treatment group; this led to high variability between the treatment and control groups and low statistical power (Sherman et al., 1989).

Sixty‐one studies (93.8%) did not report any evidence of contamination of control conditions during the intervention period. Four studies either explicitly noted possible contamination or presented indirect evidence that contamination was very likely. For instance, the adjacency of included experimental segments in a map presenting hot spot locations in the Bogota hot spots policing experiment was highly suggestive of contamination effects (Blattman et al., 2017, p. 8). None of the included studies reported evidence suggestive that the evaluators were only selecting those crime types that showed an effect. Finally, only three studies (4.6%) presented any other evidence of possible bias. For example, the Bogota hot spots policing study reported crime outcome measures that confounded violent crime (home robbery; person robbery) with property crime (no burglary, breaking/entering, or theft from person measures included) and did not include larcenies from a person (Blattman et al., 2017, p. 12).

The internal validity of the included studies was generally high. There were variations in the overall strength of the research designs used by included studies: 27 studies used randomized controlled trials and 38 studies used quasiexperimental designs. Among the 38 studies that utilized a quasiexperimental approach, the strength of the research design varied. Therefore, we conducted sensitivity analyses that tested the moderating effects of research design on the relationship between hot spots policing programs and crime outcomes.

### 5.5 Meta‐analysis of the effects of hot spots policing on crime

Our meta‐analyses of the effects of hot spots policing programs on crime were limited to 62 of the 65 eligible studies. Two studies, the St. Louis Problem‐Oriented Policing in Three Drug Market Locations Study (Hope, 1994) and the Beenleigh (Australia) Calls for Service Project (Criminal Justice Commission, 1998), did not report the necessary information to calculate program effect sizes. As described in Appendix D, the Houston (TX) Targeted Beat Program (Caeti, 1999) did not use appropriate statistical methods to estimate program effects and, unfortunately, accurate effect sizes could not be calculated. We were able to calculate effect sizes for 73 main effects tests and 40 displacement and diffusion tests in these 65 eligible studies. As such, the unit of analysis in the meta‐analyses presented here represent these independent tests rather than individual studies.

**Figure 2:**
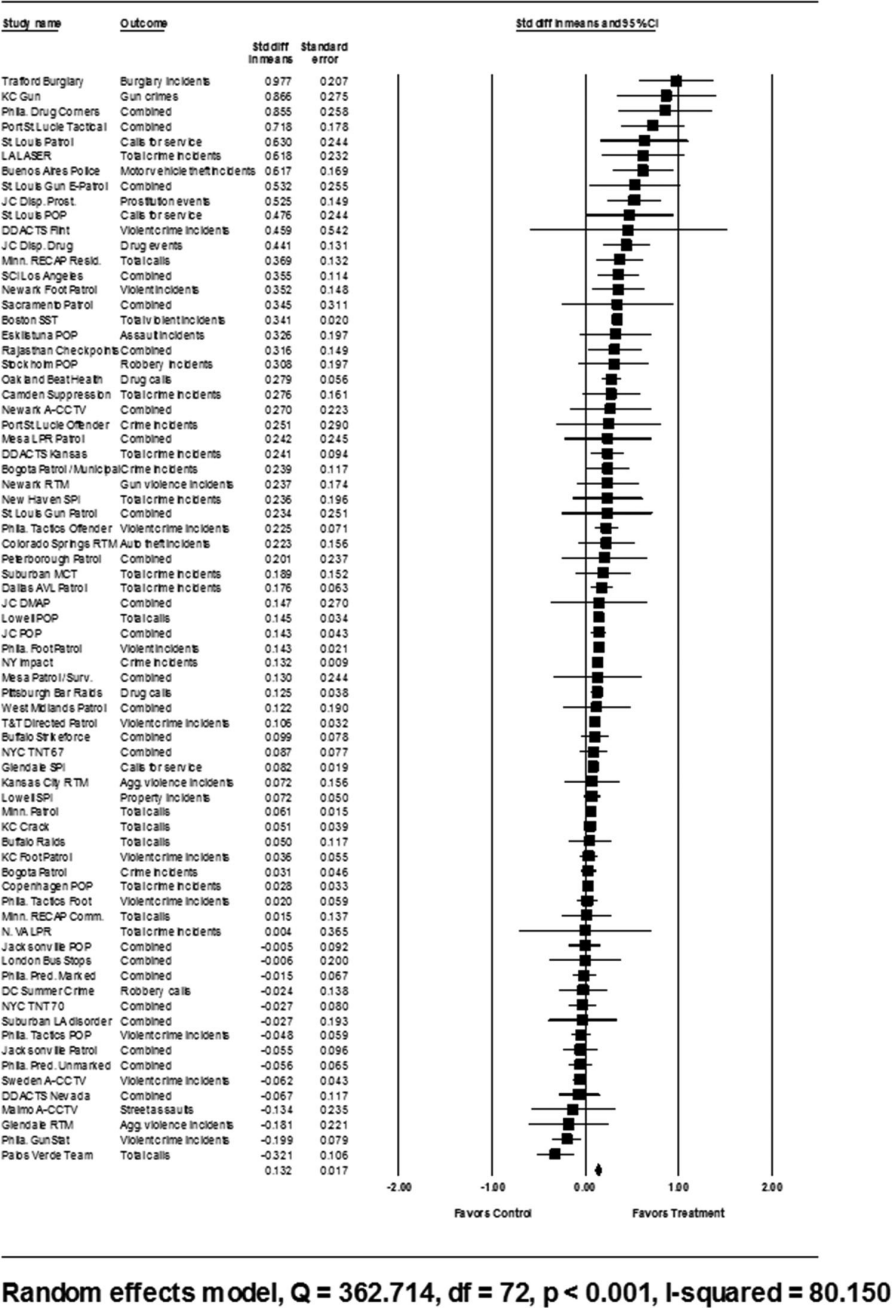
Combined effect sizes for study outcomes

Using the overall mean effect size from each study for 73 main effects tests, the forest plot in Figure 2 show the standardized difference in means between the treatment and control or comparison conditions (effect size) with a 95% confidence interval (CI) plotted around them for all tests. Points plotted to the right of 0 indicate a treatment effect; in this case, the test showed a reduction in crime or disorder. Points to the left of 0 indicate a backfire effect where control conditions improved relative to treatment conditions. A random‐effects model was used to estimate the overall mean effect size based on an a priori assumption of a heterogeneous distribution of effect sizes.^8^ The meta‐analysis of effect sizes suggests an effect in favor of hot spots policing strategies. Notably, the overall effect size for these studies is 0.132 (*p* < .001); this would be considered a small mean effect size (see Cohen, 1988).

**Figure 3:**
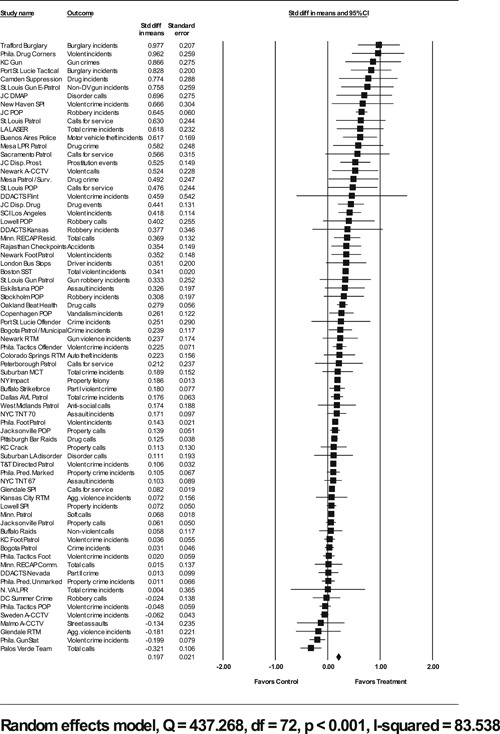
Largest effect sizes for study outcomes

**Figure 4:**
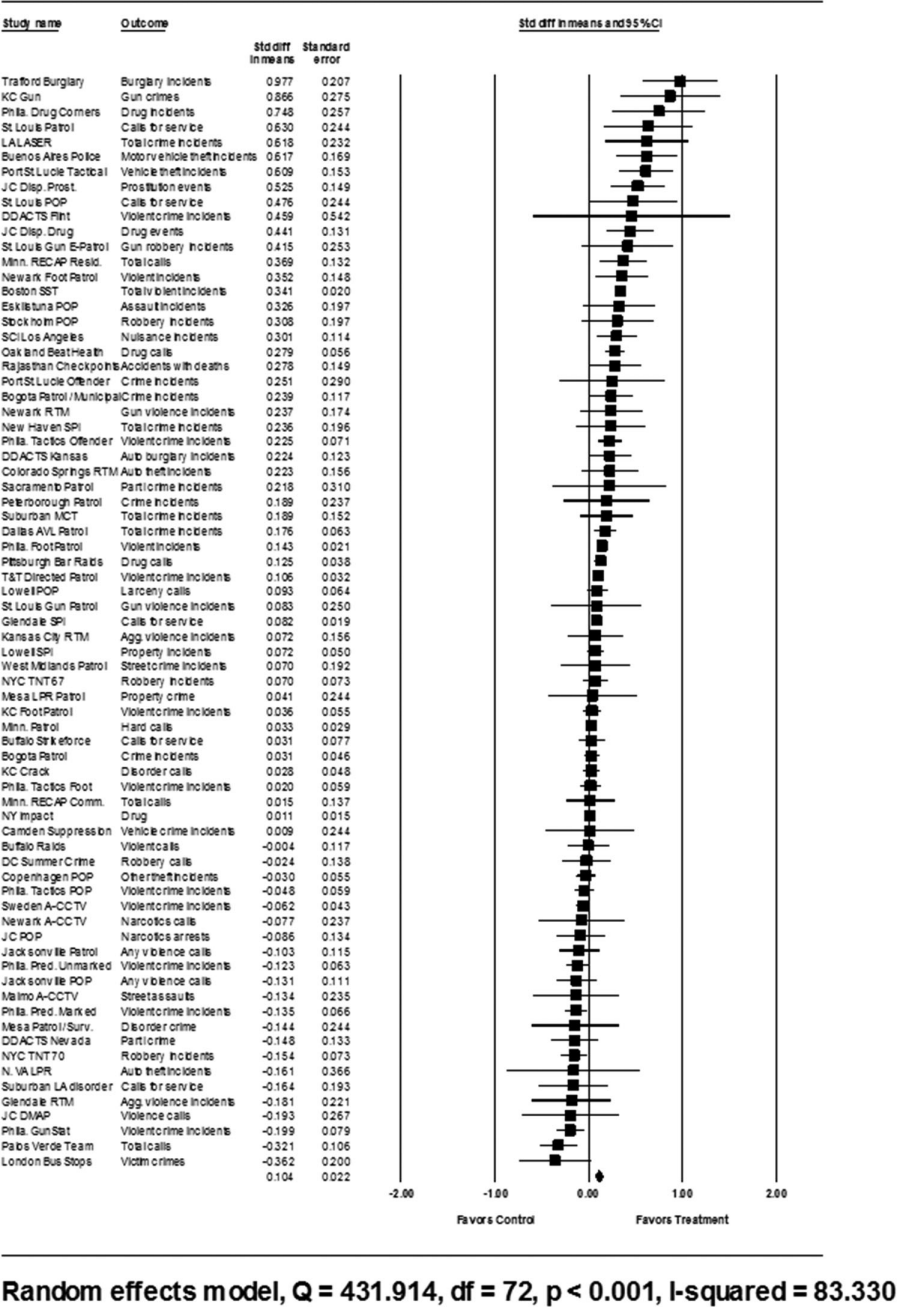
Smallest effect sizes for study outcomes

Fifty‐seven tests reported effect sizes that favored treatment conditions over control conditions (78.1% of 73 total tests). The Trafford (UK) Predictive Risk Mapping and Policing quasiexperiment (0.977), Kansas City Gun quasiexperiment (0.866), and Philadelphia Drug Corners Crackdown quasiexperiment (0.855) tests reported the largest statistically significant effect sizes while the Minneapolis Hot Spots Patrol experiment (0.061) reported the smallest statistically significant effect size. The forest plots in Figures 3 and 4 present the meta‐analyses of the largest and smallest effect sizes for each study, respectively.^9^ For the largest effect size meta‐analysis, the overall standardized mean difference effect size was 0.197 and statistically significant at the *p* < .05 level. For the smallest effect size meta‐analysis, the overall standardized mean difference effect size was 0.104 and statistically significant at the *p* < .05 level. Table 5 presents mean effect sizes for the effects of hot spots policing programs disaggregated by crime type. Hot spots policing programs produced statistically significant (*p* < .05) positive mean effect sizes for violent crime outcomes (0.102), property crime outcomes (0.124), disorder outcomes (0.161), and drug crime outcomes (0.244).

**Table 5:** The effects of hot spots policing on specific crime types

Crime category	*N* Studies	Effect size
Violent crimes	44	0.102^*^
Property crimes	26	0.124^*^
Disorder offenses	15	0.161^*^
Drug offenses	10	0.244^*^

*Note:* Random effects meta‐analysis models used in all reported effect sizes.

^*^ *p* < .05.

**Figure 5:**
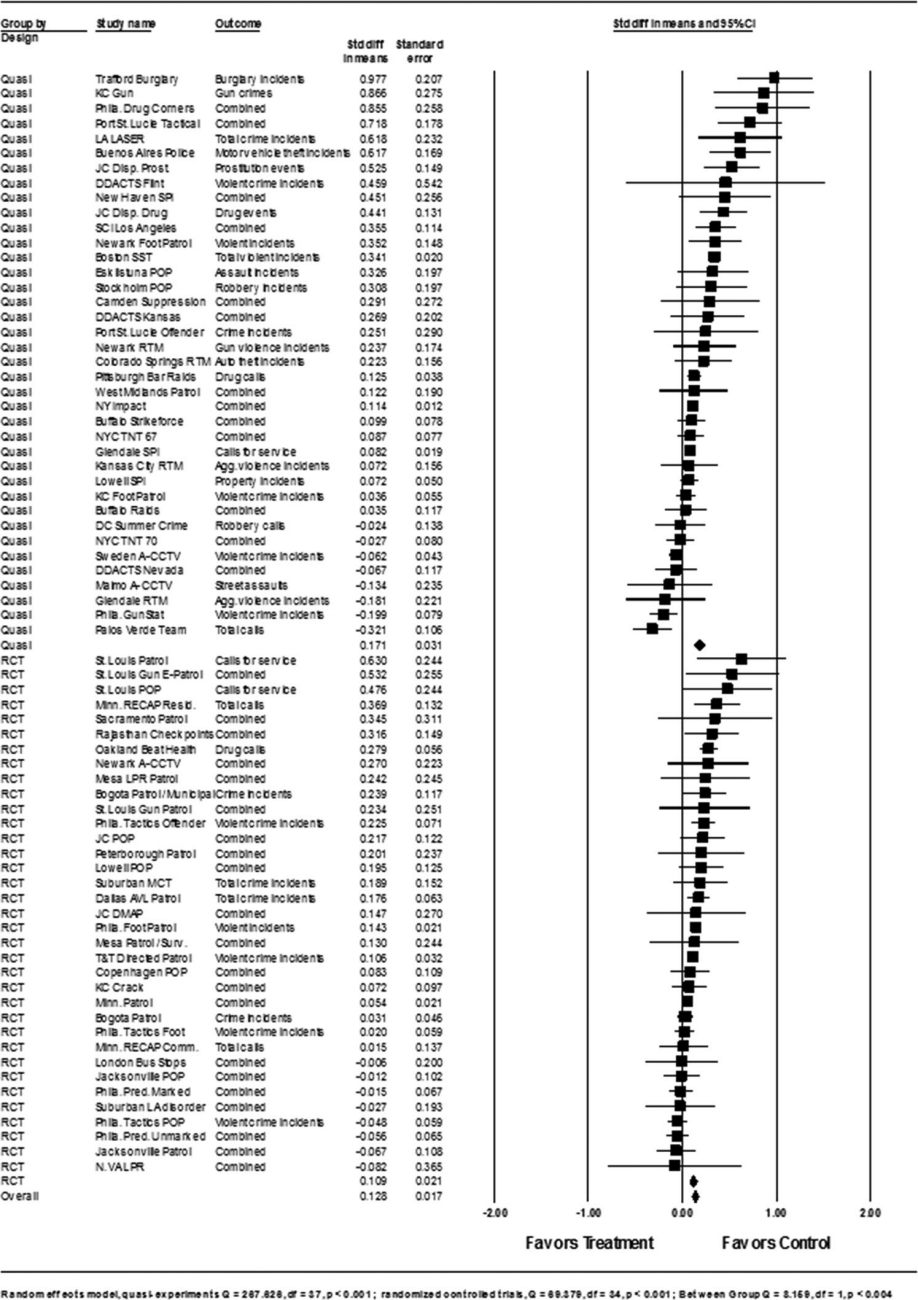
Research design type as moderator for study outcomes

Given the important distinction in methodological quality between the randomized controlled trials and quasiexperimental evaluation studies, we also explored research design as a moderator variable. It is well known among social scientists that program evaluations with more rigorous research designs tend to report null effects compared to evaluations with weaker research designs. As Rossi's (1987) Iron Law of Evaluation states, “The expected value of any net impact assessment of any large scale social program is zero” (p. 3). And as his Stainless Steel Law of Evaluation posits, “The better designed the impact assessment of a social program, the more likely is the resulting estimate of net impact to be zero” (Rossi 1987, p. 3). Figure 5 presents a random‐effects model that considers the two different classes of evaluation designs included in this review. The quasiexperimental designs were associated with a modestly larger within‐group effect size (0.171, *p* < .001) relative to the randomized controlled trial designs (0.109, *p* < .001).^10^ We also conducted an exploratory moderator analysis that suggested stronger quasiexperimental designs produced a slightly more conservative effect size estimate (0.158, *p* < .001) relative to weaker quasiexperimental designs (0.188, *p* < .001) but these differences were not statistically significant (between group *Q* = 0.194, df = 1, *p* = .660).^11^

#### 5.5.1 Meta‐analysis of displacement and diffusion effects

Prior to a discussion of the research findings, it must be noted that it is very difficult to detect displacement effects because the potential manifestations of displacement are quite diverse. As Barr and Pease (1990) suggest, “if, in truth, displacement is complete, some displaced crime will fall outside the areas and types of crime being studied or be so dispersed as to be masked by background variation…no research study, however massive, is likely to resolve the issue” (p. 293). The same difficulties are encountered when testing for diffusion effects. Most tests were limited to examining immediate spatial displacement and diffusion effects; that is, whether focused police efforts in targeted areas resulted in crime “moving around the corner” or whether these proximate areas experienced unintended crime control benefits.

**Figure 6:**
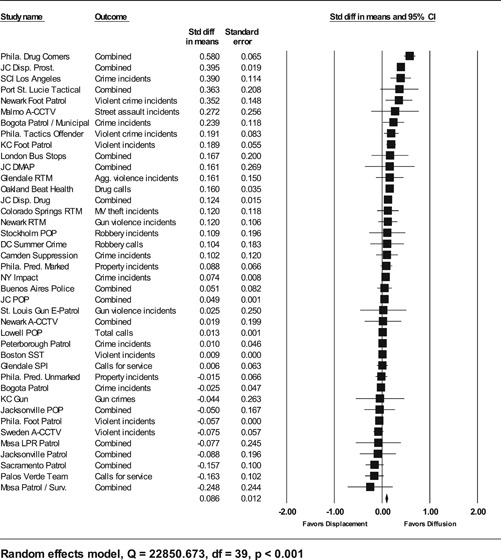
Combined effect sizes for displacement and diffusion outcomes

In this analysis, we analyzed immediate crime displacement and diffusion effects jointly as two sides of a single distribution that ranged from harmful to beneficial effects in areas adjacent to the treatment and control hot spots. Using the overall mean effect size from each study for 40 displacement and diffusion tests, the forest plots in Figure 6 show the standardized difference in means between the treatment and control or comparison conditions (effect size) with a 95% CI plotted around them for all tests. Points plotted to the right of 0 indicate a diffusion of crime control benefits effect; in this case, the test showed a reduction in crime or disorder in the areas surrounding the targeted hot spots. Points to the left of 0 indicate a crime displacement effect. We used a random‐effects model to estimate the overall mean effect size.^12^ The meta‐analysis suggests a small but statistically significant overall diffusion of crime control benefits effect (0.086) generated by the hot spots policing strategies (*p *< .001).

**Figure 7:**
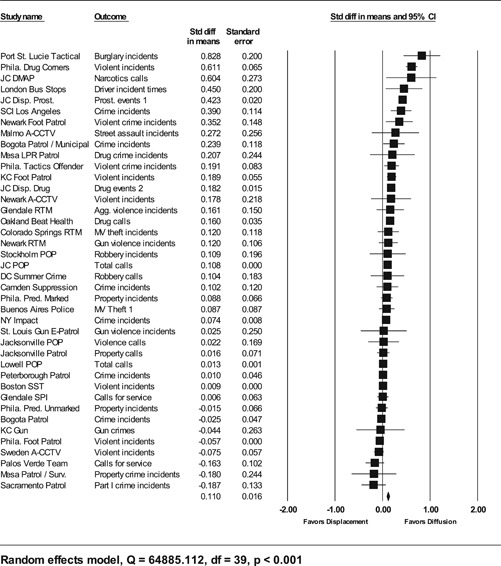
Largest effect sizes for displacement and diffusion outcomes

**Figure 8:**
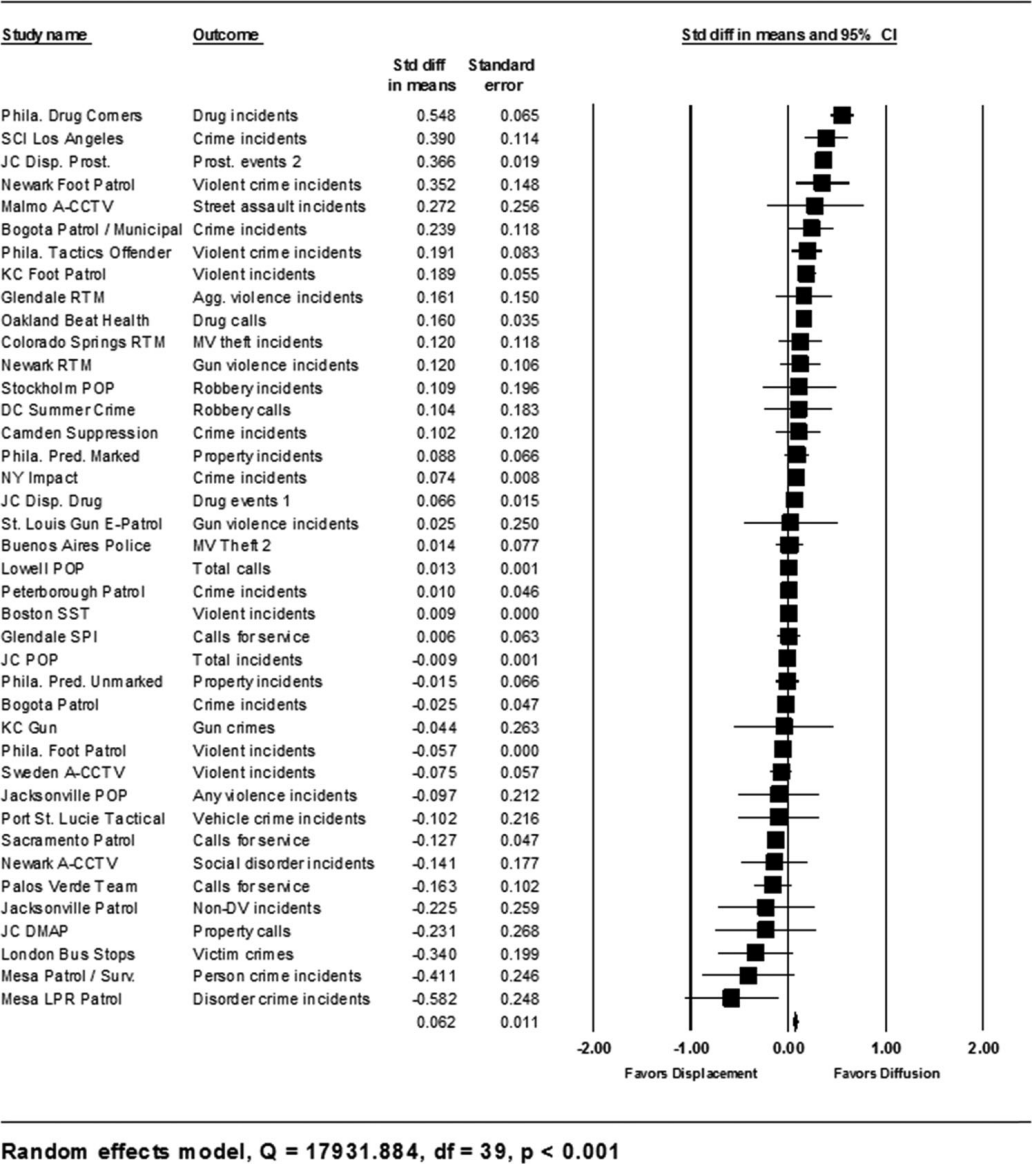
Smallest effect sizes for displacement and diffusion outcomes

Twenty‐nine tests (72.5% of 40 total tests) reported effect sizes that favored diffusion effects over displacement effects. The largest statistically significant diffusion effects were reported by the Philadelphia Drug Corners Crackdown quasiexperiment (0.580), Jersey City Displacement and Diffusion Study quasiexperiments (buffers around prostitution site = 0.395, buffers around drug crime site = 0.124),^13^ and the Los Angeles Safer Cities Initiative quasiexperiment (0.390). Eleven tests (27.5% of 40 total tests) reported effect sizes that favored displacement effects over diffusion effects. The Philadelphia Foot Patrol experiment was the only study that reported a statistically significant displacement effect (−0.057). The forest plots in Figures 7 and 8 present the meta‐analyses of the largest and smallest effect sizes for each study, respectively.^14^ Both meta‐analyses estimated overall effect sizes that favored diffusion effects over displacement effects. For the largest effect size meta‐analysis, the overall standardized mean difference effect size was small (0.110) and statistically significant at the *p* < .05 level. For the smallest effect size meta‐analysis, the overall standardized mean difference effect size was also small (0.062) but still statistically significant at the *p* < .05 level.

#### 5.5.2 Program type as effect size moderator

Our narrative review documented that hot spots policing programs have adopted POP, focused drug enforcement, increased patrol, increased gun searches and seizures, and zero‐tolerance policing to control high‐activity crime places. POP programs attempt to change the underlying conditions at hot spots that cause them to generate recurring crime problems (Braga & Weisburd, 2010; Goldstein, 1990). The other hot spots policing interventions represent increased traditional policing activities concentrated at specific places to prevent crime through general deterrence and increased risk of apprehension (Nagin et al., 2015). There is, of course, some overlap between the enforcement interventions employed by the POP hot spots programs and the actions taken by the increased policing hot spots programs. However, these two general types of programs represent fundamentally different orientations in dealing with the problems of high‐activity crime places.

**Figure 9:**
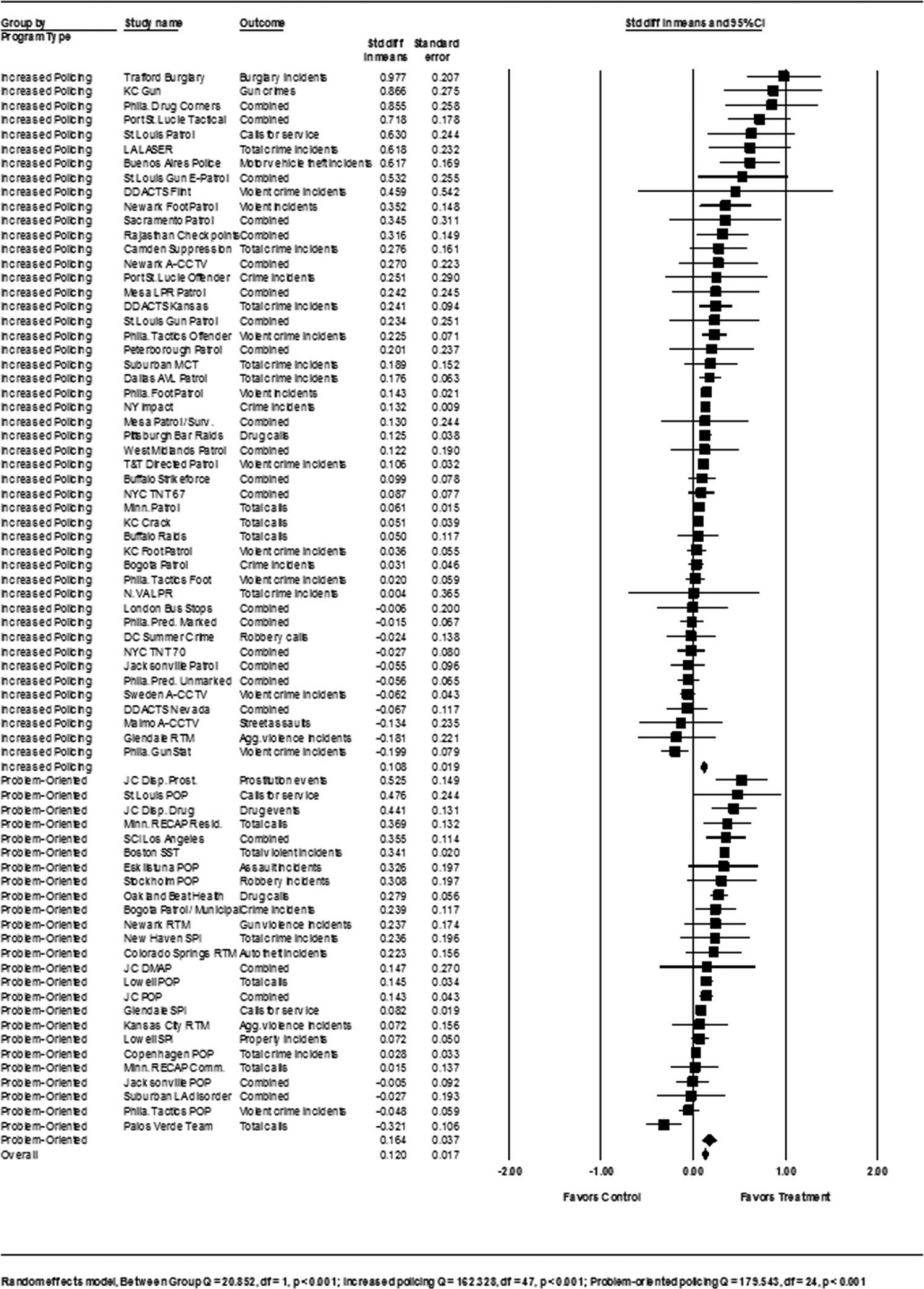
Hot spots policing program type as moderator for study outcomes

Moderator variables help to explain and understand differences across studies in the outcomes observed. Program type could be an influential moderator of the observed effect sizes in our overall meta‐analysis. Figure 9 presents a random‐effects model examining the two different hot spots policing program types: POP and increased policing.^15^ Our meta‐analysis revealed that POP programs produced a modestly larger overall mean effect size (0.164, *p* < .001) relative to the size of the overall mean effect size generated by increased traditional policing programs (0.108, *p* < .001).

#### 5.5.3 Publication bias

Publication bias, generally defined as the concern that the collection of studies easily available to a reviewer represents those studies most likely to have statistically significant results, presents a strong challenge to any review of evaluation studies (Rothstein, 2008). The credibility of a review arguably depends more heavily on the collection of studies reviewed than on which statistical methods of synthesis are used (Wilson, 2009). Similar to the problem of a biased study sample leading to biased results in an individual study, a biased collection of studies will potentially lead to biased conclusions in a systematic review (Rothstein & Hopewell, 2009). As reported earlier, our search strategies were designed to mitigate the potential effects of publication bias on our analyses. Indeed, it is encouraging that nearly half of the eligible studies (29 of 65, 44.6%) were acquired through gray literature sources such as published reports, theses, dissertations, unpublished reports, and unpublished working papers. The studies identified through gray literature sources reported a much smaller overall mean effect size (0.060, *p *< .001) when compared with the overall mean effect size (0.200, *p *< .001) reported by studies in published journal articles, suggesting that our search strategies were successful in identifying a range of hot spots policing studies with varying effects on crime outcomes.^16^

Like many systematic reviews, our meta‐analyses used the trim‐and‐fill procedure to explore whether publication bias might be affecting the results and to estimate how the reported effects would change if the bias were to be removed (Duval & Tweedie, 2000; Duval, 2005). The diagnostic funnel plot is based on the idea that, in the absence of bias, the plot of study effect sizes should be symmetric about the mean effect size. If there is asymmetry, the trim‐and‐fill procedure imputes the missing studies, adds them to the analysis, and then recomputes the mean effect size. Trim‐and‐fill procedures do suffer from some well‐known limitations that could result in the underestimation or overestimation of publication bias (Rothstein, 2008; Simonsohn, Nelson, & Simmons, 2014).^17^ Nonetheless, this approach does provide reviewers with a well‐understood measure of the possible influence of bias on their meta‐analytic results.

**Figure 10:**
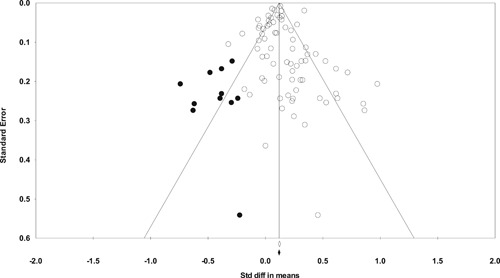
Funnel plot of standard error by standardized difference in means. Empty circles are the original studies. Filled‐in circles indicate 11 imputed studies from the trim‐and‐fill analysis. These additional studies only slightly changed the mean effect size estimate. Using a random effects model, decreased from 0.132 (95% CI = 0.097, 0.165) to 0.103 (95% CI = 0.067, 0.138). CI, confidence interval

A visual inspection of the resulting funnel plot indicated some asymmetry with more studies with a large effect and a large standard error to the right of the mean than the left of the mean. The trim‐and‐fill procedure determined that 11 studies should be added to create symmetry. The funnel plot with imputed studies is presented in Figure 10. Using a random‐effects model, the mean random effect decreased from 0.132 (95% CI = 0.097, 0.165) to 0.103 (95% CI = 0.067, 0.138). Indeed, the 95% CIs substantially overlap, suggesting that the underlying parameters may not be different. Nevertheless, the trim‐and‐fill result suggests mild publication selection bias. However, the adjusted mean effect size remained a similar statistically significant small size and, as such, the observed publication bias does not appear to be sufficient to nullify the results (as suggested by the funnel plot in Figure 10).

## 6 DISCUSSION

### 6.1 Summary of main results

Overall, results from this review suggest that hot spots policing is associated with small but meaningful crime control gains. The preventive impact of hot spots policing was statistically significant for crime overall and when crime outcomes were disaggregated by offense type. Programs that focused police resources and attention on high‐activity small crime places concentrated generated reductions in drug offenses, disorder offenses, property crimes, and violent crimes.

Slightly more than half of the 78 tests of hot spots policing examined potential crime displacement and diffusion effects. Narrative reviews of these studies indicated little evidence of crime displacement; indeed, the studies suggested hot spots policing was more likely to produce unintended crime prevention benefits in areas immediately adjacent to targeted hot spots. Additionally, a meta‐analysis of key reported outcome measures suggest hot spots policing has a small but statistically significant overall mean effect size in favor of a diffusion of crime control benefits over crime displacement effects.

There was some evidence that the research design used in the included studies moderated the magnitude of the impact of hot spots policing on crime. The within‐group effect size for quasiexperimental designs was somewhat larger when compared with randomized controlled trial designs. Nevertheless, the effects of hot spots policing on crime remained statistically significant regardless of the research design. Among studies that used quasiexperimental designs, studies that utilized more rigorous designs showed slightly more conservative effect size estimates compared with studies with weaker designs. However, the within‐group effect size differences between stronger and weaker quasiexperiments were not statistically significant.

The magnitude of the impact of hot spots policing also varied by program type. Hot spots policing initiatives that used POP interventions generated a modestly larger overall mean effect size relative to the overall mean effect size generated by increased traditional policing programs.

### 6.2 Overall completeness and applicability of evidence

Positive findings produced in this review have widespread applicability to the field of policing and crime prevention. The previous iteration of this review contained 19 studies dating back to 1989. This updated review identified 46 eligible studies published between 2010 and February 2017 for a new total of 65 eligible studies. With the addition of a large number of hot spots policing studies, the essential finding of this review was reaffirmed: hot spots policing generates small reductions in crime. Most eligible hot spots policing interventions occurred in the United States (51 studies); however, 12 studies were implemented in other countries thereby suggesting a general applicability of hot spots policing across varying contexts. Only one study included in the review conducted a formal cost‐benefit analysis. Therefore, further research is warranted on the cost‐effectiveness of hot spots policing to traditional policing strategies.

### 6.3 Quality of the evidence

The overall quality of evidence present in this review is robust. Randomized controlled trial designs were used in almost half of eligible studies and among the quasiexperimental studies, many used rigorous evaluation methods. Positive crime control findings were observed for both experimental and quasiexperimental research designs. More than half of eligible studies demonstrated that treatment and control units were similar at the baseline measurement period. There was no evidence that authors of eligible studies engaged in selective reporting of crime outcomes. Furthermore, evidence of contamination of treatment was absent in nearly all of the eligible studies.

### 6.4 Limitations and potential biases in the review process

Outcome measured by studies included in this review relied exclusively on official records and did not include measures of self‐report victimization. This review was also unable to calculate standardized effect sizes for three studies containing five tests of hot spots policing due to the insufficient or inadequate information being presented.

### 6.5 Agreements and disagreements with other studies or reviews

The results of this systematic review support the assertion that focusing police efforts at high activity crime places can be effective in preventing crime (Skogan & Frydl, 2004; Weisburd & Majmundar, 2018). This review reaffirms and strengthens results on the effectiveness of hot spots policing at reducing crime from previous iterations of systematic review and meta‐analysis of hot spots policing (Braga et al., 2012, 2014; Braga, 2001, 2005, 2007). Our findings on hot spots policing rarely generating crime displacement and more likely producing a diffusion of crime control benefits into adjacent areas is consistent with findings from prior reviews (Bowers et al., 2011; Weisburd & Majmundar, 2018), but are contrary to arguments made in other works (Blattman et al., 2017; Reppetto, 1976).

## 7 AUTHORS' CONCLUSIONS

### 7.1 Implications for practice and policy

Evidence from this review suggests hot spots policing is an effective approach to crime prevention. However, police executives and policymakers should note certain practices may generate stronger impacts at high‐crime places. In our review, we found that POP interventions generated larger overall effect sizes when compared with the increased policing interventions. While increasing presence and concentrating traditional enforcement activities constitute an effective police response to crime hot spots, it seems likely that altering place characteristics and dynamics will produce larger crime prevention benefits (Braga & Weisburd, 2010). We believe that the POP approach holds great promise in developing tailored responses to very specific recurring problems at crime hot spots. While it is difficult for police agencies to implement the “ideal” version of POP (Braga & Weisburd, 2006; Cordner & Biebel, 2005; Eck, 2006), the available evidence suggests that even “shallow” problem solving better focuses police crime prevention efforts at crime hot spots.

Proactive policing strategies, such as hot spots policing programs, have been suggested to lead to abusive and unlawful policing practices in disadvantaged minority neighborhoods (Tso, 2016). Indeed, Rosenbaum (2006) cautions that hot spots policing can easily become zero‐tolerance and indiscriminate aggressive tactics can drive a wedge between the police and communities. An evaluation of the adverse system side effects of Operation Sunrise, described here as the Philadelphia Drug Corners Crackdown, found that initiative strained the local judicial system by generated a high volume of arrests that resulted in a significant increase in fugitive defendants (Goldkamp & Vilcica, 2008). Short‐term crime gains produced by particular types of hot spots policing initiatives could undermine the long‐term stability of specific neighborhoods through the increased involvement of mostly low‐income minority men in the criminal justice system.

Only seven studies included in this review examined the impacts of hot spots policing on community residents. These studies found little evidence that hot spots policing programs result have negative impacts on police‐relations. A recent report by the U.S. National Academies Committee on Proactive Policing supports this position, noting that proactive policing strategies such as hot spots policing show “consistent evidence of [crime reduction] effectiveness without evidence of negative community outcomes” (Weisburd & Majmundar 2018, p. 13). However, the committee also recognized the scant evidence on this issue and acknowledged that the potential impacts of hot spots policing on legitimacy may depend in good part on the types of strategies used and the context of the hot spots affected. Implementing problem‐oriented and situational prevention strategies that reduce police reliance on aggressive enforcement strategies in crime hot spots may not only generate stronger crime control gains but could also yield positive benefits for police–community relations. Whatever the impact, we clearly need to know more about the effects of hot spots policing approaches on the communities that the police serve.

Finally, in closing, we were surprised that only one of the 65 hot spots policing evaluations reviewed here conducted formal cost‐benefit assessments. Operation Style in Peterborough, England found that 21 more minutes of uniformed and unarmed patrol by Police Community Support Officers (PCSO) was linked to 85 to 360 fewer potential days of imprisonment in each targeted hot spot relative to control areas. This imprisonment reduction was associated with 5.6–23 Euros saved for every 1 Euro spent on PCSO patrol, or $6.68–$27.45 USD per $1.19 USD spent on PCSO patrol (Ariel et al., 2016). It is unfortunately rare for crime and justice program evaluations to include analyses of monetary costs of running the program relative to the benefits accrued by preventing crimes (Welsh & Farrington, 2000). When monetary costs were explicitly mentioned in the hot spots policing evaluations, it was usually to acknowledge that additional patrols in hot spot areas were supported by the police department's own overtime budget (e.g., Taylor et al., 2011) or through external grant funds (e.g., Sherman & Rogan, 1995a). Many of the evaluations implied that the hot spots interventions were supported via reallocating existing resources into the treatment areas without incurring any additional costs. Nevertheless, the policy impact of this body of research would be considerably strengthened if evaluation demonstrated that hot spots policing programs generated both crime control gains and monetary savings relative to traditional policing methods.

### 7.2 Implications for research

Our systematic review identified 78 tests of hot spots policing in 65 eligible studies. Sixty‐two of the 78 tests reported noteworthy crime control gains associated with the hot spots policing interventions when treatment conditions were compared to control conditions. A meta‐analysis of key reported outcome measures revealed a small but statistically significant mean effect size favoring the effects of hot spots policing in reducing crime in treatment places relative to control places. When crime displacement was measured, it was very limited and unintended crime prevention benefits were more likely to be associated with the hot spots policing programs (see also Bowers et al., 2011). A meta‐analysis of key reported outcome measures in 40 tests revealed a small but statistically significant mean effect size favoring a diffusion of crime control benefits rather than a crime displacement effect.

Twenty‐seven of the 65 eligible studies in this review used randomized controlled trials to evaluate the effects of hot spots policing on crime. When research design was considered as an effect size moderator, our meta‐analysis reported that the quasiexperimental evaluation generated larger overall effect sizes when compared with the randomized controlled trials. While the biases in quasiexperimental research are not clear (e.g., Campbell & Boruch, 1975; Wilkinson & Task Force on Statistical Inference, 1999), two recent reviews in crime and justice suggest that weaker research designs might lead to more positive outcomes (e.g., see Weisburd, Lum, & Petrosino, 2001; Welsh, Peel, Farrington, Elffers, & Braga, 2011). This does not mean that nonexperimental studies cannot be of high quality, but only that there is evidence that nonexperimental designs in hot spots policing evaluations seem likely to overstate outcomes as contrasted with randomized experiments. However, the purported relationship between quasiexperimental designs and larger effect sizes has not been universally found (e.g., see Lipsey & Wilson, 2001; Shadish & Ragsdale, 1996).

## 8 SOURCES OF SUPPORT

Earlier iterations of this systematic review were supported in part by funds from the Smith Richardson Foundation and the U.S. National Academy of Sciences.

## 9 DECLARATIONS OF INTEREST

With colleagues, Braga has conducted two randomized controlled experiments and one quasiexperimental evaluation that found hot spots policing to be effective in controlling crime and disorder problems. Moreover, his colleagues (e.g., David Weisburd and Lorraine Mazerolle) have conducted other experimental evaluations of the effects of hot spots policing on crime. Although Braga does not have an ideological bias toward the effectiveness of place‐focused interventions, it may be uncomfortable for him to report findings in this review that contradict the findings of his experiment or experiments conducted by his colleagues. Papachristos and Hureau have collaborated with Braga on an evaluation of the effects of hot spots policing program in Boston. Beyond that single study, neither Papachristos nor Hureau has been involved in evaluating hot spots policing interventions. Turchan has not been involved in any hot spots policing evaluations.

## 10 ROLES AND RESPONSIBILITIES

A. A. B. designed the original systematic review following established Campbell Collaboration conventions and protocols; A. A. B., D. M. H., and A. V. P. designed the second iteration while A. A. B., B. T., D. M. H., and A. V. P. designed the third iteration. With the assistance of Phyllis Schultze, B. T., and A. A. B. executed the varied search strategies to identify eligible studies. A. A. B., B. T., D. M. H., and A. V. P. selected eligible studies that fit the established criteria and coded the characteristics of the eligible studies. A. A. B., B. T., D. M. H., and A. V. P. calculated standardized mean effect sizes and executed the formal meta‐analyses. B. T. and A. A. B. wrote the narrative reviews for each eligible study. A. A. B., B. T., D. M. H., and A. V. P. collaborated closely on the writing of the literature review, methodology and analysis sections, results, and conclusion.

- Content: A. A. B., B. T., A. V. P., and D. M. H.
- Systematic review methods: A. A. B., B. T., A. V. P., and D. M. H.
- Statistical analysis: A. A. B., B. T., A. V. P., and D. M. H.
- Information retrieval: A. A. B., and B. T.

## 11 SOURCES OF SUPPORT

Earlier iterations of this systematic review were supported in part by funds from the Smith Richardson Foundation and the U.S. National Academy of Sciences.

David B. Wilson deserves special thanks for his analytic support (and patience) in the completing the meta‐analysis. We would also like to thank Phyllis Schultze of Rutgers University's Criminal Justice Library, Rosalyn Bocker, and Deborah Braga for their assistance in searching for and locating eligible studies. David Weisburd, Larry Sherman, Mark Lipsey, Anthony Petrosino, Brandon Welsh, Charlotte Gill, Cynthia Lum, and David Farrington also deserve thanks for making helpful comments on earlier iterations of this review. Finally, we would like to thank David Weisburd, Josh Hinkle, and Cody Telep for sharing data from their POP systematic review and Bruce Taylor, Christopher Koper, and Daniel Woods for sharing data from their hot spots policing randomized controlled trial.

## 12 PLANS FOR UPDATING THE REVIEW

Anthony Braga will coordinate the next update to this review, with contributions from Brandon Turchan, Andrew Papachristos, and David Hureau. We plan to update this review every 5 years in accordance with Campbell Collaboration guidelines.

## Footnotes

1 These journals were: *Criminology, Criminology and Public Policy*, *Justice Quarterly*, *Journal of Research in Crime and Delinquency*, *Journal of Criminal Justice*, *Police Quarterly*, *Policing*, *Police Practice and Research*, *British Journal of Criminology*, *Journal of Quantitative Criminology*, *Crime and Delinquency*, *Journal of Criminal Law and Criminology*, *and Policing and Society*. Hand searches covered 1979–2017.

2 Ms. Phyllis Schultze of the Gottfredson Library at the Rutgers University School of Criminal Justice executed the initial abstract search and was consulted throughout on our search strategies. Ms. Schultze also helped identify comparable substitutes for abstract databases and indexes used in previous iterations of the review but were no longer maintained.

3 C2 SPECTR was searched in previous iterations of this review. However, this register has not been updated consistently by C2 and, as such, was not searched in this update of the hot spots policing review.

4
https://www.campbellcollaboration.org/effect‐size‐calculato.html

5 Overlapping search results is an issue that is frequently encountered when conducting a comprehensive exploration of research literature.

6 These activities included roadblocks, patrol with license plate reader technology, zero‐tolerance policing, and increased gun searches and seizures.

7 The landmark Kansas City Preventive Patrol Experiment had to be stopped and restarted three times before it was implemented properly; the patrol officers did not respect the boundaries of the treatment and control areas (Kelling, Pate, Dickman, & Brown, 1974). Likewise, the design of the Minneapolis Spouse Abuse Experiment was modified to a quasiexperiment when randomization could not be achieved because officers chose to arrest certain offenders on a nonrandom basis (Berk, Smyth, & Sherman, 1988).

8 For the overall main effects meta‐analysis, *Q* = 362.714, df = 72, *p* < .001 and *I* ^2^ = 80.150.

9 Random effects models were used to estimate the overall standardized mean effect sizes. For the largest effect size meta‐analysis, *Q* = 437.268, df = 72, *p* < .001, *I* ^2^ = 83.534. For the smallest effect size meta‐analysis, *Q* = 431.914, df = 72, *p* < .001, *I* ^2^ = 83.330.

10 We used a random‐effects model for this comparison. For the quasiexperiments, *Q* = 267.626, df = 37, *p* < .001, *I* ^2^ = 86.175. For the randomized controlled trials, *Q* = 69.379, df = 34, *p* < .001, *I* ^2^ = 50.994. For the overall analysis, the between group *Q* = 8.159, df = 1, *p* < .004, suggesting that the type of evaluation produced statistically significant differences in observed crime outcomes. The moderated overall effect size was 0.128 (standard error = 0.017, *p *< .001, 95% CI = 0.094, 0.162).

11 In this exploratory analysis, we first used the Maryland Scientific Methods Scale (Sherman et al., 1997) to distinguish between “Level 3” and “Level 4” (in a five‐level scale) quasiexperimental evaluations. Level 3 designs rule out many threats to internal validity such as history, maturation/trends, instrumentation, testing, and mortality. However, as Farrington et al. (2002) observe, the main problems of Level 3 evaluations center on selection effects and regression to the mean due to the nonequivalence of treatment and control conditions. Level 4 evaluations measure outcomes before and after the program in multiple treatment and control condition units. These types of designs have better statistical control of extraneous influences on the outcome and, relative to lower‐level evaluations, deal with selection and regression threats more adequately. We then further distinguished “strong” quasiexperimental evaluations by their use of small hot spot locations (e.g., street segments, clusters of addresses, etc.) as units of analysis rather than larger areal units (e.g., census block groups, precincts, etc.). When hot spot treatment effects are measured at larger areal units rather than at the actual treated hot spot locations (see, e.g., Sviridoff et al., 1992), the evaluations may not be well‐positioned to detect program effects if these impacts in fact exist.

12 Random effects models were used to estimate the overall displacement and diffusion standardized mean effect sizes: *Q* = 22850.673, df = 39, *p* < .001, *I* ^2^ =99.829.

13 The Jersey City Displacement and Diffusion Study quasiexperiment measured separate displacement and diffusion effects for one‐ and two‐block buffer zones surrounding the targeted prostitution and drug crime hot spots. The Buenos Aires Police Presence after Terror Attack quasiexperiment measured treatment effects on blocks immediately surrounding the block with the protected Jewish center and blocks one removed from the block with the protected Jewish center. For both studies, distinct effect sizes were calculated for each of the two sets of buffer areas.

14 Random effects models were used to estimate the standardized mean effect sizes for the largest and smallest displacement and diffusion effect size analyses. For the largest effect size meta‐analysis, *Q* = 64885.112, df = 39, *p* < .001, *I* ^2^ = 99.940. For the smallest effect size meta‐analysis, *Q* = 17931.884, df = 39, *p* < .001, *I* ^2^ = 99.783.

15 For POP programs, *Q* = 179.543, df = 24, *p* < .001, *I* ^2^ = 86.632. For increased policing programs, *Q* = 162.328, df = 47, *p* < .001, *I* ^2^ = 71.046. The between *Q* = 20.852, df = 1, *p* < .001, suggesting that the hot spots policing program type produced statistically significant differences in observed crime outcomes. The moderated overall effect size was 0.120 (standard error = 0.017, *p *< .001, 95% CI = 0.086, 0.153).

16 The 29 gray literature studies included 32 independent tests of hot spots policing programs and the 36 journal article studies included 41 independent tests of hot spots policing programs. For gray literature studies, *Q* = 73.908, df = 31, *p* < .001, *I* ^2^ = 58.056. For journal article studies, *Q* = 228.913, df = 40, *p* < .001, *I* ^2^ = 82.526. The between *Q* = 42.342, df = 1, *p* < .001, suggesting that the publication type produced statistically significant differences in observed crime outcomes. The moderated overall effect size was 0.125 (standard error = 0.018, *p* < .001, 95% CI = 0.089, 0.161).

17 As discussed by Rothstein (2008, p. 69), the trim‐and‐fill procedure is based on the notion that, in the absence of bias, a funnel plot of study effect sizes will be symmetric about the mean effect. If there are more small studies on one side than on the other side of the bottom of the funnel plot, there is concern that some studies may have been censored from the meta‐analysis. The trim‐and‐fill approach imputes the missing studies, adds them to the analysis, and then recomputes the mean effect size. The most notable limitation is that this approach assumes the observed asymmetry is a result of publication bias rather than of true differences in the results of the small studies compared with the larger ones.

## APPENDIX A LIST OF 146 EXPERTS CONTACTED

Hassan Aden, Vera Institute of Justice

Karen Amendola, Police Foundation

Barak Ariel, Hebrew University

Abhijit Banerjee, Massachusetts Institute of Technology

David Bayley, University at Albany, SUNY

Richard Berk, University of Pennsylvania

Lawrence Bobo, Harvard University

Brenda Bond, Suffolk University

Kate Bowers, University College London

Alfred Blumstein, Carnegie Mellon University

Clairissa Breen, Buffalo State, SUNY

Gerben Bruinsma, Netherlands Institute for the Study of Crime and Law Enforcement

Rod Brunson, Rutgers University

Kevin Bryant, Benedictine College

James Bueerman, Police Foundation

Michael Buerger, Bowling Green State University

George Burruss, University of South Florida

Joel Caplan, Rutgers University

George Capowich, Loyola University, New Orleans

Christine Carr, Kansas City Police Department

Breanne Cave, Police Foundation

Steven Chermak, Michigan State University

Ronald V. Clarke, Rutgers University

Jacqueline Cohen, Carnegie Mellon University

Greg Collins, Shawnee Police Department

Phil Cook, Duke University

Lisa Dario, Florida Atlantic University

Michael Deckard, University of Missouri, St. Louis

Scott Decker, Arizona State University

Esther Duflo, Massachusetts Institute of Technology

John E. Eck, University of Cincinnati

Robin Engel, University of Cincinnati

Jeffrey Fagan, Columbia University

Christine Famega, California State University, San Bernardino

Graham Farrell, University of Leeds

David Farrington, University of Cambridge

Matthew Fielding, Greater Manchester Police

Andrew Fox, California State University, Fresno

Patrick Gartin, Missouri State University

Amanda Geller, Columbia University

Manne Gerell, Malmö University

Andrew Gilchrist, University of Cincinnati

Phillip Goff, John Jay College of Criminal Justice

Herman Goldstein, University of Wisconsin

Peter Grabosky, Australian National University

Jack Greene, Northeastern University

Elizabeth Groff, Temple University

Ben Grunwald, University of Chicago

Cory Haberman, University of Cincinnati

Lauren Hajjar, Suffolk University

Rachel Harmon, University of Virginia

Amelia Haviland, Carnegie Mellon University

Julie Hibdon, Southern Illinois University

Joshua Hinkle, Georgia State University

Natalie Kroovand Hipple, University of Indiana

John Hollywood, RAND Corporation

Timothy Hope, University of Salford

Priscillia Hunt, RAND Corporation

Shane Johnson, University College London

Vincent Jones, Greater Manchester Police

Nola Joyce, Philadelphia Police Department

George Kelling, Manhattan Institue

Daniel Keniston, Yale University

David M. Kennedy, John Jay College of Criminal Justice

Leslie Kennedy, Rutgers University

Dae‐Young Kim, Buffalo State, SUNY

Jonathan Klick, University of Pennsylvania

David A. Klinger, University of Missouri, St. Louis

Johannes Knutsson, Norwegian Police University College

Tammy Rinehart Kochel, Southern Illinois University

Christopher Koper, George Mason University

Janet Lauritsen, University of Missouri, St. Louis

Brian Lawton, John Jay College of Criminal Justice

Gloria Laycock, University College London

Cynthia Lum, George Mason University

John MacDonald, University of Pennsylvania

Tracey Maclin, Boston University

Edward R. Maguire, Arizona State University

Peter Manning, Northeastern University

Charles Manski, Northwestern University

Natalie Martinez, University of Nevada, Las Vegas

Stephen D. Mastrofski, George Mason University

David Mazeika, University of Maryland

Lorraine Mazerolle, University of Queensland

Jack McDevitt, Northeastern University

Edmund McGarrell, Michigan State University

Tracey Meares, Yale University

Linda Merola, George Mason University

Renée Mitchell, Sacremento Police Department

Mark H. Moore, Harvard University

Daniel Nagin, Carnegie Mellon University

Kenneth Novak, University of Missouri, Kansas City

Alexis Norris, California State University, San Bernardino

Emily Owens, University of California, Irvine

Henry Partridge, Manchester Metropolitan University

Ruth Peterson, Ohio State University

Scott Phillips, Buffalo State, SUNY

Glenn L. Pierce, Northeastern University

Alex Piquero, University of Texas, Dallas

Eric Piza, John Jay College of Criminal Justice

Steven Raphael, University of California, Berkeley

Jerry Ratcliffe, Temple University

Justin Ready, Arizona State University

George Rengert, Temple University

Greg Ridgeway, University of Pennsylvania

Dennis Rosenbaum, University of Illinois, Chicago

Richard Rosenfeld, University of Missouri, St. Louis

Jason Rydberg, University of Massachusetts, Lowell

Robert Sampson, Harvard University

Rachel Santos, Radford University

Roberto Santos, Radford University

Jessica Saunders, RAND Corporation

Michael Scott, University of Wisconsin, Madison

Christopher Sedelmaier, University of New Haven

Elaine B. Sharp, University of Kansas

Lawrence Sherman, University of Cambridge

Wesley Skogan, Northwestern University

Evan Sorg, Rowan University

David Spade, University of Missouri, Kansas City

William Spelman, University of Texas, Austin

Marc Swatt, Justice and Security Strategies

Travis Taniguchi, RTI International

Bruce Taylor, University of Chicago

Ralph Taylor, Temple University

Cody Telep, Arizona State University

Timothy Thomas, University of Washington

Nick Tilley, University College London

George E. Tita, University of California, Irvine

Jeremy Travis, John Jay College of Criminal Justice

Tom Tyler, New York University

Craig Uchida, Justice and Security Strategies

Samuel Walker, University of Nebraska, Omaha

Elin J. Waring, Lehman College, CUNY

Cristobal Weinborn, University of Cambridge

David Weisburd, Hebrew University Law School

Alexander Weiss, Alexander Weiss Consulting

Charles Wellford, University of Maryland

Brandon Welsh, Northeastern University

Andrew Wheeler, University of Texas, Dallas

Michael White, Arizona State University

Simon Williams, West Midlands Police

Jeremy M. Wilson, Michigan State University

Jennifer Wood, Temple University

Daniel Woods, Police Foundation

Robert Worden, University at Albany, SUNY

Sue‐Ming Yang, Georgia State University

## APPENDIX C EXCLUDED STUDIES

There were a number of studies identified during the abstract search that were worthy of further consideration but ultimately determined not to meet the inclusion criteria. This appendix notes those studies and provides a brief explanation as to why the study was excluded.

**Table:**

Author(s)	Intervention	Location	Reason for exclusion
Alderden et al. (2011)	Gang Hot Spots Policing in Chicago—The Deployment Operations Center	Chicago, IL	Target area too large
Andresen (2015)	Increased Foot Patrol in Lower Lonsdale	Lower Lonsdale, British Columbia	No comparison area
Andresen and Lau (2014)	Increased Foot Patrol in Lower Lonsdale	Lower Lonsdale, British Columbia	No comparison area
Andresen and Malleson (2014)	Increased Foot Patrol in Lower Lonsdale	Lower Lonsdale, British Columbia	No comparison area
Barthe and Stitt (2011)	Increased Police Presence in a Non‐Criminogenic Area	Reno, NV	Target area too large
Bynum et al. (2014)	Project Safe Neighborhoods in Detroit	Detroit, MI	No hot spots policing component
Corsaro et al. (2012)	Crash Analysis Reduction Strategy	Cincinnati, OH	Target area too large
Crank et al. (2010)	Omaha Metro Safety Initiative	Omaha, NE	No comparison area
Frogner et al. (2013)	§ Project in Sweden	Orebro, Sweden	No comparison area
Gorr and Lee (2015)	Policing Hot Spots via the Early Warning System	Pittsburgh, PA	Study was a simulation and did not assess an actual intervention
Guseynov (2010)	CSTAR Projects in Kansas City	Kansas City, MO	Target area too large
Hall and Puls (2010)	DDACTS in Baltimore County	Baltimore County, MD	Target area too large
Heaton et al. (2016)	Expanded Private University Police Patrol in Chicago	Chicago, IL	No hot spots policing component
			Target area too large
Hoover et al. (2016)	Houston Enhanced Action Patrol	Houston, TX	Target area too large
			No separate comparison area
Hunt et al. (2014)	Shreveport Predictive Policing Experiment	Shreveport, LA	Control group did not receive “business as usual” policing
Jang et al. (2012)	Hot Spots Policing with Dallas PD's Disruption Unit	Dallas, TX	No control area
Kim et al. (2016)	Symbolic SWAT Raids in Buffalo	Buffalo, NY	Target area too large
			No follow‐up enforcement activity
Klick and Tabarrok (2005)	Terror Alerts as Shocks to Police Presence	Washington, DC	No comparison area
MacDonald et al. (2016)	Expanded Private University Patrol in Philadelphia	Philadelphia, PA	No hot spots policing component
			Target area too large
Maskaly (2009)	Drug Crackdown by Reno PD's Street Enforcement Team	Reno, NV	Evaluation did not analyze the target area
			No comparison area
McClure et al. (2014)	DDACTS in Multiple Sites	15 sites, US	No outcome evaluation
McLean et al. (2010)	Operation Safe Corridor	Ashton, England	Target area too large
Melenka (2016)	Increased Foot Patrol in Lower Lonsdale	Lower Lonsdale, British Columbia	Target area too large
Mohler et al. (2015)	Randomized Controlled Field Trials of Predictive Policing	UK and US	Control group did not receive “business as usual” policing
Papazian (2013)	HALO Camera Surveillance System in Denver	Denver, CO	No added patrol or dedicated viewer
Piza et al. (2014)	Increased CCTV in Newark	Newark, NJ	No hot spots policing component
Ratcliffe et al. (2017)	Operation Thumbs Down	Los Angeles, CA	Target area too large
Silverii (2010)	DDACTS in Lafourche Parish	Lafourche Parish, LA	Target area too large
Wells and Wu (2011)	Proactive Policing by Houston PD's Crime Reduction Unit	Houston, TX	No control group without police activity
Wells et al. (2012)	Proactive Policing by Houston PD's Crime Reduction Unit	Houston, TX	No control group without police activity
Worrall (2016)	Smart Policing Initiative in Frisco	Frisco, TX	No control group without police activity
Williams and Chernoff (2013)	Initiative: Laser Point	Manhattan, KS	All hot spots received treatment and the comparison group was crime in the previous year

## APPENDIX D DETAILED NARRATIVE REVIEW OF ELIGIBLE HOT SPOTS POLICING EVALUATIONS

### Minneapolis (MN) Repeat Call Address Policing (RECAP) Program

In the Minneapolis RECAP program, a randomized controlled trial was used to test the effects of problem‐oriented policing on commercial and residential addresses that generated large volumes of calls for service to the police (Sherman et al., 1989). The 452 commercial and residential addresses that generated the high numbers of calls for service to the Minneapolis Police Department over a 1‐year period were identified via a simple ranking procedure and included in the experiment. A specialized unit of one sergeant and four patrol officers were assigned to implement the problem‐oriented policing strategy at treatment addresses for a 1‐year intervention time period. After simple random allocation procedure was completed, 107 commercial addresses and 119 residential addresses received the problem‐oriented policing. The calls for service during the baseline year (1986) were compared with calls for service during the intervention year (1987) to estimate the effect of the problem‐oriented policing intervention on the treatment commercial and residential addresses. Subsequent accounts of the of the RECAP treatment noted some innovative problem solving but generally described a problem‐oriented policing intervention comprised of traditional law enforcement actions, referrals to social services, informal counseling by police, and modest changes to the physical environment (Buerger, 1992, 1993).

The evaluation noted several issues with the execution of the research design (Sherman et al., 1989). The two most important were: (a) by chance alone, the simple randomization procedure resulted in many of the most active addresses to be allocated to treatment conditions; the instability between control and treatment groups resulted in reduced statistical power to detect a treatment effect, and (b) the specialized unit was understaffed to deal with 226 high‐activity addresses and the resulting treatment dosage was low. Analyses of pre‐post differences in calls for service revealed no statistically significant differences for the treatment commercial addresses relative to the control commercial addresses. However, analyses of pre‐post differences in calls for service at treatment residential addresses relative to control residential addresses revealed a statistically significant 15% reduction in calls in the first 6 months that declined to 6% in the first full year.

### New York (NY) Tactical Narcotics Teams (TNT)

The New York Police Department first launched the TNT program in May 1988 to by allocating a team of officers to a drug‐plagued area in Queens; by 1989, TNT was operating in locations throughout New York City (Sviridoff et al., 1992). The TNT intervention was designed as a mobile overlay of resources to supplement existing police staffing in particular areas suffering from disorderly street‐level drug market problems and was comprised of plainclothes and undercover officers who relied upon “buy and bust” operations to disrupt local drug markets. TNT deployments lasted for 90 days followed by “maintenance” of high visibility police presence. Beginning in 1989, the Vera Institute of Justice completed an external 2‐year study of TNT operations.

The Vera impact evaluation used a quasiexperimental design and measured the impact of TNT on assault, robbery, and burglary incidents in two treatment precincts, the 67th and 70th, relative to one comparison precinct, the 71st (Sviridoff et al., 1992). Entire precincts were not treated as research sites; rather the evaluation focused on TNT impacts in small drug market areas defined as “particular streets, intersections, sets of buildings, or other ‘hot spots’” (p. 12). The Vera evaluation also included pre‐ and postintervention surveys of community residents and a number of pre‐post qualitative interviews in the targeted areas. The evaluation noted some implementation difficulties in the study precincts that included diminished resources and arrests in the 67th precinct and a shorter than planned maintenance period in the 70th precinct. Auto regressive integrated moving average (ARIMA) interrupted time series models found that the TNT intervention did not generate statistically significant reductions in assault, robbery, and burglary incidents in the 70th precinct. However, in the 67th precinct, ARIMA time series models found a statistically significant reduction in assault incidents associated with the TNT intervention but no statistically significant reductions in robbery or burglary. The community survey and interviews suggested that TNT did not improve community perceptions of disorder, reduce fear of crime, increase use of public amenities, or improve community attitudes toward the police.

### St. Louis (MO) Problem‐Oriented Policing at Three Drug Market Locations

Hope (1994) documented three case studies that were part of a “Community Oriented Problem Solving” initiative launched by the St. Louis Metropolitan Police Department in 1991. In the three case studies, specific addresses associated with street‐level drug sales were targeted for focused police attention. In the case studies, Hope (1994) described problem‐oriented policing interventions comprised of mostly traditional enforcement tactics with some situational responses. These situational responses included housing code enforcement and boarding up and securing buildings. The problem‐oriented policing intervention period lasted for 9 months.

The evaluation of the interventions in the three case studies used a quasiexperimental design; changes in citizen calls at hot spot addresses location were compared to changes in calls at other addresses on the block as well as other blocks in surrounding areas (Hope, 1994). Simple trend and OLS regression analyses examined citizen calls for service during the 9‐month intervention as well as 12‐month preintervention and 6‐month postintervention periods. The evaluation reported that all three drug locations experienced varying reductions in total calls. Regression analysis suggested that reductions on blocks where drug locations were located were greater than other blocks and intersections in surrounding areas. Hope (1994) also examined immediate spatial crime displacement and diffusion of crime control benefits by comparing trends in calls at targeted addresses to trends in calls at other addresses on same block. He reported mixed results. In case study 1, the intervention seemed to generate significant displacement into surrounding addresses. However, in case studies 2 and 3, he did not find any significant displacement or diffusion effects.

### Minneapolis (MN) Hot Spots Patrol Program

The Minneapolis Police Department collaborated with academic researchers to re‐examine the deterrent effects of police patrol on crime (Sherman & Weisburd, 1995). The landmark Kansas City Patrol Experiment concluded that varying levels of police patrol had no significant effects on crime (Kelling et al., 1974). The Minneapolis redesign of the Kansas City Patrol Experiment addressed two limitations of the original design. First, the small number of areas (15 patrol beats) in the Kansas City experiment resulted in weak statistical power of the design to detect an effect. Second, the police patrol treatment was diffused across relatively large areas (patrol beats); as such, the dosage level of the police patrol intervention applied to the treatment areas may not have been enough to generate a deterrent effect. The research team identified 110 hot spots based on clustering of calls for service at specific addresses and consideration of researcher observations of appropriate place boundaries. These 110 hot spots were allocated to treatment and control conditions in five statistical blocks (resulting in 55 treatment hot spots and 55 control hot spots). The analysis compared calls for service at treatment locations relative to control locations for a baseline year relative to a treatment year.

On the basis of the observations of trained researchers, the treatment hot spots received twice as much police patrol presence when compared with the control hot spots (Sherman & Weisburd, 1995). The study authors noted that there was some breakdown in the treatment applied during summer months due to officer vacations and peak calls for service to the police department. The authors conducted a sensitivity analysis with varying comparison dates to account for the lack of dosage during the summer months. Using a series of analysis of variance models, the authors reported that the police patrol treatment generated between 6% and 13% statistically significant reductions in calls for service in treatment hot spots relative to calls for service in control hot spots. Analyses of systematic social observation data on disorderly behavior in the hot spots collected by trained researchers suggested that observed disorder was only half as prevalent in treatment hot spots relative to control hot spots.

### Jersey City (NJ) Drug Market Analysis Program

The Jersey City Police Department collaborated with the Center for Crime Prevention Studies at Rutgers University to design and implement a randomized controlled trial to evaluate the effects of a problem‐oriented drug enforcement strategy at drug hot spots in Jersey City, New Jersey (Weisburd & Green, 1995a). Using computer mapping technology supplemented by perceptions of Jersey City narcotics officers of drug market boundaries, the research team identified 56 drug hot spots that were randomly allocated in statistical blocks to treatment and control conditions (28 treatment hot spots and 28 control hot spots). The treatment followed a stepwise strategy that encouraged business owners and residents to be engaged in crime control efforts, implemented carefully designed crackdowns focused on dealers operating in targeted drug hot spots, and employed a postcrackdown maintenance of targeted areas by heightened uniform patrol presence. The control drug markets experienced unsystematic arrest‐oriented narcotics enforcement activity that represented the routine drug enforcement work pursued by the Jersey City Police Department's narcotics squad.

The randomized controlled trial used mixed‐model analysis of variance methods to compare calls for service during 7‐month preintervention to calls for service during 7‐month postintervention time periods at the treatment and control drug hot spots (Weisburd & Green, 1995a). The analysis revealed statistically significant reductions in disorder calls for service in the treatment drug markets relative to the control drug markets. Violent and property calls for service were not significantly impacted by the intervention. The research team also used mixed‐model analysis of variance methods to compare calls for service during 7‐month preintervention to calls for service during 7‐month postintervention time periods at the two‐block buffer zones surrounding the treatment and control drug hot spots. The analysis revealed a statistically significant reduction, or diffusion of benefits effect, in public morals and narcotics calls for service in the treatment buffers relative to control buffers. Finally, the research team also replicated the drug market identification process similar to what was employed to identify the original study drug market locations. This exercise suggested that drug market activity was twice as likely to be found in areas surrounding the control drug hot spots relative to areas surrounding the treatment drug hot spots.

### Kansas City (MO) Gun Project

The Kansas City Gun Project examined the gun violence prevention effects of proactive patrol and intensive enforcement of firearms laws via safety frisks during traffic stops, plain view searches and seizures, and searches incident to arrests on other charges (Sherman & Rogan, 1995a). The quasiexperimental evaluation focused on testing the hypothesis that gun seizures and gun crimes would be inversely related. In other words, an increase in the number of guns seized in the targeted location would be associated with a decrease in gun crimes in the targeted location. The Gun Project intervention was limited to one target patrol beat that was matched to a comparison beat with nearly identical numbers of drive‐by shootings in 1991. Simple computer analyses of call and incident data were used to focus police interventions at hot spot locations within the targeted beat. A pair of two‐officer cars, working overtime from 7 p.m to 1 a.m. 7 days a week and not required to answer citizen calls for service, provided extra patrol in the targeted beat. The officers initiated a high volume of contact with the street population. During 29 weeks in 1992–1993, the directed patrols resulted in 1,090 traffic citations, 948 car checks, 532 pedestrian checks, 170 state or federal arrests, and 446 city arrests (Sherman & Rogan, 1995a). The comparison beat received routine levels of police activities.

Sherman and Rogan (1995a) used a variety of quantitative methodologies, including before and after difference of means, ARIMA time series models, and analysis‐of‐variance models, to evaluate the gun crime data. The quasiexperimental evaluation revealed that proactive patrols focused on firearm recoveries resulted in a statistically significant 65% increase in gun seizures (29 additional guns seized) and a statistically significant 49% decrease in gun crimes in the target beat area (83 fewer gun crimes); gun seizures and gun crimes in the comparison beat area did not significantly change (Sherman & Rogan, 1995a). The Kansas City Gun quasiexperiment also used before and after difference of means tests and ARIMA time series analyses to examine whether gun crimes were displaced into seven beats contiguous to the target beat. None of the contiguous beats showed significant increases in gun crime and two of the contiguous beats reported significant decreases in gun crimes.

A separate nonequivalent control group quasiexperiment examined community reaction to the Kansas City intervention and, through surveys of randomly selected residents in the treatment and control areas, found that the community strongly supported the intensive patrols and perceived an improvement in the quality of life in the treatment neighborhood (Shaw, 1995). In contrast to broader concerns about the effects of proactive policing programs on police–community relations, the Kansas City hot spots patrol program apparently did not increase community tensions. The research did not, however, attempt to measure the views of persons stopped by police patrolling in the hot spot areas. Shaw (1995) presents data revealing that two‐thirds of all persons arrested for illegally carrying concealed weapons in the target area in 1992 did not live in the target area. Shaw (1995) suggests that most offenders in gun hot spot areas may be outsiders who come only for trouble and, as such, the street population who are stopped and checked by the police may have very different views from the residents of that area.

### Kansas City (MO) Crack House Police Raids Program

The Kansas City (MO) Police Department collaborated with researchers from the Crime Control Institute and the University of Maryland to test the deterrent effects of uniformed police raids of crack houses on block‐level crime and disorder (Sherman & Rogan, 1995b). Using a randomized controlled trial, the research design required all eligible cases to be drawn from blocks with at least five calls for service in the 30 days preceding an undercover drug buy made at the inside of a residence. All cases had to be eligible for a search warrant (as judged by Street Narcotics Unit officers) before random assignment occurred. Of 207 eligible cases, court‐authorized raids were randomly allocated to 104 blocks and were conducted at 98 of those sites; the other 103 blocks did not receive raids.

The analysis followed an “intention‐to‐treat” plan in which cases were analyzed according to random assignment to treatment rather than the treatments actually received (Sherman & Rogan, 1995b). Negative binomial regression models were used to analyze citizen calls for service and offense reports during 30‐day preintervention and 30‐day postintervention time periods at treatment blocks relative to control blocks. The evaluation reported modest decreases in citizen calls (*p* = .06) and offense reports (*p *= .15) at treatment blocks relative to control blocks that decayed in 2 weeks.

### Beenleigh (AUS) Calls for Service Project

The Criminal Justice Commission and the Queensland Police Service launched the Beenleigh Calls for Service Project in September 1996 to determine whether problem‐oriented policing would reduce the number of calls for service to the Beenleigh Police Division (Criminal Justice Commission, 1998). At the time of the project, Beenleigh was described as a lower‐income suburb with a population of some 40,000 residents. The Criminal Justice Commission's Research Division analyzed calls for service data for the Beenleigh Police Division and identified two groups of ten addresses that experienced the highest volume of calls during separate 6‐month periods. These 20 addresses then received the problem‐oriented policing treatment for a 6‐month intervention period. The problem‐oriented interventions were comprised of increased police presence at the targeted addresses, providing crime prevention information and advice to people at the targeted addresses, altering the physical environment (such as trimming bushes and shrubs), and making referrals of problems to other agencies (Criminal Justice Commission, 1998, p. x–xi).

The Criminal Justice Commission (1998) research team used a quasiexperimental design to compare calls for service trends in Beenleigh to calls for service in the matched town of Browns Plains. Simple time series analyses of total monthly calls for service in 5‐month pretest, 6‐month intervention, and 3 month posttest periods found no noteworthy differences in the total number of calls in the town of Beenleigh relative to the matched town of Browns Plains (Criminal Justice Commission, 1998, p. 25). However, simple nonexperimental pre/post comparisons found noteworthy reductions in total citizen calls for service in 16 of 19 case studies included in the report. The research team concluded that the problem‐oriented policing strategy enjoyed some success in reducing calls for service at the targeted locations, but due to the small scale of the project and limitations of the research design, these crime prevention gains were not large enough to be detected at the aggregate town level (Criminal Justice Commission, 1998, p. 28).

### Jersey City (NJ) Problem‐Oriented Policing at Violent Places Project

The Jersey City Police Department collaborated with researchers from Rutgers University's Center for Crime Prevention Studies to evaluate the effects of problem‐oriented policing interventions on high‐activity violent crime places (Braga et al., 1999). Using computerized mapping and database technologies, 24 violent crime places were identified based on ranking intersection areas with high levels of assault and robbery calls and incidents, and police and researcher perceptions of violent areas. These 24 high activity violent crime places were matched into twelve pairs and one member of each pair was allocated to treatment conditions in a randomized block field experiment. The treatment consisted of problem‐oriented policing interventions comprised of mostly aggressive disorder enforcement tactics with some situational responses. The duration of the intervention time period was 16 months.

Using Poisson regression models, the main analyses examined the differences of differences between a number of indicators during 6‐month pre‐ and posttest periods, comparing control and experimental groups. The analyses found that the treatment resulted in statistically significant reductions in total calls for service and total crime incidents, as well as varying reductions in all subcategories of crime types, in the treatment violent crime hot spots relative to controls (Braga et al., 1999, pp. 562–563). Analyses of systematic observation data collected during the pre‐ and posttest periods revealed that social disorder was alleviated at 10 of 11 treatment places relative to controls (Braga et al., 1999, p. 564).^18^ Nonexperimental systematic observation data collected pre‐ and posttest at treatment places suggested that physical disorder was alleviated at 10 of 11 treatment places (Braga et al., 1999, p. 564).^19^ Pre‐ and posttest interviews with key community members suggested that community perceptions of places improved at 7 of 12 treatment places (Braga, 1997, pp. 235–236). The research team also used experimental analyses to examine displacement and diffusion effects in two‐block catchment areas surrounding the treatment and control violent crime places. The analyses found little evidence of immediate spatial displacement or diffusion effects.

### Houston (TX) Targeted Beat Program

Between 1994 and 1996, the Houston Police Department launched the Targeted Beat Program to reduce Part I crimes in the seven highest crime beats in the city (Caeti, 1999). Funds were allocated to use overtime officers to saturate these seven beats; computer analyses were used to further target enforcement actions at specific hot spots locations within the treatment beats. The Houston Police Department used varying crime reduction strategies across the seven targeted beats: three beats used “high visibility patrol” at hot spots, three beats used “zero tolerance” policing at hot spots, and one beat used a problem‐oriented policing approach comprised of mostly traditional tactics to control hot spots. The intervention period lasted for 2 years.

Caeti (1999) used a quasiexperimental design to estimate treatment effects of the Houston Targeted Beat Program; target beats were matched to noncontiguous comparison beats through cluster analysis and correlations of Census data. Unfortunately, the results of the Houston Targeted Beat quasiexperiment must be interpreted with caution. The key analytic measures of effectiveness were comparisons of pre‐ and posttest differences (as measured by *t* tests) in reported crime incidents at treatment beats relative to control beats (Caeti, 1999, p. 319–322). However, the analyses did not examine the differences of differences between treatment and control areas. As such, the quasiexperimental analyses did not directly measure whether observed changes in treatment beats were significantly different from observed changes in control beats. Reported statistically significant reductions in treatment beats relative to nonsignificant decreases and any increases in reported crime can be interpreted with caution as a treatment effect. However, conclusions that the program did not work in treatment beats with reported significant crime reductions relative to control beats with significant crime reductions were not justified. It was completely possible that the observed significant reductions in the treatment beats were significantly greater than the significant reductions in control beats.

Given these caveats, the Houston Targeted Beat quasiexperiment suggests that the aggregated treatment beats experienced significant reductions in auto theft, total Part I index crimes,^20^ and total Part I “patrol suppressible” crimes (robbery, burglary, and auto theft) relative to aggregated control beats. The three treatment beats where “zero tolerance” aggressive disorder policing was used to control hot spots experienced mixed reductions in Part I crimes relative to control beats; the three treatment beats where “high visibility” directed patrol was used to control hot spots experienced reductions in a wide variety of Part I crimes relative to control beats; the one treatment beat where an enforcement problem‐oriented policing strategy was implemented to control hot spots did not experience noteworthy decreases relative to a control beat. The limits of the analytic framework preclude conclusions that certain types of policing strategies may be more effective in preventing crime in hot spots. Nevertheless, the results of this study can be broadly taken to support the position that focused police enforcement efforts can be effective in reducing crime at hot spots.

The Houston Targeted Beat quasiexperiment examined displacement and diffusion effects by conducting simple pre/post comparisons of reported Part I index crimes in beats contiguous to the treatment beats. The analyses revealed no overall evidence of displacement and contiguous beats surrounding three targeted beats (one problem‐oriented policing beat and two “zero tolerance” beats) experienced possible diffusion effects as several types of reported Index crimes decreased notably.

### Oakland (CA) Beat Health Program

The Oakland Police Department's Beat Health program was a problem‐oriented policing intervention designed “to control drug and disorder problems, in particular, and restore order by focusing on the physical decay conditions of targeted commercial establishments, private homes, and rental properties” (Mazerolle et al., 2000, p. 213). The Oakland Police officers collaborated with teams of city agency representatives to inspect drug nuisance properties, coerce landowners to clean up blighted properties, post “no trespassing” signs, enforce civil law codes and municipal regulatory rules, and initiate court proceedings against property owners who fail to comply with civil law citations. The program evaluation used a randomized controlled trial to determine the impact of the Beat Health civil remedy program (treatment group) relative to the impact of the routine policing activities of the regular patrol division (control group) on street blocks in Oakland, California (Mazerolle et al., 2000).

Street blocks were eligible for inclusion in the evaluation when a residential or commercial property on a street block was referred to the Beat Health Unit as having a drug and/or blight problem. Control and treatment groups were each randomly allocated 50 street blocks within residential and commercial statistical blocks (total *N* = 100). The experimental analysis used the differences of differences design; pre‐post time periods were 21.5 months before and 12 months after the 5.5‐month intervention period. The research design also explicitly examined displacement and diffusion effects in 500 foot radii catchment areas surrounding the treatment and control street blocks. Mazerolle et al. (2000) found that the Beat Health program generated a statistically significant reduction in drug calls in treatment blocks relative to control blocks but no statistically significant differences in other call types. Analyses of catchment areas suggested an overall diffusion of crime control benefits for treatment catchment areas relative to control catchment areas.

### Pittsburgh (PA) Police Raids at Nuisance Bars Program

Concerned about an apparent association between bars and drug dealing, the Pittsburgh (PA) Police Department established the Nuisance Bar Task Force which included prosecutors, liquor control, code enforcement agencies, and community representatives (Cohen et al., 2003). Nuisance bars were initially identified through calls to the Mayor's “Bar Hot‐Line” and to the police narcotics and vice squads; nuisance bars were then officially targeted after plainclothes detectives verified reports of drug dealing and other disorder problems in and around the business premises. After designation as a nuisance bar, it was subjected to raids by the narcotics squad. The evaluators examined raids at 37 nuisance bars conducted between January 1990 and December 1992 (Cohen et al., 2003). Nuisance bars received an average of 3.7 raids per month during enforcement periods that lasted between one (43%) and 5 months (18%).

The evaluators used a quasiexperimental design to compare trends in drug calls for service in targeted nuisance bar areas relative to trends in drug calls for service in nonnuisance bar areas (Cohen et al., 2003). The units of analysis were 660 foot areas (2–3 blocks in either direction) surrounding the 37 targeted nuisance bars and 40 nonnuisance bars located in the same neighborhoods. To estimate intervention impacts, the evaluators used OLS and Tobit regression models that controlled for land‐use and population‐based risk factors, secular trends, serial autocorrelation, length of enforcement periods, and the number of raids. The evaluators concluded that the police raids resulted in statistically significant reductions in drug calls in the treatment areas relative to control areas during periods of active enforcement. These crime control gains largely disappeared when active enforcement ceased.

### Buenos Aires (ARG) Police Presence after Terror Attack Study Initiative

On July 18, 1994, terrorists exploded a bomb at the main Jewish center in Argentina, resulting in 85 deaths and an additional 300 wounded (DiTella & Schargrodsky, 2004). One week after this tragedy, the Argentinean government assigned police protection to all Jewish and Muslim centers in the country. DiTella and Schargrodsky (2004) collected data on the number of motor vehicle thefts per block in three neighborhoods in Buenos Aires for the 9‐month period between between April 1, 1994 and December 31, 1994. The authors then collected information on the location of protected Jewish center on the blocks. The authors used difference‐in differences estimators in Least Squares Dummy Variable regression models to examine the impact of increased police presence on motor vehicle thefts per block for blocks with Jewish institutions (treatment), one‐block away from Jewish institutions, and two‐blocks away from Jewish institutions in three Buenos Aires neighborhoods over a 9‐month period (5 months posttest, 4 months pretest).

The analysis included 37 treatment blocks, 161 blocks one‐block from treatment, 226 blocks two‐blocks from treatment, and 876 total blocks in the analysis. The results found that extra police presence was associated with a statistically significant 75% reduction in motor vehicle thefts on the targeted blocks (DiTella & Schargrodsky, 2004). The extra police presence was not associated with significant immediate crime displacement or diffusion of crime control benefits to blocks surrounding the protected Jewish centers. The regression analysis did not report any statistically significant differences in motor vehicle theft in the blocks that were one‐block from the treatment block and in the blocks that were two‐blocks from the treatment block.

### Philadelphia (PA) Drug Corners Crackdowns Program

The Philadelphia (PA) Police Department launched Operation Safe Streets on May 1, 2002 to crackdown on 214 of the highest drug activity locations by stationing officers at these places 24 hr a day, 7 days a week (Lawton et al., 2005). Of the 214 locations, 34 were defined as the intersection of two streets and 180 were defined as single addresses. The evaluation team created circular 0.1 mile buffers around the 214 treatment locations (equivalent of roughly one city block in Philadelphia). The evaluators developed 73 “matched” 0.1‐mile comparison areas through spatial analyses to identify nontreated high‐activity drug locations elsewhere in Philadelphia and further examination of demographics via simple analyses of 2000 U.S. Census data. Buffer zones, comprised of 0.1 mile areas surrounding treatment areas, were also constructed to examine immediate spatial crime displacement and diffusion of benefits effects.

ARIMA interrupted time series analysis models were used to analyze trends in violent crime incidents and drug crime incidents at treatment areas and comparison areas (Lawton et al., 2005). ARIMA models were also used to examine trends in treatment buffer zones and comparison buffer zones. The time series analyses examined trends in 121 weeks of pretreatment data and 18 weeks of treatment data. The impact analysis revealed that the Operation Safe Streets intervention was associated with statistically significant reductions in violent crime incidents and drug crime incidents at the treatment areas; no significant intervention time period changes in outcomes were noted at the comparison areas. The analyses of the adjoining buffer zones suggested a statistically significant reduction, or diffusion of benefits, for violent crime incidents. The results of the analyses of drug crime incident trends in the adjoining buffer zones were mixed, however. Depending on the specification of the ARIMA model, the intervention either generated a displacement effect (1,0,1) or a diffusion effect (1,0,0).

### Jersey City (NJ) Displacement and Diffusion Study

The Police Foundation collaborated with the Jersey City Police Department on a controlled study to determine whether targeted police action at two high‐activity crime places led to immediate spatial crime displacement or diffusion of crime control benefits in the areas surrounding the targeted places (Weisburd et al., 2006). Crime mapping and database technologies, supplemented by police officer observations, were used two identify the two study locations: a street prostitution hot spot and a very active street‐level drug market. One‐ and two‐block buffer zones (or “catchment areas”) were constructed around the two targeted crime places to measure possible displacement and diffusion effects emanating from the focused police actions in targeted crime places. The interventions at the prostitution and drug hot spots could be broadly described as enforcement problem‐oriented policing interventions comprised of focused traditional police activities with limited situational responses.

The outcome measure in the evaluation were prostitution and drug events occurring during 20‐min observation periods in target and buffer areas as noted by trained observers from the research team (Weisburd et al., 2006). More than 6,000 20‐min observations were made in the target and buffer areas over the course of the study. At the prostitution hot spot location and surrounding catchment areas, the authors used a quasiexperimental design where observed prostitution event trends were examined over a 9‐month period and adjusted for citywide disorder call trends. At the drug crime hot spot location and surrounding catchment areas, the authors used a quasiexperimental design where observed drug‐behavior events were examined over a 9‐month period and adjusted for citywide drug call trends. Difference of means tests were used to evaluate pre‐ versus posttest changes in observed events in targeted areas adjusting for citywide trends in respective call categories.

For the prostitution hot spot location, the authors reported a statistically significant 45% reduction at the targeted location, a statistically significant 61% reduction in catchment area 1, and a statistically significant 64% reduction in catchment area 2. For the drug crime hot spot location, the authors reported a statistically significant 58% reduction at the targeted location, a nonstatistically significant 33% reduction in catchment area 1, and a statistically significant 64% reduction in catchment area 2. Ethnographic research in the neighborhoods and interviews with arrested offenders suggested that offenders in the targeted areas did not simply displace into surrounding areas because the diminished opportunities and increased risks associated with moving were judged to exceed any gains from continuing their criminal behavior in proximate areas.

### Lowell (MA) Policing Crime and Disorder Hot Spots Project

The Lowell Police Department collaborated with Harvard University researchers to implement a randomized controlled trial testing the effects of problem‐oriented policing strategies in reducing crime and disorder problems at hot spots in Lowell, Massachusetts (Braga & Bond, 2008). Spatial analyses of crime and disorder calls for service, coupled with police officer and researcher observations on place boundaries, were used to identify 34 crime and disorder hot spots. These hot spots were matched in like pairs based on simple comparisons of numbers and types of calls for service, place characteristics, and neighborhood demographics. One member of each pair was randomly allocated to treatment conditions in a randomized block field experiment. The treatment consisted of problem‐oriented policing interventions comprised of mostly aggressive disorder enforcement tactics with some situational responses. The duration of the intervention time period was 12 months.

Using count‐based regression models, the main analyses examined the differences of differences between a number of indicators during 6‐month pre‐ and posttest periods, comparing control and treatment groups. The analyses found that the treatment resulted in statistically significant reductions in total calls for service, as well as varying reductions in all subcategories of crime types, in the treatment hot spots relative to controls (Braga & Bond, 2008). Analyses of systematic observation data collected during the pre‐ and posttest periods revealed that social disorder was alleviated at 14 of 17 treatment places relative to controls (Braga & Bond, 2008). Additional analyses of systematic observation data collected during the pre‐ and posttest periods revealed that physical disorder was alleviated at 13 of 17 treatment places relative to controls (Braga & Bond, 2008). A mediation analysis of the core treatment elements suggested that the crime and disorder gains were driven by situational responses rather than increased misdemeanor arrests or police‐led social service actions.

Pre‐ and posttest interviews with key community members suggested that they noticed an increased police presence and disorder problems were positively impacted in treatment places relative to control places (Braga & Bond, 2009). However, the respondents did not detect any significant changes in police strategy, the willingness of the police to work with residents, or the demeanor of the police toward citizens. The research team also used experimental analyses to examine displacement and diffusion effects in two‐block catchment areas surrounding the treatment and control hot spots. The analyses found little evidence of immediate spatial displacement or diffusion effects.

### Jacksonville (FL) Policing Violent Crime Hot Spots Program

The Police Executive Research Forum collaborated with the Jacksonville Sheriff's Office to implement a randomized controlled trial to test the crime control effects of problem‐oriented policing and direct‐saturation patrol at treatment violent crime hot spots relative to control violent crime hot spots (Taylor et al., 2011). The research team used spatial analyses to identify 83 “street violence” hot spots that average 0.02 square miles in size. These 83 violent crime hot spots were then randomly allocated within statistical blocks to problem‐oriented policing treatment (*N *= 22), direct‐saturation patrol treatment (*N *= 21), and control conditions (*N *= 40). The problem‐oriented policing and direct‐saturation patrol treatments lasted for 90 days. The problem‐oriented policing treatment was comprised of enforcement initiatives and situational crime prevention measures; Taylor et al. (2011) reported that 283 problem‐oriented interventions were implemented across the 22 treatment locations.

The PERF research team compared 1‐year pretreatment outcomes to 90‐day posttreatment outcomes and used Poisson and negative binomial regressions to estimate difference in differences treatment effects on violent and property crime calls and incidents (Taylor et al., 2011). The problem‐oriented policing intervention was associated with a statistically significant 33% reduction in “street violence” and other noteworthy reductions in violence and property crime during the 90 days following the intervention. The direct‐saturation patrol treatment was not associated with any statistically significant reductions in violent and/or property crimes. Using the same analytic framework, the PERF research team examined displacement and diffusion effects in 500 feet buffers surrounding the treatment and control hot spots. The analysis suggested that violent crime problems may have been displaced from problem‐oriented policing treatment hot spots into the surrounding buffer zones. The analysis did not find any noteworthy treatment or diffusion results associated with the direct‐saturation patrol intervention.

### Philadelphia (PA) Foot Patrol Program

The Philadelphia Police Department collaborated with Temple University researchers to implement a randomized controlled trial to determine whether foot patrol prevents crime at violent crime hot spots (Ratcliffe et al., 2011). The research team identified 120 hot spots based on spatial and temporal analyses of “street” violent crime incidents occurring between 2006 and 2008. The research team also considered the perceptions of Philadelphia Police commanders in the determination of hot spot boundaries. The 120 hot spots were ranked by volume of violent crime incidents, matched into like pairs, and then randomly allocated to treatment (*N *= 60) and control conditions (*N *= 60). The treatment was comprised of pairs of officers patrolling on foot in shifts covering 10 a.m. through 2 a.m. the next morning from Tuesday through Saturday each week. The intervention period lasted 12 weeks over the summer of 2009.

The Temple University research team used inverted ORs and linear regression models to estimate the differences of differences in street violent crime incidents during the intervention periods to street violence incidents during the preintervention periods for the treatment and control hot spots (Ratcliffe et al., 2011). The analysis revealed that the foot patrol treatment generated a statistically significant 23% reduction in violent crime incidents in the treatment hot spots relative to the control hot spots. Buffer areas were constructed by the research team around the study hot spots. Subsequent analyses of violent crime in the buffer areas suggested that some violent crime was displaced from foot patrol hot spots into the surrounding areas; however, Ratcliffe et al. (2011) concluded that the violent crime control gains in the treatment areas exceeded the violent crime displacement into the surrounding areas.

### Boston (MA) Safe Street Teams Program

The Boston Police Department launched the Safe Street Teams hot spots policing in January 2007 to address a recent increase in violent crime (Braga et al., 2011). Using computerized mapping technology and qualitative judgments on place boundaries, the Boston Police Department identified 13 violent crime hot spots to receive a Safe Street Team. Each team was staffed by one sergeant and six police officers. These teams were required to remain in their designated areas and implement problem‐oriented policing interventions to address violent crime problems in their hot spot areas. The teams implemented problem‐oriented policing interventions that were predominately characterized by increased enforcement initiatives and limited situational crime prevention responses (Braga et al., 2011).

A nonrandomized quasiexperimental design was used to evaluate the violent crime control benefits of the Safe Street Team program at treated street segments and intersections relative to untreated street segments and intersections (Braga et al., 2011). Propensity score matching techniques were used to identify equivalent comparison places in Boston. Growth curve regression models were use to analyze violent crime trends at treatment street units (*N *= 478) relative to comparison street units (*N* = 564). The preintervention period included yearly counts of violent index crimes between 2000 and 2006 time period while the intervention time period included yearly counts of violent index crimes between 2007 and 2009. The analysis revealed that the Safe Street Team program was associated with a statistically significant 14% reduction in violent crime at treatment street units relative to comparison street units. Using the same analytical framework, the evaluators also examined violent crime trends at street units in two‐block zones surrounding the treatment street units relative to control street units. The growth curve regression models did not report statistically significant spatial crime displacement or diffusion of crime control benefits effects.

### DDACTS Program in Washoe County (NV)

In contrast to prior DDACTS programs that have predominately occurred in urban environments, Washoe County implemented a DDACTS initiative in a largely unincorporated and suburban setting (Beck, 2010). This program was primarily a directed patrol effort that was implemented in two different locations, with each site receiving two iterations of treatment (Beck, 2010). The effects of this program on Part I and Part II index crimes and calls for service was evaluated using a quasiexperimental design. Each of the two distinct treatment police beats were matched with two comparison police beats that had comparable geographic and economic characteristics. Notably, although treatment targeted zones within two separate police beats, the analysis was performed at the beat‐level.

The impact of the intervention was estimated using ANOVA that compared crime in treatment and control areas, separately, before and after the intervention (Beck, 2010). Analyses revealed that neither treatment or comparison areas experienced significant changes in crime incidents or calls for service in the 4 weeks before and 4 weeks after the intervention. Beck (2010) posits one reason for the null effects may have been insufficient treatment dosage; specifically, the level of enforcement activities that took place in the target areas was not substantially greater than areas that did not receive treatment.

### Safer Cities Initiative in Los Angeles (CA)

Los Angeles County is home to one of the largest homeless populations in the United States and, in 2004, the media began providing extensive coverage to the densest concentration of homeless in Los Angeles known as “Skid Row” (Berk & MacDonald, 2010). In September 2005, LAPD launched a pilot project called the “Main Street Pilot Project” in the Historic District of downtown Los Angeles. The pilot project was primarily an order maintenance policing strategy that sought to break up the density of homeless encampments through fines and citations, as well as cracking down on public disorder offenses (including public intoxication, drug use, and prostitution). Personnel involved in the pilot project included 4 to 5 foot patrol officers engaging in order maintenance policing, the deployment of a mobile police command station, undercover teams working in open‐air drug markets and areas known for prostitution, and a specialized undercover unit focused on robberies. Anecdotal evidence suggested that the pilot project was a success, which led to the expansion of the Safer Cities Initiative (SCI) in September 2006. The SCI was a place‐based strategy that sought to break up homeless encampments and reduce nuisance crime, property crime, and violent crime (Berk & MacDonald, 2010). Officers gradually worked through specific areas of “Skid Row” by providing visible police presence for at least 1 week before moving onto the next section. The media and LAPD deemed the project a success as the homeless encampments were cleared, homeless individuals dispersed, debris cleared, and crime and drug overdoses declined.

Berk and MacDonald (2010) offer an independent and rigorous quasiexperimental evaluation of the true impact of the SCI across three outcomes: violent crime, property crime, and nuisance crime. The unit of analysis in the evaluation was larger than the area that received treatment: treatment was delivered in a specific area (“Skid Row”) and analyses were performed at the police division level. Four police divisions adjacent to the treatment division served as the comparison group. A time series analysis examined weekly crime counts from January 1, 2000 to December 31, 2007. Analytic techniques to test the effects of the intervention relied upon generalized additive regression. Crime displacement was examined using the four police divisions adjacent to the treatment division.

Results demonstrated support for the intervention as a crime reduction strategy. Both the pilot project and expanded SCI were associated with statistically significant (*p* < .05) decreases in nuisance crime, violent crime, and property crime (Berk & MacDonald, 2010). While there was no evidence of crime displacement, there was evidence of a diffusion of benefits for both iterations of the project. Total crime was significantly lower in the four police divisions adjacent to the treatment division following the implementation of the pilot project and expanded SCI.

### License Plate Reader Patrols in Crime Hot Spots in Two Adjacent Jurisdictions

Lum et al. (2011) examined the effects of license plate readers on total crime and auto‐related crime (auto theft, theft from auto, and other auto‐related crimes (e.g., driving under the influence and reckless driving)) in two adjacent jurisdictions in Virginia: Alexandria City and Fairfax County in Virginia. A block randomized controlled experimental design stratified by jurisdiction was used to assign 15 hot spots to the treatment group and 15 hot spots to the control group (Lum et al., 2011). Each jurisdiction had two LPR units available and each LPR unit received a list of hot spots to visit for 30 min each. Negative binomial regression that controlled for seasonality was used to examine changes in crime before, during, and 30 days after the intervention.

There were no significant differences between treatment and control groups in weekly counts of total crime during or after the intervention (Lum et al., 2011). Furthermore, no offense‐specific deterrent effect was observed. LPR treatment was not significantly related to auto theft or auto‐related crime. The location of the limited number of hot spots inhibited a formal evaluation of crime displacement but results from a sensitivity analysis that included a dummy variable for areas adjacent to experimental hot spots did not substantially differ from the main analysis. Lum et al. (2011) posited that weak treatment intensity may be responsible for the null effects; specifically, due to resource limitations, “there was likely only a single vehicle involved in an experiment hot spot at any given time” (pp. 340). Additionally, the authors noted that the small sample size used in this study made it difficult to detect a small effect if one was indeed present.

### Camden 28‐Day Crime Suppression Operation in Camden (NJ)

The Camden Police Department launched a series of crime suppression operations throughout 2004 and 2005. Ratcliffe and Breen (2011) evaluated one of those operations that consisted of high‐visible uniformed patrols at crime hot spots known as the “28‐Day Crime Suppression Operation” using a quasiexperimental design. Burglary, violent crime, drug crime, and vehicle crime in the target area was compared to crime in the remainder of the city. Levels of crime in preintervention (November 3, 2004 to December 21, 2004) were compared to crime in the active intervention period (December 22, 2004 to February 8, 2005) and, separately, postintervention (February 9, 2005 to March 29, 2005). The effects of the intervention were analyzed using a phi calculation and the percentage change in crime. Crime displacement in the buffer area surrounding the target zone was assessed using the Weighted Displacement Quotient (WDQ).

When comparing crime prior to the intervention to the active intervention period, crime trends favored the control group for vehicle, violent, and drug crime whereas burglary trends favored treatment (Ratcliffe & Breen, 2011). Overall, the target area experienced a 24% increase in total crime and the control area experienced an 18% decline. All types of crime decreased in the buffer area but the WDQ indicated the presence of displacement for violent and drug crime and a diffusion of benefits for vehicle crime and burglary. Results were more supportive of the intervention when a pre‐post comparison was used as trends favored treatment for all four outcomes. For all four outcomes combined, the target area experienced a 44% reduction in total crime whereas the control group experienced an 8% increase. Additionally, the WDQ showed evidence of a diffusion of benefits for violent crime, burglary, and drug crime but displacement effects for vehicle crime.

### Predictive Risk Mapping and Policing in Trafford, England

The Trafford Basic Command Unit in the Greater Manchester Police conducted a directed patrol strategy using predictive policing that weighed spatial and temporal risks of domestic burglary revictimization (Fielding & Jones, 2012). Risk levels were communicated to officers on a map using a color‐coded scheme. Fielding and Jones (2012) used a quasiexperimental design with two different comparison groups to examine the effects of the strategy: areas similar to the target zone within the Greater Manchester area and, separately, nationwide. Burglary counts during the intervention (May 12, 2010 to May 10, 2011) were compared to counts in the previous year. Although the treatment targeted specific micro‐time hot spots, the analysis was completed at the police division level.

A time series analysis with a first‐order autoregressive specification was used to estimate the impact of the intervention (Fielding & Jones, 2012). Domestic burglary declined in both the treatment and control areas during the intervention compared to the previous year, but this decrease was only significant in the targeted areas. Additionally, the target zones identified as most at‐risk for revictimization experienced the greatest crime control gains with decreases in domestic burglaries from 45% to 53%.

### Broken Windows Style Crackdowns in Three Cities in California

Despite the effectiveness of hot spots policing at reducing crime and disorder, the strategy (particularly broken windows policing) has been criticized for potentially generating “backfire effects” that harm communities (Weisburd et al., 2012; see also Weisburd et al., 2011). Weisburd et al. (2012) empirically investigated this possibility by analyzing the effects of a 6‐month broken windows hot spot policing initiative in three California cities (Redlands, Colton, and Ontario) on citizen attitudes (fear of crime, police legitimacy, and collective efficacy) and crime. Officers in the treatment condition received a one‐day training on broken windows policing, were encouraged to never ignore incidents of disorder, and received guidance on how to how respond when disorder was encountered. Furthermore, treatment segments received three additional hours of patrol per week; during these hours, officers specifically focused on addressing social and physical disorder. A block randomized experimental design was used to randomly assign 110 high crime street segments to treatment (*N* = 55) and control conditions (*N* = 55). Attitudinal measures examined in this study were obtained from a two wave panel survey (pre‐post) of persons living or working in the identified hot spots who completed the pre‐ and postintervention survey (*N* = 371).

Through a series of ANOVA models that included controls for the city and an interaction between the intervention and city, Weisburd et al. (2012) found scant evidence suggesting that broken windows policing at crime hot spots negatively impacted residents' attitudes toward crime or law enforcement. Residents in treated hot spots did not report being more fearful of crime (perceived risk and fear of walking alone at night) or experience changes in collective efficacy. Individuals exposed to treatment perceived lower levels of crime than those in the control group, though this difference was not statistically significant. Additionally, no significant differences were present between treatment and control subjects for perceptions of disorder. Analyses of calls for service data did not indicate that the hot spot experiment was associated with reductions in crime (Weisburd et al., 2012).

### Operation LASER in Los Angeles, California

In September 2011, the Newton Division of LAPD started Operation LASER (Los Angeles' Strategic Extraction and Restoration program) in an effort to reduce gun violence (Uchida & Swatt, 2013). Operation LASER was a dual‐pronged approach that deployed both offender‐focused and location‐based strategies at high crime areas within selected reporting districts. Uchida and Swatt (2013) analyzed reporting districts separately based on whether they received both the chronic offender and chronic location components of the intervention (*N* = 20) or whether they received only the chronic offender treatment (*N* = 19). The comparison group in this quasiexperimental evaluation was composed of 334 reporting districts drawn from the seven police divisions neighboring the target division. Hierarchical linear modeling was used to estimate the impact of the intervention where crime counts for each month were nested by reporting district. All gun‐involved Part I and Part II incidents in the preintervention period (January 2006 to August 2011) were compared to crime in the active period (September 2011 to June 2012).

Overall, Operation LASER was associated with a statistically significant 5% reduction in monthly gun crime (Uchida & Swatt, 2013). The effectiveness of the intervention varied by the type of treatment that was delivered. Reporting districts that received both the chronic offender and chronic location treatments experienced a significant 7% decrease in gun crime whereas no significant changes in gun crime were observed in reporting districts that only received the chronic offender treatment.

### Palos Verdes Team Policing Project in Las Vegas (NV)

Launched in March 2012, the Palos Verdes Team Policing Project was a problem‐oriented policing initiative that sought to reduce violent, property, disorder, and total calls for service in a high crime neighborhood in Las Vegas, Nevada (Martinez, 2013). The initiative also sought to repair strained police–community relations in the target area and build pride and a sense of ownership among neighborhood residents. A team of nine law enforcement personnel under the supervision of a sergeant and lieutenant were responsible for developing and implementing the initiative. The intervention primarily relied on saturation patrol and offender‐focused investigations but also used other tactics such as public health inspections, organized beautification and graffiti removal efforts, and community outreach. Martinez (2013) evaluated this initiative using a quasiexperimental design with the single treatment neighborhood matched to three comparison neighborhoods of a similar size, demographic composition, and level of calls for service. Paired sample *t* tests were used to compare changes in mean weekly calls for service during the intervention (April 2012 to December 2012) to the 9 months immediately preceding the intervention and, separately, the same 9 months the previous year to account for seasonality. Calls for service in a catchment area adjacent to the targeted neighborhood was examined for displacement effects.

Results reported in this evaluation were consistent regardless of the preintervention period referenced. The treatment neighborhood experienced significant increases in violent and total calls for service but there were no noteworthy changes in property and disorder offenses (Martinez, 2013). When accounting for seasonality, crime in the three comparison areas largely remained unchanged. Lastly, there was no evidence of spatial crime displacement.

### License Plate Readers at Crime Hot Spots in Mesa (AZ)

Koper et al. (2013) examined the effects of a short‐term police patrol deployment strategy using license plate readers (LPR) on violent crime, property crime, drug offenses, disorder, and auto theft in Mesa, Arizona. Four officers in Mesa PD's specialized vehicle theft unit each received a LPR device and were directed to high‐risk roadway segments on a rotating basis. Each route receiving treatment experienced LPR patrol for 1 hr/day for 8 days out of a 2‐week period before the next route is targeted. LPR operations were carried out Wednesday through Saturday from 3 p.m. to 1 a.m. To explore whether LPR technology enhanced crime reduction effectiveness, a second treatment condition was introduced that assigned officers to hot spots where they would conduct manual license plate checks.

The intervention took place for a 30‐week period from August 2008 to March 2009 (Koper et al., 2013). A block randomized controlled design was used to assign 117 high‐risk routes to treatment and control conditions: 45 routes received the LPR‐enhanced patrol, 45 received increased patrol with manual checks, and 27 served as the control group. The impact of the intervention was estimated using count‐based random effects panel regression models that controlled for seasonality. Both short‐ and long‐term treatment effects were considered as preintervention calls for service were compared to both when the treatment was active and in the 2‐week posttreatment period. Displacement was assessed by analyzing changes in crime levels in routes adjacent to those receiving treatment.

The effectiveness of the intervention varied by the tactic used and follow‐up period considered. Overall, drug crime declined significantly by 28% among routes receiving the LPR‐enhanced treatment whereas manual check treatment routes experienced a significant 35% increase in calls for drug crime (Koper et al., 2013). When focusing on only the period when the treatments were active, neither the LPR‐enhanced or manual check treatment groups were significantly related to any of the five outcomes considered. In the 2 weeks following the intervention, LPR treatment areas experienced a significant 49% decrease in drug calls and manual check treatment routes experienced significant declines of 75% for auto theft calls and 46% for person crimes. Evidence of short‐term crime displacement was present: routes adjacent to those that were treated experienced significant increases in person and disorder crime that lasted 2 weeks after the treatment was administered.

### Lowell (MA) Smart Policing Initiative

From 2007 through 2008, Lowell, Massachusetts experienced a substantial increase in property crime that was largely driven by drug‐motivated offenders. In response to this growing problem, the Lowell Smart Policing Initiative began in September 2011 and utilized a problem‐oriented policing approach to target drug‐related property crime (Bond et al., 2014). Specific strategies that were used varied by sector but common tactics included directed patrol, traffic enforcement, community engagement, and targeting prostitution. Bond et al. (2014) evaluated the intervention using a quasiexperimental design. Sector captains in three districts each selected four hot spots in their sector; then, each hot spot was matched to a comparison hot spot that had similar crime and social characteristics. The impact of the intervention was estimated using a simple percentage change in property crime preintervention period (September 2009 to December 2010) compared to the active intervention (December 2012). Results were aggregated by sector.

Changes in crime from the preintervention period to the active intervention period favored the treatment condition (Bond et al., 2014). In all three sectors, treatment hot spots experienced a decrease in property crime that was greater than comparison hot spots. Treatment hot spots experienced reductions in property crime of 19% (North Sector) and 16% (East and West Sectors). Comparison hot spots experienced decreases in property crime of 14% (North Sector) and 7% (East Sector), and an increase of 5% (West Sector).

### DDACTS Program in Shawnee (KS)

The Shawnee Police Department carried out a 3‐year DDACTS initiative in an effort to reduce instances of vehicle burglary, vehicle theft, and robbery (Bryant et al., 2014). Principal tactics deployed during this intervention were increased police presence and traffic enforcement in the target zone. Bryant et al. (2014) evaluated the intervention using a mixed methods approach that included focus groups with law enforcement personnel, a business survey, a community survey, and a quantitative impact assessment. The impact assessment followed a quasiexperimental design with the treatment zone compared to two separate control areas: (a) a zone with a comparable population size, land use, and target crime levels and (b) the remainder of the city. *t* tests comparing mean crime 3 years before and 3 years during the intervention for the target zone, control zone, and remainder of the city were used to assess the impact of the intervention. Spatial displacement effects were explored by analyzing crime in the city adjacent to Shawnee.

Qualitative analyses demonstrated support for the intervention among police, businesses, and residents. Focus groups conducted with officers revealed a shift in culture and increased officer “buy in” over the course of the initiative (Bryant et al., 2014). Officers indicated that they considered DDACTS an effective and sustainable crime reduction strategy. Participants in the business and resident surveys perceived a greater police presence and higher number of traffic stops during the intervention. They also reported that quality of life in Shawnee improved as a result of DDACTS and they were supportive of high‐visibility targeted traffic enforcement.

Results from the quantitative impact assessment were also positive and supportive of the intervention. The target zone experienced a statistically significant 40% reduction in total target crimes and a marginally significant 70% reduction in robbery (Bryant et al., 2014). Vehicle burglary, vehicle theft, and collisions all decreased during the intervention compared to the preintervention period but not at a statistically significant degree. In contrast, there were no significant reductions in any of the outcomes in the control zone and changes in crime in the remainder of the city were inconsistent and varied by the type of offense. When disaggregating the treatment components, there was no statistically significant correlation between the intensity of enforcement activities and any of the crime outcomes examined. Evidence of a spatial diffusion of benefits was present for vehicle theft and total targeted crime.

### Summer Crime Initiative in Washington, DC

In 2011, the Metropolitan Police Department in Washington, DC launched a location‐based arrest‐driven crackdown known as the “Summer Crime Initiative” (SCI). The SCI used targeted enforcement with the goal of reducing violent crime, gun‐related offenses, and drug‐related crime (Mazeika, 2014). The treatment consisted of a team of officers deployed to hot spots for 24 hr a day for 7 days per week using 12‐hr rotational shifts. Mazeika (2014) used a quasiexperimental difference‐in‐difference research design that compared five treatment hot spots to five control hot spots with similar crime rates, arrest rates, and demographics. Citizen‐generated calls for service for robbery in the preintervention period were compared to both the active and postintervention periods for both treatment and control groups. General displacement was assessed by examining crime in two‐block buffer zones around target and control locations. Specific displacement was explored by tracking a cohort of 475 offenders who were arrested for robbery in the 16 months prior to the intervention and whether they were rearrested.

Compared to the preintervention period, citizen‐generated robbery calls for service during the intervention decreased significantly in target areas and increased in control areas (Mazeika, 2014). When comparing the postintervention period to the active intervention period, robbery increased in the treatment area and decreased in the control area. There was little evidence of general crime displacement into areas surrounding the treatment area. As for specific displacement, more offenders in the control cohort were rearrested than offenders in the treatment cohort during the intervention but this trend reversed in the postintervention period; however, differences between the two groups were not statistically significant.

### Operation Impact in Newark (NJ)

Operation Impact was a place‐based crime reduction strategy that began in June 2008 in Newark, New Jersey (Piza & O'Hara, 2014). Closely resembling the Operation Impact that was carried out in New York City, this iteration of the program was primarily a saturation patrol strategy where rookie officers were assigned to patrol the targeted area on foot from 6 p.m. to 2 a.m. in an effort to reduce total violent crime, murder, shootings, nondomestic aggravated assault, and robbery (Piza & O'Hara, 2014). The target zone was a mix of apartment buildings and businesses, with a large 28‐building low‐rise housing complex known for drug trafficking fixated at the eastern portion of the zone. A quasiexperimental design was used to analyze crime in the target area relative to two different comparison groups: (a) another zone with similar crime problems and land use and (b) the rest of the precinct (minus the target and catchment areas). Piza and O'Hara (2014) estimated the impact of the intervention using ORs that compared crime in the treatment and control areas preintervention (June 4, 2007 to June 3, 2008) and during the intervention (June 4, 2008 to June 3, 2009). The intervention was active for 2 years but Piza and O'Hara (2014) noted that declines in the department's budget and personnel weakened the intervention over its final year. Tests for spatial displacement effects considered crime in the 1‐block area surrounding the target area. Temporal displacement was analyzed by examining crime that occurred outside the intervention's operational hours.

For all outcomes considered, crime reductions favored treatment over the precinct comparison group (Piza & O'Hara, 2014). These declines were statistically significant for overall violence, aggravated assault, and shootings. Analyses using the similar zone as the comparison group were consistent with findings using the precinct for comparison. Crime during nonoperational hours for four of the five outcomes examined suggesting a temporal diffusion of benefits, with the exception being robbery which increased 73% during nonoperational hours. There was also evidence of a spatial diffusion of benefits for overall violence, aggravated assault, and shootings, but evidence of displacement for robbery.

### St. Louis (MO) Metropolitan PD's Firearms Violence Hot Spots Experiment

Faced with problems of serious gun violence, St. Louis Metropolitan PD launched an experiment testing the effectiveness of two different patrol strategies: directed patrol with self‐initiated enforcement and directed patrol without self‐initiated enforcement (Rosenfeld et al., 2014). Deployments for both types of patrol were limited to times when the gun violence was most prevalent (3 p.m. to 7 a.m.). The experiment was originally planned to last 3 months but was extended to 9 months. A randomized controlled trial stratified by police district was used to examine the impact of these two strategies on rates of nondomestic gun assault and gun‐involved robbery. Of the total sample of 32 identified hot spots, eight were assigned to treatment condition #1 (directed patrol without self‐initiated enforcement), eight to treatment condition #2 (directed patrol with self‐initiated enforcement), and eight to the control group. There were 258 total street segments within the 32 hot spots, with an average of eight street segments per hot spot. Rosenfeld et al. (2014) evaluated the intervention using a multilevel linear model that compared crime in the 9‐month intervention period to crime in the preceding 9 months. Spatial displacement was assessed by examining crime rates within a 500‐ft radius of the identified hot spots. Temporal displacement was assessed by examining crime during the daytime shift when treatment was not active. Lastly, offense displacement was assessed by examining nonfirearm assault.

Analyses revealed differing levels of effectiveness between the two types of treatment. Directed patrol with self‐initiated enforcement was associated with significant reductions in firearm assault rates and marginally significantly declines in overall firearm violence but had no effect on gun‐involved robbery (Rosenfeld et al., 2014). Directed patrol without self‐initiated activity was not significantly related to any of the three outcomes examined. Further analyses of directed patrol with self‐initiated activity and nondomestic firearm assault explored the effects of the amount of self‐initiated activity and directed patrol were analyzed separately. Greater amounts of self‐initiated activity were significantly related to lower firearm assault rates whereas the amount of directed patrol yielded no significant effects. When the type of self‐initiated activity was disaggregated, arrests and occupied vehicle checks were the only two types of activity significantly related to lower rates of firearm assaults. Lastly, no evidence was found indicating the presence of displacement in any of the forms examined (spatial, temporal, or offense). Importantly, Rosenfeld et al. (2014) noted that although the directed patrol with self‐initiated activity treatment was implemented with strong fidelity, the fidelity for directed patrol without self‐initiated activity was limited. Because officers in the latter group were instructed to limit their self‐initiated activity, more self‐initiated activity should have been observed in the control group but the opposite occurred.

### Hot Spots Randomized Field Trial in Sacramento (CA)

After several personnel layoffs, Sacramento PD sought an efficient way to allocate resources and reduce crime. This desire was manifested in the form of a 90‐day directed patrol strategy without additional outside funding (Telep et al., 2014). In an effort to reduce citizen‐initiated calls for service, Part I crime incidents, and soft crime (e.g., disorder), patrol officers were assigned 1 to 6 crime hot spots in a random order that they were to visit for 12 to 16 min each at least once every 2 hr, and officers' patrol patterns were monitored using data from an automated vehicle locator system. The effectiveness of this directed patrol strategy was assessed using a block randomized controlled trial design where 42 eligible hot spots were randomly assigned to treatment (*N* = 21) and control conditions (*N *= 21). *t* tests were used to compare outcomes in the experimental period to the same period in the previous year (2010) and, separately, the average of the previous years (2008 to 2010).

Difference‐in‐difference results revealed that treatment hot spots experienced significantly fewer calls for service than control hot spots regardless of the preintervention period referenced (Telep et al., 2014). For Part I incidents, there were significant decreases in crime favoring the treatment condition relative to the control condition but only when using 2010 alone as the comparison time period whereas no effects were observed for soft crime incidents. Overlapping catchment areas surrounding identified hot spots limited analyses of crime displacement to 11 treatment and 9 control hot spots. Overall, there was evidence of slight displacement effects. Catchment areas surrounding treated hot spots experienced marginally significant increases in calls for service and Part I incidents compared to control catchment areas. Mitchell (2017) completed a cost‐benefit analysis of the Sacramento Hot Spot Experiment and found the experiment was associated with more modest benefits when measuring effects using the California Crime Harm Index instead of crime counts, and that most harm reduction associated with the intervention resulted from changes in property crime.^21^

### Trinidad and Tobago Police Services Hot Spot Experiment

Trinidad and Tobago Police Services launched a directed patrol initiative in 2013 aimed at reducing violent crime (murder, wounding, and shootings) (Sherman et al., 2014). Directed patrol treatment was delivered at identified hot spots within the targeted districts. The effectiveness of the direct patrol strategy was tested using a randomized controlled trial design where police districts were randomly assigned to treatment (*N *= 20) and control conditions (*N *= 20). Personnel in treatment districts held meetings every 2 weeks to review the previous 14 days of violent crime incidents and to offer feedback to patrol officers. Sherman et al. (2014) assessed the impact of the intervention using a meta‐analysis of the 20 treatment‐control pairs to calculate an overall effect size that compared violent crime before (September 2012 to August 2013) and after the intervention (December 2013 to May 2014).

Overall, results from the meta‐analysis of the 20 pairs of hot spots demonstrated that the intervention was associated with a small but statistically significant decrease in murders and shootings (Sherman et al., 2014). Reductions in violent crime favored treatment for 15 of the 20 district pairs. And, of these 15 district pairs, crime reductions in four were statistically significant (*p* < .05) and two were marginally significant (*p* < .10).

### Policing Crime Hot Spots in Stockholm, Sweden

Stockholm, Sweden was challenged by muggings (robberies) that occurred most frequently late in the evening and at night, and were commonly carried out by groups of young men (Marklund & Merenius, 2014). Violence occurred in approximately 60% of muggings and threats with a weapon happened in approximately one‐third. To address rampant muggings, a problem‐oriented policing initiative was implemented in October 2012 at seven mugging hot spots by a special committee of investigators and patrol officers. Directed patrol at crime hot spots on days and at times when muggings were most frequently committed was the primary crime reduction tactic used but the committee also placed greater emphasis on comprehensive investigations, crime pattern analysis, focusing on repeat offenders, making contact with potential offenders and victims, and speaking with buyers and sellers of mobile phones (which were frequently taken in muggings). Over the course of the intervention, there were an average of six visits to each hot spot per shift, with each visit averaging approximately 25 min. Marklund and Merenius (2014) examined the effects of this intervention using a quasiexperimental design with the remainder of the inner city as the comparison group. A series of *t* tests were used to assess differences between the average weekly crime before and during the intervention, with a subgroup analysis of nights when directed patrols were and were not active.

Results did not reveal crime reduction effects in favor of treatment (Marklund & Merenius, 2014). There were 0.7 fewer robberies in the treatment areas per week during than intervention compared to the previous year but this decline was nonsignificant and smaller than the robbery decline in the rest of the city. Analyses that focused on nights when directed patrols were and were not active also did not suggest the presence of a treatment effect. Both treatment and control groups experienced nonsignificant 7% declines in robberies per day when direct patrols were active compared to the previous year. On nights when directed patrols were not active, robbery reductions were greater in the control location (11%) than the treatment location (9%).

Marklund and Merenius (2014), however, did note several implementation challenges that compromised treatment integrity. The dosage of directed patrol treatment at hot spots during times and days when muggings most frequently occurred was relatively low due to officer contracts that only allows them to work 4 weekend nights within a 6‐week period. Furthermore, officers engaged in directed patrol made fewer contacts with repeat robbery suspects the longer the intervention was enacted and results of the investigations did not improve. Lastly, on numerous occasions, resources were not ready or available to initiate patrols.

### Policing Crime Hot Spots in Eskilstuna, Sweden

In September 2012, police in Eskilstuna, Sweden implemented a problem‐oriented strategy in response to the increasing problem of public assaults (Markland & Merenius, 2014). These assaults most frequently occurred on weekend nights and many were alcohol‐fueled, took place outside of a pub or night club, and involved young men. Law enforcement collaborated with owners and employees of bars where assaults frequently occurred. Police also sought to reduce instances of over‐serving alcohol via undercover operations and increased monitoring of security guards working at the bars. Notably, no additional funds were used to support policing activities carried out during this intervention. Marklund and Merenius (2014) used a quasiexperimental design to examine the effects of the intervention. Weekly crime in the three hot spots that received treatment was compared to the remainder of the city before and during the intervention.

Analyses revealed results that favored the intervention. Abuse and violence against officials decreased 16% during the intervention compared with the previous year whereas no change in crime occurred in the rest of the city (Marklund & Merenius, 2014). For the times and days that were the primary focus of the intervention, treatment hot spots experienced a 28% decline in the targeted crimes whereas the rest of the city experienced an 8% increase.

### Anti‐Drunk Driving Program in Rajasthan, India

In 2010, the Rajasthan police department launched cracking crackdown on drunk driving in an effort to determine the most efficient way to deploy its finite resources (Banerjee et al., 2014). Banerjee et al. (2014) proposed a theoretical model where offenders are considered active learners who strategically modify their offending behavior based on patterns of police deployments. Treatment in this experiment was randomized in two ways. First, target areas were randomly assigned to have their treatment randomly distributed across three routes or to have it fixed at the route with the highest crime. Second, the intensity of treatment was randomized. The effects of varying methods of conducting vehicle checkpoints on drunk driving was evaluated using a multilevel, fixed‐effects Poisson regression model (Banerjee et al., 2014).

Results indicated that surprise checkpoints were an effective way of reducing drunk driving and nighttime fatal car accidents (Banerjee et al., 2014). Randomly rotating where vehicle checkpoints were conducted yielded greater benefits than static deployment of vehicle checkpoints. These effects were sustained for up to 6 weeks following the crackdown. Collectively, these results support the proposition that people learn the methods and locations of police crackdowns and strategically respond to it by taking alternative routes.

### Philadelphia (PA) Policing Tactics Experiment

The Philadelphia Policing Tactics Experiment utilized a stratified randomized controlled trial design to examine the effects of three separate hot spots policing tactics (foot patrol, problem‐oriented policing, and offender‐focused policing) on violent crime (homicide, robbery, aggravated assault, and simple assault) and violent felonies (homicide, robbery, and aggravated assault) (Groff et al., 2015). Groff et al. (2015) evaluated the impact of the three strategies using a longitudinal multilevel design with negative binomial regression. Treatment was modeled using a contrast coding scheme that only compared treatment areas to control areas “within each tactic's randomization pool during treatment time periods but it ignores differences between the treatment and control areas for a respective tactic during nontreatment time periods and never contrasts the treatment and control areas across randomization pools during any time periods” (Groff et al., 2015, p. 36). Spatial displacement was assessed by examining crime in buffer areas surrounding the identified hot spots.

A total of 81 hot spots were initially identified then police commanders classified them based on how suited they were for the three different types of treatment (Groff et al., 2015). For each grouping, 20 hot spots were randomly assigned to the treatment condition and seven to the control condition. The foot patrol treatment condition consisted of “a minimum of 8 hours per day, 5 days per week, for 12 weeks” with District Captains determining how many officers and what times and days the hot spots would be patrolled. The POP treatment condition involved teams of district officers who received training on problem‐oriented policing and collaborated with community members following the SARA model. Lastly, the offender‐focused approach identified repeat violent offenders residing or engaging in violent crime in the target area who then received extra attention from law enforcement (e.g., aggressive patrol and partnerships with beat officers). Start dates varied across and within treatment conditions, and all hot spots received 12 to 24 weeks of treatment. This staggered implementation was not intentional. The original plan was for all interventions to start and end at the same time (June 2010 to August 2010) to minimize threats linked to seasonality but it took longer for the offender‐focused and POP tactics to become operational compared with the directed patrol tactic.

Of the three different treatment conditions that were tested, only the offender‐focused strategy was associated with any crime control gains (Groff et al., 2015). Specifically, the offender‐focused strategy was related to a 42% decrease in all violent crime and a 50% decrease in violent felonies relative to the control group. Because neither the foot patrol or POP strategies were associated with either outcome, crime displacement analyses were limited to the offender‐focused tactic. For both violent crime and violent street felonies, evidence suggests a diffusion of benefits associated with the treatment.

### Colorado Springs PD's Risk‐Based Intervention in Colorado Springs (CO)

Colorado Springs PD's risk‐based intervention was part of a broader initiative examining the effectiveness of risk terrain modeling in five U.S. cities and sought to mitigate incidents of disorder and motor vehicle theft (Kennedy et al., 2015). Tactics that were utilized in this intervention included “Code Enforcement property inspections, Community Service Officer Neighborhood Cleanups, Community Meetings, Proactive Police Enforcement against disorder offenses, Proactive Traffic Enforcement, and the deployment of License Plate Recognition (LPR) devices for the purpose of identifying stolen Motor Vehicles in the target area” (p. 4). The intervention was evaluated using a quasiexperimental design with comparison groups constructed via propensity score matching: 144 street units received treatment and 144 street units served as controls. The impact assessment considered the Average Treatment Effect on the Treated as well as the effects of individual intervention tactics using regression analysis. Crime in the preintervention period (August 16, 2013 to November 30, 2013) was compared to crime in the postintervention period (August 16, 2014 to November 30, 2014) for both the treatment and control areas in order to account for seasonality. Potential spatial crime displacement was tested for by examining crime in catchment zones surrounding targeted areas.

An examination of treatment fidelity revealed that 97% of treatment activities occurred within the targeted area (Kennedy et al., 2015). Results suggested that the target area experienced a marginally significant 33% decrease in motor vehicle theft compared to the control condition. Furthermore, evidence indicated the presence of a diffusion of benefits as motor vehicle theft decreased in catchment zones surrounding treatment sites. When intervention tactics were disaggregated, code enforcement was the only tactic significantly associated with reductions in motor vehicle theft in the treatment area overall or at specific high‐risk places.

### Newark PD's Risk‐Based Intervention in Newark (NJ)

Newark PD's risk‐based intervention was part of a broader initiative examining the effectiveness of risk terrain modeling in five U.S. cities and sought to mitigate incidents of gun violence (Kennedy et al., 2015). The strategy implemented by Newark PD prioritized law enforcement making contacts with three types of businesses that were identified as being at an elevated risk for gun violence: restaurants, food take outs, and gas stations. A task force of three officers and a Lieutenant visited each of the identified businesses in the target each day that the intervention was active. Kennedy et al. (2015) evaluated the intervention using a quasiexperimental design with comparison groups constructed via propensity score matching. There were 177 street units that received treatment and 180 street units that served as controls. The impact assessment considered the Average Treatment Effect on the Treated as well as the effects of individual intervention tactics using regression analysis. Crime in the 3‐month postintervention period (February 10, 2014 to May 11, 2014) was compared to crime during the same period in the previous year (February 10, 2013 to May 11, 2013) for both the treatment and control areas in order to account for seasonality. Potential spatial crime displacement was tested for by examining crime in catchment zones surrounding targeted areas.

Kennedy et al. (2015) analysis of implementation fidelity found that roughly 97% of the treatment activities occurred within the targeted area. Results indicated that the target area experienced a marginally significant 35% reduction in gun violence. Furthermore, there was evidence of a slight diffusion of crime reduction benefits in areas surrounding the targeted areas. When considering individual aspects of the treatment, code enforcement was associated with significantly lower gun violence overall and at specific high‐risk areas. In contrast, CSO‐community meetings, proactive enforcement, and traffic enforcement, separately, were all unrelated to gun violence.

### Kansas City PD's Risk‐Based Intervention in Kansas City (MO)

Kansas City PD's risk‐based intervention was part of a broader initiative examining the effectiveness of risk terrain modeling in five U.S. cities and sought to mitigate incidents of aggravated violence (specifically, fatal and nonfatal shootings, aggravated assault with a firearm, and armed and unarmed street robbery) (Kennedy et al., 2015). Multiple tactics were deployed throughout the intervention including “Code Enforcement, Directed Patrols, Licensing and Inspection checks, meet‐and‐greets with known offenders juxtaposed with social service referrals/support, CPTED inspections, Pedestrian Checks, Area Presence, Residence Checks, Traffic Violations, and Building Checks” along with a new protocol for dispatching officers to certain calls for service. The evaluation of the intervention followed a quasiexperimental design with comparison groups constructed via propensity score matching; 139 treated street units were matched to 195 comparison street units. Regression analyses estimating the impact of the intervention considered the Average Treatment Effect on the Treated as well as the effects of individual intervention tactics. Crime in the active intervention and 3‐months postintervention was compared to crime during the same time periods in the previous year for both the treatment and control areas.

An examination of treatment fidelity showed that roughly 99% of treatment activities occurred within the targeted areas (Kennedy et al., 2015). When comparing the active intervention period to the preintervention period, the target area experienced a nonsignificant increase in aggravated violence. A comparison of pre‐post periods indicated a nonsignificant 12% decrease in aggravated violence among treated street units. Results pertaining to the effectiveness of police tactics individually varied by the level of analysis and time periods examined. For the during intervention analysis, pedestrian checks, area presence, and residence checks were each associated with significant reductions in aggravated violence for the collective target area but no individual tactic had a significant impact on crime at the high‐risk street unit level. For the postintervention analysis, no individual tactic was significantly related to aggravated violence in the target area overall but building checks were significantly related to lower violence in high‐risk areas.

### Glendale PD's Risk‐Based Intervention in Glendale (AZ)

Glendale PD's risk‐based intervention was part of a broader initiative examining the effectiveness of risk terrain modeling in five U.S. cities and sought to mitigate incidents of robbery (Kennedy et al., 2015). This initiative utilized a variety of tactics including “Directed Patrols, Flyer Distribution, Community Meetings and Engagement Activities, Proactive Stops, and Proactive Arrests” (p. 14). Whether this intervention was an effective approach to reducing robbery was explored using a quasiexperimental design with comparison groups constructed via propensity score matching. There were 37 street units that received treatment and 141 street units that served as controls. The impact assessment considered the Average Treatment Effect on the Treated and the effects of individual intervention tactics using regression analysis. Crime in the active intervention and postintervention periods was compared with crime during the same time periods in the previous year preintervention for both the treatment and control areas. Possible spatial displacement effects were considered by examining crime in catchment zones surrounding targeted areas.

An assessment of treatment fidelity revealed that 91% of treatment activities occurred within the targeted area (Kennedy et al., 2015). Because of the spillover of treatment outside of the targeted area, Kennedy et al. (2015) defined treated areas as street units that received at least one intervention action. When comparing the active intervention period to the preintervention period, treatment areas experienced a marginally significant 42% decrease in robbery relative to control areas. Moreover, results indicated that diffusion of benefits occurred in catchment areas surrounding the targeted locations and that crime control gains were greater in catchment areas than street units that were treated directly. A pre‐post analysis indicated that the treatment group experienced a nonsignificant 38% increase in robbery over the comparison group. Testing for the effects of individual tactics found limited evidence suggesting directed patrol and flyer distribution were associated with reductions in hot spots overall but no individual tactic was related to robbery when focusing solely on high‐risk street segments.

### St. Louis County (MO) Hot Spots in Residential Areas Study

The St. Louis County Hot Spots in Residential Areas Study tested the effects of two different hot spot policing strategies (problem‐solving and directed patrol) on calls for service (Kochel et al., 2015). Because residential areas were the focus of this intervention, hot spots were required to have at least 40 residential address to be eligible for the study. After initial identification, potential hot spots were then vetted by precinct commanders. A stratified randomized controlled experimental design was used to assign 71 hot spots to treatment and control conditions: 20 received the problem‐solving treatment, 20 received the directed patrol treatment, and 31 served as the standard policing practice control group. The problem‐solving treatment consisted of 22 officers trained on the SARA method who partnered with at least one community stakeholder to identify and respond to a problem (the majority of which ended up being property crime). The directed patrol treatment sought to double the amount of time officers spent at each hot spot. Automated vehicle location data were used to track the treatment fidelity of the directed patrol effort and found time spent at hot spots increased during the study period “except for the last 2 weeks of the project, at which point officer fatigue with the treatment prevailed” (p. 5).

The evaluation of St. Louis County Hot Spots in Residential Areas Study used crime data as well as community survey data. An ARIMA model with controls for seasonality was used to quantify the impact of the intervention on calls for service before and after the intervention was initiated (Kochel et al., 2015). Results indicated that directed patrol hot spots and problem‐solving hot spots experienced statistically significant decreases of 5% and 7%, respectively, in calls for service. The control group experienced a decrease in calls for service but not at a statistically significant level.

The impact of the intervention was also considered in terms of its effects on community perceptions of law enforcement. Specifically, Kochel and Weisburd (2017) used mixed effects regression analyses to analyze whether the intervention influenced residents' perceptions of police abuse, procedural justice and trust, police legitimacy, and willingness to cooperate with police. Analyses revealed that residents subjected to the directed patrol treatment experienced significant short‐term decreases in procedural justice and trust compared to the control group, and a nonsignificant decrease in police legitimacy. Conversely, residents subjected to the problem‐solving treatment experienced no significant short‐term attitudinal changes pertaining to law enforcement. In the long term, residents in hot spots that received either treatment reported being more likely to cooperate with police.

### Mobile Computing Technology at Crime Hot Spots in a Suburban County

Koper et al. (2015) explored whether mobile computing technology assisted officers in reducing crime at hot spots in a high crime suburban police district. A block randomized controlled trial design was used to assign 18 hot spots to the treatment (*N *= 9) and control conditions (*N *= 9) in a suburban jurisdiction (Koper et al., 2015). The impact of the intervention was analyzed using a longitudinal panel design with a negative binomial count distribution and a lagged time measure to account for seasonality. Notably, the extent to which officers used mobile IT was not randomized but it was considered in subgroup analyses.

Koper et al. (2015) stressed that the main factor driving the need for subgroup analyses was a threat to treatment integrity. Officers were instructed to carry out three 15‐ to 30‐min patrols per shift at their assigned hot spot; however, due to officer discretion and resources limitations, the actual treatment delivered at hot spots was considerably lower than what was originally intended. The average time per visit spent by officers on a visit to a hot spot was within the intended range (26 min) but each hot spot only received less than two visits per week over the 11‐week intervention period. Additionally, how officers used the technology differed from what was expected. Specifically, officers tended to use the mobile computing technology for traditional and reactive purposes rather than for strategic problem‐solving and crime prevention.

Quantitative analyses revealed locations that received the additional patrols with mobile computing technology experienced a nonsignificant 11% decrease in crime incidents (Koper et al., 2015). When disaggregating treatment by the intensity of dosage, high dosage experimental areas experienced a marginally significant 24% crime reduction whereas crime in low dosage treatment sites increased slightly. An additional subgroup analysis performed by Koper et al. (2015) cross‐referenced the level of patrol dosage (low or high) with the level of technology use (low or high). High dosage patrol sites with low IT use experienced a marginally significant 45% crime decrease and high dosage sites with high IT use experienced a significant 14% reduction in crime. In contrast, low dosage patrol sites were not significantly related to crime regardless of the level of technology use.

### Proactive CCTV Monitoring with Directed Police Patrol in Newark (NJ)

In 2007, Newark PD began installing CCTV cameras and eventually had 146 CCTV cameras placed throughout the city (Piza et al., 2015). However, limited resources were dedicated to camera monitoring and incidents that were observed by monitors were not often reported because of large queues in calls for service (Piza, Caplan, and Kennedy 2017).^22^ In an effort to improve how CCTV cameras were monitored, Newark PD –conducted an experiment involving proactive camera monitoring. Additional staff were assigned to monitor cameras and two patrol cars could be directed to particular areas when monitors observed an incident occurring. A randomized controlled trial design was used to evaluate whether proactive CCTV monitoring was an effective means of reducing violent crime, social disorder, and narcotics activity (Piza et al., 2015). Nineteen sites were randomly assigned to treatment and 19 sites to the control condition. Treatment was modeled in three different ways: (a) tours when the treatment was in effect (8 p.m. to 12 a.m.); (b) days that the treatment was active (Wednesday to Saturday); and (c) the entire 11‐week treatment period (July 20, 2011 to October 1, 2011). For all three treatment effects, negative binomial regression was used to compared preintervention calls for service to calls for service in the active intervention period. Displacement effects were explored by examining crime in buffer areas one median block (291 feet) surrounding each viewshed.

During tours when the treatment was active, the intervention was associated significant reductions of 48% for violent crime and 49% for social disorder (Piza et al., 2015). On days that the treatment was active, there was a significant 40% decline in favor of treatment areas relative to control areas. Over the full experimental area, social disorder in treatment areas decreased significantly by 41% compared to control areas; violent crime and narcotics activity also declined but not to a significant degree. In all three models, the intervention was not significantly related to narcotics activity. Results for crime displacement were mixed. Evidence of a diffusion of benefits was observed for violent crime but evidence suggested the presence of crime displacement for social disorder offenses during tours when the treatment was active. Over the full 11‐week period, there was a diffusion of benefits for social disorder greater than the reductions observed in the actual treatment areas. Further analyses found evidence of residual deterrence for narcotics activity but this was accompanied by crime displacement. Total net effects showed sizable declines in violent crime in favor of treatment during active tours and days, and a decrease in social disorder over the full 11‐week period.

### Tactical Police Response at Micro‐Time Hot Spots in Port St. Lucie (FL)

aaSantos and Santos (2015a, 2015b) evaluated a 5‐year period where Port St. Lucie PD embraced a tactical police response strategy aimed at micro‐time hot spots. It was established that three criteria must be met to be considered a micro‐time hot spot: “(1) two or more residential theft from vehicle crimes; (2) occurring from one to 14 days of another; (3) within a 0.5‐mile radius or 0.79 square miles” (Santos & Santos, 2015a, p. 684). Following the identification of a micro‐time hot spot, the crime analyst developed and published a one‐page bulletin containing information on the crime, suspects, known offenders in the area, field interview information, and whether evidence has been collected. Upon receiving this information, police targeted these micro‐time hot spots by conducting directed patrol, contacting potential victims, and contacting known offenders. Responses were carried out for 14 days after the micro‐time hot spot was identified but micro‐time hot spots were tracked until no crime occurred within the identified radius for 21 days.

The effectiveness of this approach was assessed using a quasiexperimental design with a comparison group constructed via propensity score matching. Separately, Santos and Santos (2015b) examined the effects of this same strategy on residential burglary. In both studies, impact assessments used independent *t* tests to measure the mean difference in crime between target and control areas posttreatment. However, samples size differed between the two studies. The residential theft from vehicle crime analysis consisted of 86 treatment and 86 control areas whereas the residential burglary analysis consisted of 54 treatment and 54 control areas. Potential displacement effects were assessed by examining crime in the 0.2‐mile catchment area within 14 days of the last crime. For both outcomes examined, directed patrol accounted for 68% to 76% of the response tactics deployed (Santos & Santos, 2015a, 2015b). Efforts targeting micro‐time hot spots were associated with significant reductions in residential theft from vehicle crime and residential burglary. Micro‐time hot spots that received a police response also “cooled off” quicker than those that were left untreated. Additionally, no evidence of spatial displacement was found for either outcome.

### Philadelphia (PA) GunStat Model

As part of the mayor's re‐election bid, and in response to the city's issues with violent crime, Philadelphia began a “collaborative initiative intended to address the problem of violence committed by recidivist offenders responsible for violent acts in violent crime hot spots” called GunStat (Sorg, 2015, p. 30). In short, the Philadelphia GunStat model was an offender‐focused approach that “(1) identifies violent crime hot spots on which to focus law enforcement efforts, (2) identifies prolific offenders responsible for these crimes at these places, (3) takes enforcement action at these individuals through various means and, (4) focuses on aggressively prosecuting and monitoring these offenders” (Sorg, 2015: pp. 30). The intervention was carried out in two phases: (a) January 1, 2012 to December 31, 2012 and (b) January 1, 2013 to December 31, 2013. Sorg (2015) evaluated the intervention using a quasiexperimental design with comparison groups constructed via three different types of propensity score matching (nearest neighbor, caliper matching, and optimal matching). Negative binomial regression was used to estimate the impact of the intervention with the size of the hot spot as a measure of exposure.

During Phase I of the intervention, violent crime and violent street felonies in target locations were substantially higher than crime in control locations (Sorg, 2015). Specifically, in the first year of the intervention, treatment locations experienced significantly higher violent crime (5% to 29%) and violent street felonies (6% to 64%) relative to control locations. For Phase II of the intervention, treatment was unrelated to total violent crime and violent street felonies. Sorg (2015) stressed that several threats to treatment integrity were encountered during the implementation of this strategy: the intervention faced challenges stemming from a lack of cross‐district collaboration, concerns that the Philadelphia PD intelligence division withheld information on certain offenders, politics over offender selection, and a lack of priority and enhanced monitoring given targeted offenders by the probation and parole department.

### Dallas (TX) Patrol Management Experiment

The Dallas Patrol Management Experiment examined the effects of using automatic vehicle location system on increasing the amount of directed patrol and reducing crime (Weisburd et al., 2015). For determining what areas would receive directed patrol, each division‐shift could identify up to five hot spots on a weekly basis; however, Weisburd et al. (2015) found that this five‐hot spot threshold was met in only 7% of cases. Treatment delivered at the beat level consisted of the research team delivering two reports to Division Commanders: (a) a list of the amount of patrol time assigned by supervisors to officers to spend in their beats and the amount of patrol actually received in each beat, as well as information on unallocated patrol time; and, (b) organized crime and total patrol information for the previous 5‐day reporting period based on level of crime and amount of patrol time reported by shift. Treatment at the hot spot level included reports specifically focused on each hot spot that were provided to commanders on a weekly basis. In the control condition, commanders received no information regarding where and for how long patrol units spend their time. Because there were two levels of treatment, analyses were performed at both the beat level and hot spot level. This study used a block randomized controlled trial design to assign 232 police beats to experimental (*N* = 116) and control conditions (*N* = 116). Of the total sample of 1,006 hot spots, 551 received treatment and 455 served as controls. Weisburd et al. (2015) estimated the impact of the intervention using trajectory group analysis and *F* tests that examined differences in crime between the control and treatment groups when the 13‐week intervention was active (March 22 to June 21, 2010).

Weisburd et al. (2015) found that results were generally consistent across the two levels of analysis. At the beat level, commanders assigned significantly more patrol time (17%) than commanders in the control group; however, disparities in total patrol time between the target and control groups were not significant and no noteworthy differences in crime were observed. At the hot spot level, more unallocated patrol time and total patrol time was spent at hot spots in treatment areas relative to control areas but the average patrol time assigned by commanders did not differ substantially. Treatment hot spots, however, did experience a notable 21% decrease in crime relative to control groups during the intervention.

### West Midlands Police's Randomized Control Trial of Crime Hot Spots

The West Midlands Police implemented a hot spots policing effort in the town of Perry Barr located in Birmingham, United Kingdom that explored the effects of varying lengths of time and frequencies of visits to hot spots on public‐generated antisocial behavior calls for service and street crime (Williams, 2015). Treatment hot spots received both 5‐ and 15‐min patrols and the duration of the patrol on a given day was determined at random (75 days received 5‐min patrols and 75 days received 15‐min patrols). Williams (2015) evaluated the intervention using a quasiexperimental design that paired the seven treatment hot spots to seven comparison hot spots that were selected at random from a pool of eligible untreated hot spots. The impact of the intervention was estimated by calculating changes in crime before, during, and after the intervention. Crime in spatial grids surrounding target areas was analyzed as a way of testing for displacement effects. Although the original plan was for the intervention to last 150 days, Williams (2015) noted that boundary adherence diminished considerably during the latter portion of the intervention; therefore, his analyses focused on the first 100 days of the intervention (June 2015 to September 2015).

A comparison of the two different patrol lengths found that less frequent but longer patrols were associated with slightly lower street crime and antisocial behavior compared to shorter but more frequent patrols (Williams, 2015). Collectively, hot spots that received either type of patrol treatment experienced a 14% reduction in street crimes and antisocial behavior calls for service compared to comparison hot spots. Evidence indicating the presence of a diffusion of benefits was also observed as crime declined in the areas immediately adjacent to treatment hot spots. Although crime decreased slightly in the treatment area, the cost‐benefit analysis completed by Williams (2015) found that the crime harm index actually increased as the result of the intervention.

### Actively Monitored CCTVs in Stockholm, Sweden

The City Police District in Stockholm began, in 2013, a 3‐year intervention that utilized CCTV cameras in and around two of the most violent locations in the country: Stureplan and Civic Square (Marklund & Holmberg, 2015). In Stureplan, seven cameras were installed and active between 11 p.m. to 6 a.m. In Civic Square, nine cameras were installed and were active between 9 p.m. and 5 a.m. Even though the cameras were active every night, they were only monitored on the weekends by an operator at the Stockholm County Communication Center. Marklund and Holmberg (2015) evaluated this approach using a quasiexperimental design where the two treatment sites were compared to a group of five other areas in the city with comparable levels of crime and social destinations (e.g., bars and restaurants). Crime incident data were analyzed from the 3 years before (July 2009 to March 2012) and during the intervention (July 2012 to March 2015) for six offenses: abuse, violence/threatening civil servant, unlawful threat, personal crime, sexual offense, and overall violence. A descriptive comparison was used to determine whether the implementation of the cameras and camera monitoring had any effects on the targeted crimes.

During the first 2 years, 15 and 22 developing incidents were disrupted as the result of active monitoring in the two target locations, respectively (Marklund & Holmberg, 2015). On the basis of crime counts when the cameras were actively monitored, the two targeted areas experienced reductions in total crime of 26% and 15% compared to the previous year with similar crime decreases when cameras were recording but not actively monitored. However, reductions in crime were greater in control areas during the intervention (28%) than in the two target areas. Marklund and Holmberg (2015) also found that changes in crime varied by type of offense. The most notable difference observed was for sexual offenses, which decreased 62% and 58% in the two target areas during the intervention whereas comparison areas experienced an 18% decrease.

Qualitative interviews with law enforcement personnel involved in the project revealed that there were several challenges encountered during the intervention (Marklund & Holmberg, 2015). For example, there were technical problems involving the cameras (pictures in black and white and cameras sometimes did not work) and quality issues were present in 14% to 15% of video reviewed. Furthermore, there was high turnover among camera operators which limited the ability for operators to gain experience and familiarity monitoring the cameras. One of the goals of implementing the CCTV cameras was that it would aid law enforcement in the investigation of the crimes that occur at those locations. However, camera footage was requested by investigators for only 20% of crimes that occurred within targeted locations. Additionally, there were only eight total cases between the two target locations that helped lead to criminal convictions.

### Operation Style in Peterborough, England

Operation Style sought to compare the effectiveness of the deployment of two different types of police personnel: “hard” patrols (sworn officers) and “soft” Police Community Support Officers (PCSC) patrols (civilian police staff who were not uniformed, did not carry weapons, and had limited arrest powers) (Ariel et al., 2016). The effectiveness of “soft” patrols at hot spots was evaluated using a randomized controlled trial design. Of the 72 identified hot spots, 34 received treatment and 38 were assigned to the control condition. Pre‐post differences in crime were analyzed using standardized mean differences and OLS regression. Crime displacement was examined in the 50‐m buffer zone around the targeted areas. GPS tracking data for all PSCOs and Constables were monitored and examined for interaction effects to ensure boundary adherence and treatment integrity. On average, targeted hot spots received two additional 10‐min visits per day from PSCOs than control hot spots.

Analyses indicated that targeted hot spots experienced decreases of 39% in crime and 20% in emergency calls for service compared to control hot spots (Ariel et al., 2016). Significant interaction effects were also observed. Both the number of visits and time spent at hot spots by PCSOs were associated with significant reductions in calls for service and crime incidents. Furthermore, Ariel et al. (2016) observed a “Reiss's Reward” effect where proactive patrols predicted less crime in target areas and reactive PCSO time predicted greater levels of crime in control areas. Overall, results support the presence of a spatial diffusion of benefits. When disaggregated by crime type, evidence of a diffusion of benefits was found for burglary, theft, criminal damage, robbery, and grievous bodily harm but evidence of displacement was found for sexual offenses and common assaults. Evaluation of the intervention using the Crime Harm Index suggested that each minute of PCSO patrol per day was associated with up to a 26‐day reduction in imprisonment in the treatment group relative to the control group. Ariel et al. (2016) did note that it was difficult to maintain the integrity of the originally intended treatment (three 15‐min patrols per shift) because of the irregular shape of the polygons that represented the hot spots created challenges when directing treatment toward the targeted locations.

### Glendale (AZ) Smart Policing Initiative

The Glendale Smart Policing Initiative (SPI) was a problem‐oriented policing strategy that utilized surveillance, targeted enforcement operations, and CPTED in an effort to reduce calls for service at convenience stores (Dario, 2016). Of the 65 convenience stores in Glendale, Arizona, 15 were Circle K stores and, in 2010, these 15 stores were responsible for 79% of calls for service at all convenience stores. Six Circle K convenience stores were selected to be the subject of the Glendale SPI. The response team proposed more than 220 CPTED recommendations to owners of the six targeted stores and recommended several other policy changes; however, even though several store managers followed some CPTED‐related suggestions, Circle K was largely nonresponsive of the recommendations and did not change their practices. In response to this inactivity, a working group was formed that shared findings with the media to publicly shame Circle K; this strategy proved effective in generating interest in the project among Circle K representatives (White & Balkcom, 2012).^23^

This quasiexperimental research design used two different control groups to examine the effects of the initiative: (a) the 13 remaining Circle K convenience stores; and (b) all 68 convenience stores in the city that did not receive treatment (Dario, 2016). Data spanning January 2008 to October 2013 were analyzed; during this period, the intervention was active from August 2010 to July 2011. Negative binomial random effects regression was used to estimate the impact of the intervention by comparing crime in pre‐ and postintervention periods (Dario, 2016). Crime in a 500‐yard buffer area surrounding targeted convenience stores was examined to test for potential spatial displacement.

Results from the difference‐in‐difference assessment showed a statistically significant reduction in calls for service at treatment stores when compared to either of the two control groups (Dario, 2016). Using all remaining convenience stores as the comparison group, regression results suggested a more than 16% reduction in calls for service postintervention for the treatment stores. When the comparison group was limited to only nontreated Circle K stores, reductions in calls for service were greater for the treatment stores relative to control stores but not by a significant margin. Evidence of a spatial diffusion of benefits was present for five of the six target stores. Importantly, Dario (2016) noted that the level of treatment likely varied by store but these differences were not able to be measured.

### Policing Violent Crime Hot Spots in Malmö, Sweden

Police in Malmö, Sweden began, in 2011, focusing on hot spots as a method of reducing violent crime with the aim of decreasing public physical assaults (Gerell, 2016). Of the 18 hot spots that were initially targeted, six clustered around a main entertainment area (Stortorget Square) and were assigned four additional patrol officers on weekend nights. On August 17, 2012, Malmö police added actively monitored CCTV cameras to increased patrol as part of their strategy to reduce crime in Stortorget Square. Law enforcement personnel would monitor the cameras and if a suspicious situation was developing, it would be communicated to patrol officers who could then intervene accordingly. The CCTV cameras were actively monitored between midnight and 6 a.m. on Saturday and Sunday nights. Gerell (2016) evaluated the effects of this strategy using a quasiexperimental design that compared assaults in Stortorget Square to a similar nightlife district elsewhere in the city that did not receive treatment. Reported assaults in the year CCTV cameras were active were compared to crime in the previous year. Temporal displacement was considered by examining crime counts in the hours and days that active CCTV monitoring was not taking place.

Statistical analyses did not find that actively monitoring CCTV cameras was responsible for reductions in public environment assaults. Public assaults in the treatment area declined by 20% during the intervention compared to the previous year but this decline was smaller than the 37% reduction observed in the control area (Gerell, 2016). It is important to note that prior to implementing the CCTV component of the broader hot spot policing strategy, the target district experienced a decrease in public environment assaults from 2011 to 2012 when only increased police presence was being used. When limiting analyses to only times and days when CCTV cameras were actively monitored, there was a decrease in public assaults in favor of treatment whereas control times experienced a nonsignificant increase in public assaults.

### Operation Impact in New York City (NY)

Operation Impact in New York City is a well‐known program for the NYPD that began in 2003 and was carried out for over a decade (MacDonald et al., 2016). The crux of the program consisted of directed patrol and intensive investigative stops in high crime zones. Initially, 24 impact zones were targeted but from 2004 to 2012, 75 of the city's 76 precincts had at least one impact zone. A quasiexperimental difference‐in‐difference study design was used to examine the effects of increased police patrols on arrests and crime (MacDonald et al., 2016). Weekly data from 2004 to 2012 were aggregated to the census block group level. Impact zones were compared to other areas in the same precinct that did not receive treatment. The authors analyzed weekly crime data from 2004 to 2012 aggregated to the census block group level and estimated the impact of the intervention using Poisson regression models with fixed effects for each precinct‐month‐year. Statistical models also explored 2‐month leads and lags. Possible spatial displacement was assessed by including controls for block groups adjacent to impact zones.

Overall, impact zones were associated with a 12% reduction in expected monthly total crime (MacDonald et al., 2016). Results varied when disaggregating by type of crime. Impact zones experienced significant decreases in total crime, assault, burglary, drug crime, misdemeanor offenses, felony property crime, robbery, and felony violent crime. Treatment of Impact Zones also significantly increased the number of arrests overall, as well as the number of arrests for burglary, weapons offenses, misdemeanors, and felony property crime. When including lags and leads, significant reductions were observed for burglary, robbery, and property felony offenses in favor of the treatment zones but there were significant increases in weapons and other felony offenses. In terms of dosage, greater numbers of probable cause stops when impact zones were operational were related to lower levels of crime. MacDonald et al. (2016) cautioned that these results have little practical importance as the number of probable cause stops would have to increase substantially in order to prevent one crime. Lastly, crime in areas adjacent to impact zones experienced an overall monthly crime decline of 7% suggesting a diffusion of benefits.

### Kansas City (MO) Foot Patrol Project

Following the model set forth by the Philadelphia Foot Patrol Experiment, the Kansas City Foot Patrol Project used increased, targeted foot patrol in an effort to reduce violent crime (Novak et al., 2016). Rookie officers were assigned to foot patrol shifts Tuesday through Saturday over a 90‐day period. Novak et al. (2016) evaluated the initiative using a quasiexperimental design where the four treatment police beats matched to four control police beats. Outcomes were measured bi‐weekly for 83 weeks resulting in a total of 336 observations. Panel‐specific autoregressive models (PSAR(1)) were used to examine changes in bi‐weekly crime counts in the 30 weeks before the intervention to the 90‐day intervention and 40‐week postintervention period. Displacement effects were investigated by examining changes in crime in roughly two‐block areas surrounding the target zones.

Both pre‐post and preactive intervention period comparisons of bi‐weekly crime counts favored treatment but not by a significant margin (Novak et al., 2016). There was evidence of a spatial diffusion of benefits as crime in catchment areas decreased significantly when comparing pre‐ and postintervention time periods. Novak et al. (2016) found the intervention to be more effective in its earlier stages. In the first 30 days of the intervention, there was a significant reduction in violent crime in favor of the treatment group without evidence of crime displacement. Conversely, in the last 45 days, no significant differences in violent crime were observed for the treatment group relative to the control group.

### Police Paramilitary Unit Raids in Buffalo (NY)

In 2012, the Buffalo Police Department executed 39 police paramilitary raids throughout the city over a 2‐day period at locations known for drug activities and recent violent crime (Phillips et al., 2016). This effort was carried out under presumption that these highly visible raids would produce a general deterrent effect and, as a result, reduce crime (e.g., calls for service, Part I violent crime, and Part I nonviolent crime). Phillips et al. (2016) evaluated the effects of this strategy using a quasiexperimental design with a comparison group constructed via propensity score matching. Of the 384 total areas used in this analysis, 99 received treatment and 285 served as controls. The impact of the intervention was estimated using a fixed effects negative binomial panel model that compared pre‐post changes in crime for both the target and control areas.

Results offered little support for the short‐term application of police paramilitary raids as a broader crime reduction strategy. Both calls for service and drug arrests increased significantly in target areas following the raids relative to control areas whereas Part I violent and Part I nonviolent crime decreased but not significantly (Phillips et al., 2016). Phillips et al. (2016) explored possible decay effects by examining Part I crime by week in the 5 weeks following the raids. Part I crime declined in both of the first 2 weeks after the raids but not to a significant degree.

### Offender‐Focused Police Intervention at Hot Spots in Port St. Lucie (FL)

In 2013, Port St. Lucie PD launched an offender‐focused hot spot policing strategy that sought to reduce residential burglary and theft from vehicles (Santos & Santos, 2016). The evaluation of this strategy followed a block randomized controlled trial design where 48 hot spots (stratified by crime per offender rate) were randomly assigned to the treatment (*N *= 24) or control condition (*N *= 24). For each target location, a list was compiled of offenders living in the area who have been arrested for residential burglary and theft from vehicle crimes, were on probation for a prior burglary arrest at the time, and nonviolent convicted offenders on probation for felony drug crime. After identifying these individuals, the crime analyst provided detectives working in the area with each individual's “criminal résumé” which contained a “comprehensive criminal and corrections history; any contacts made with the police department, as a victim, a witness, in a call for service, or in a traffic citation; a list of the targeted offender's associates; residence history; credit history; history with city services (e.g., utilities, code enforcement); and social media activity (e.g., Facebook, Twitter, Instagram)” (Santos & Santos, 2016, p. 381). Two detectives carried out the treatment, each assigned to 12 of the 24 targeted areas, made regular contact with the identified offenders over the 9‐month intervention. The effectiveness of the offender‐focused strategy was assessed using negative binomial and ordinary least squares regression that compared outcomes from before the intervention (October 2012 to June 2013) to when the intervention was active (October 2013 to June 2014).

Initial *t* test results found there were 46% fewer targeted offenders arrested in treatment areas during the intervention than preintervention and 68% fewer arrests per targeted offender, and these differences were significant at the *p* < .05 level (Santos & Santos, 2016). For hot spot crime, regardless of whether the treatment effect was modeled as binary or in terms of dosage (the number of contacts made), results did not indicate that treatment was related to residential burglary or theft from vehicle crime though the direction of the coefficient was in the hypothesized direction. Similar results were observed when examining the impact of the intervention on other outcomes: both measures of treatment were associated with fewer arrests and rearrests but neither to a significant degree.

### New Haven (CT) Smart Policing Initiative

In response to the city's most violent year since the mid‐1990s, the New Haven Smart Policing Initiative (SPI) was implemented in 2013 in one of the city's most violent neighborhoods: Newhallville (Sedelmaier & Hipple, 2016). The 13‐week effort relied primarily on increased foot patrol as a crime reduction tactic but this was supplemented with problem‐oriented policing and community engagement. Whether this approach was effective at reducing total crime and violent crime was assessed using a quasiexperimental design where the target area was compared to four other neighborhoods in the city with comparable levels of violent crime, population size, poverty levels, and racial composition. Sedelmaier and Hipple (2016) conducted analyses at two different areal levels: the neighborhood and high‐risk areas within neighborhoods that were identified via risk terrain modeling. Crime counts in three separate 13‐week periods were compared: before, during, and after the intervention. Importantly, Sedelmaier and Hipple (2016) noted that the early stages of the intervention suffered from a high degree of turnover among supervisors involved in the project but this issue was eventually settled and the implementation of the intervention was able to proceed smoothly.

Overall, results indicated greater crime control gains in the treatment neighborhood relative to comparison neighborhoods. At the neighborhood level, a pre‐post comparison showed Newhallville experienced declines of 33% for total crime and 52% for violent crime (Sedelmaier & Hipple, 2016). The four comparison neighborhoods also experienced reductions in total crime but to a lesser degree than the target neighborhood (17% to 38%). Furthermore, two comparison neighborhoods experienced slight reductions in violent crime and two experienced an increase in violent crime. Comparable results were observed when focusing only on high‐risk areas within neighborhoods. Reductions in total crime and violent crime at high‐risk areas within the target neighborhood were larger than reductions in crime at high‐risk areas in comparison neighborhoods, and some high‐risk comparison areas actually experienced an increase in violent crime.

### Operation Menas in London, England

Operation Menas used a randomized controlled trial design to examine the effects of increased visible patrol at bus stops (with a 50‐m buffer) on victim‐generated calls for emergency and bus driver incident reports (DIRs) (Ariel & Partridge, 2017). The specific aspects of the treatment included a double patrol team of uniformed officers visiting targeted hot spots three times per day for 15 min, and this occurred five times per week over the 6‐month intervention period. Bus driver incident reports were examined for three different time periods: when the patrols were active (Monday–Friday, 12:00 to 18:00), hours when the patrols were not active, and all hours for all days. Victim‐generated calls for service were only examined for the time when patrols were active (Monday–Friday, 12:00 to 18:00). The unit of analysis for this study was the individual bus stop with a 50‐m buffer. Two separate catchment areas around individual bus stops were used to explore crime displacement: 50–100 m and 100–150 m. A total of 102 bus stops were randomly assigned to treatment (*N *= 51) and control groups (*N* = 51). Differences in crime between pre‐ and postintervention periods were assessed using a generalized linear regression model with an adjusted Poisson distribution created by the Pearson *χ* ^2^ scale parameter method to account for over‐dispersion.

Results from this study were mixed. A marginally significant 37% reduction in DIRs was observed during active treatment hours in the immediate vicinity of treated bus stops relative to control groups (Ariel & Partridge, 2017). In contrast, treated bus stops experienced a nonsignificant (*p* = .10) increase of 25% in victim‐generated crime counts compared to the control bus stops. Assessments of spatial displacement of crime found the closest catchment area (50–100 m) experienced a statistically significant 40% decrease in DIRS and a marginally significant 23% increase in victim‐generated crimes during active intervention hours. No significant pre‐post changes in DIRs or victim‐generated crimes were observed in the farthest catchment area (100–150 m). Analyses revealed slight evidence of a temporal diffusion of benefits as DIRs declined 29% (*p* < .10) when treatment patrols were not active during the intervention period.

### Investigating Hot Spot Policing in Copenhagen, Denmark

In 2015, the National Police in Denmark initiated a problem‐oriented hot spots policing effort in three police districts in an effort to reduce street crime (Attermann, 2017). Specific tactics deployed by the team executing the intervention included increased visible patrol, removal of signs of physical disorder, installation and monitoring of surveillance cameras, support for private security guards and business owners, information campaigns geared toward vulnerable populations, and making contacts with people identified as at‐risk for engaging in crime. There were a total of 31 hot spots, ranging from a shopping mall to individual bars or stores, included in the trial across all three police divisions. A block randomized controlled design, stratified by crime and features of the environment, was used to place hot spots into treatment (*N* = 15) and control conditions (*N* = 16). The effectiveness of the intervention was assessed using a difference‐in‐difference design that compared crime during the intervention (January 15, 2016 to September 15, 2016) to the same period in the previous year (January 15, 2015 to September 15, 2015). Importantly, Attermann (2017) emphasized that crime is relatively low in Denmark and that even among the identified hot spots, crime was less concentrated compared to a typical hot spot in the United States.

There were 71 total treatment activities carried out in the targeted areas: eight were already taking place before the start of the intervention and were not modified, 26 were relaunched activities where strategies were in place previously but were modified after the intervention was initiated, and 37 were newly established activities (Attermann, 2017). Each of these activities were initiated at varying points during the intervention period.

Difference‐in‐difference results indicated that the intervention had differing effects across the various crime types that were considered (Attermann, 2017). Treatment was associated with significant decreases in vandalism and motor vehicle crime relative to control areas but was unrelated to all other outcomes (violence; violence, robbery, and threats; shoplifting; other theft; and total street crime). Attermann (2017) performed a subgroup analysis of the nine hot spots that received the most intensive treatment and found treatment intensity was unrelated to street crime overall and during targeted times of day.

### Hot Spots Policing in Bogotá, Colombia

The Mayor's Office in Bogotá, Colombia implemented an intensified state presence effort with the aim of reducing crime at high‐crime streets (Blattman et al., 2017). There were 1,919 street segments randomly assigned to one of four experimental conditions: (a) doubled policing patrol, (b) increased municipal service, (c) both increased patrol and municipal service, or (d) control. A two‐stage block (by police station) randomization procedure was used. First, quadrants were randomly assigned to treatment or control then hot spots within the quadrants were randomly assigned to treatment and control. No additional officers were hired to execute the intensive patrol strategy that spanned February 2016 to October 2016; rather, officers were only directed to increase the time spent at targeted high crime street segments where they carried out normal police activities. Increased municipal services were implemented from June 2016 to August 2016; however, before and after pictures of streets showed little difference following beautification treatment. The impact of the intervention was estimated using weighted least squares regression while accounting for randomization interference and included inverse probability weights. Potential crime displacement was assessed by examining crime in street segments within 250 meters of targeted street segments.

Analyses of only on the directly treated hot spots showed doubled police patrol and, separately, increased municipal service treatments were associated with significant reductions in crime and people's perceived security risks (Blattman et al., 2017). However, when accounting for interference between units, the size of treatment effects become more modest. Specifically, <100 crimes were prevented by all treatment conditions over the 8‐month intervention period. The largest reductions in crime and people's perceived security risks were observed for hot spots that received both forms of treatment. Survey results indicated that the intervention did not change the level of trust that residents had in the state but it was associated with people having less favorable opinions of the Mayor's office. Notably, Blattman et al. (2017) found a sizable crime increase in street segments within 250 m of segments that received intensive policing and this increase was larger than the direct treatment crime reduction benefits. Property crime was most likely to be displaced whereas there was limited and inconsistent evidence of a diffusion of benefits for violent crime. Lastly, the increased municipal services treatment condition was associated with a small diffusion of benefits into nearby street segments.

### The Philadelphia (PA) Predictive Policing Experiment

The Philadelphia Predictive Policing Experiment was a 90‐day intervention that tested the effectiveness of three different policing strategies at crime hot spots that were identified using a risk‐based algorithm to predict high crime locations (Ratcliffe et al., 2017). A randomized controlled trial design was used to randomly assign 20 police districts to one of four conditions: (a) control; (b) awareness; (c) awareness with dedicated marked patrol car and uniformed officers; or (d) awareness with dedicated officers and an unmarked vehicle.^24^ Each police district had three predicted mission areas (500 by 500 feet grids) and the unit of analysis was the district‐day. The authors used a null mixed effects negative binomial regression model with a random effect for police district to estimate the impact of the intervention on property crime and a descriptive comparison for violent crime. Displacement effects were explored by examining crime in grids adjacent to the target and within two cells of each predicted grid. Temporal displacement was assessed by tracking crime counts in an 8‐hr period following the treatment shift.

Analyses suggested the effectiveness of police efforts at crime hot spots was contingent upon the tactic deployed. The “awareness with uniformed patrol” treatment was associated with a 31% decline in property crime and produced evidence of a temporal diffusion of benefits in the form of 42% less property crime in the 8 hr after the treatment shift (Ratcliffe et al., 2017). In contrast, neither the “awareness only” or “awareness with unmarked patrol” strategies were related to property crime and none of the three treatment conditions were related to violent crime. From a statistical analysis standpoint, Ratcliffe et al. (2017) noted that limited statistical power impaired their ability to detect significant differences in the models.

### Flint (MI) DDACTS Pilot Program

A shrinking city budget that was the product of rapid outward population migration led to a nearly 50% reduction in Flint PD officers from 2003 to 2011 (Rydberg et al., 2017). In an effort to counter the drastically reduced police force, Michigan State Police (MSP) assigned officers to conduct directed patrols in high violent crime areas in Flint, as well as two other Michigan cities (Detroit and Saginaw). These supplemental MSP patrols formed the Flint DDACTS Pilot Program which sought to reduce violent crime, including homicide, aggravated assault, and robbery.

Rydberg et al. (2017) noted two ways in which the Flint DDACTS program differed from typical DDACTS initiatives: (a) violent crime was the primary determinant of hot spot identification rather than the overlap of crime and traffic accidents and (b) the areas targeted were much larger than what is typically considered a hot spot. Importantly, although the intervention was implemented at a larger areal unit, the analysis was performed at the block level. A quasiexperimental design with comparison blocks constructed via synthetic control modeling was used to evaluate the effects of this intervention. In the seven targeted hot spots, there were 1,117 blocks exposed to the DDACTS treatment. Two different comparison groups were used: (a) blocks in Flint that did not receive the treatment (*N* = 1,888) and (b) blocks in the similar city of Detroit where DDACTS was not present (*N* = 13,097). Because seven different hot spots were targeted, each beginning at different times and lasting for varying periods, Rydberg et al. (2017) modeled the treatment as seven different interventions. The overall impact of the strategy on violent crime was estimated using a fixed effects meta‐analysis that compared quarterly crime counts preintervention (January 2010 to December 2011) to quarterly crime counts during the intervention (January 2012 to December 2013).

Results from the meta‐analysis offered little support for the Flint DDACTS program as an effective violent crime reduction strategy with some evidence suggesting that areas exposed to treatment experienced an increase in violent crime (Rydberg et al., 2017). Specifically, when using the Flint comparison blocks, the intervention was associated with significant increases in overall violence (18%) and aggravated assault (33%). A similar aggravated assault increase among treated blocks (26%) was observed when using the Detroit comparison blocks though this effect declined over time. Notably, with Detroit blocks as the comparison group, robberies were 24% lower in the treatment blocks at a statistically significant level.

### Operation Strikeforce in Buffalo (NY)

From April to May of 2013, Buffalo PD conducted a series high visibility roadblocks that used an automated license plate reader to scan the license plates of all passing vehicles in order to see whether the driver had an outstanding warrant or revoked license or if the vehicle was stolen (Wheeler & Phillips, 2018). During this 2‐month period, 60 roadblocks were performed at 46 separate locations. Wheeler and Phillips (2018) assessed the effectiveness of this approach using a quasiexperimental design that paired treated street segments (*N *= 328) with comparison street segments (*N *= 328) via propensity score matching. *t* tests and fixed effects negative binomial regression were used to estimate the impact of the intervention by comparing pre‐post crime counts.

Overall, results were mixed as to whether the intervention effectively reduced crime (Wheeler & Phillips, 2018). Results from *t* tests showed that treatment street segments had significantly less Part I violent crime after the intervention but significantly more traffic accidents. Multiple regression analyses suggested that roadblock locations experienced significant increases in Part I violent and Part I nonviolent crime, but significantly fewer traffic accidents. Wheeler and Phillips (2018) also deconstructed the postintervention period and explored possible decay effects over the 4 weeks after the intervention. This supplemental analysis, however, yielded inconsistent results.
